# Effects of (Pro)renin Receptor on Diabetic Cardiomyopathy Pathological Processes in Rats via the PRR-AMPK-YAP Pathway

**DOI:** 10.3389/fphys.2021.657378

**Published:** 2021-05-27

**Authors:** Jie Xiong, Xuefei Dong, Shengnan Li, Fan Jiang, Jing Chen, Shiran Yu, Bo Dong, Qing Su

**Affiliations:** ^1^Department of Cardiology, Shandong Provincial Hospital Affiliated to Shandong University, Jinan, China; ^2^The Key Laboratory of Cardiovascular Remodeling and Function Research, Chinese Ministry of Education, Qilu Hospital of Shandong University, Jinan, China; ^3^Department of Sport, Health and Exercise Science, University of Hull, Hull, United Kingdom; ^4^Warwick Medical School, University of Warwick, Coventry, United Kingdom; ^5^Department of Endocrinology, Xinhua Hospital, Shanghai Jiao Tong University School of Medicine, Shanghai, China

**Keywords:** diabetic cardiomyopathy, (pro)renin receptor, Yes-associated protein, AMPK, myocardial fibrosis, inflammation

## Abstract

Diabetic cardiomyopathy (DCM) is a common complication associated with diabetes. The (pro)renin receptor (PRR) is an important member of the local tissue renin-angiotensin system and plays a vital role in many cardiovascular diseases. Yes-associated protein (YAP) also plays a crucial role in many cardiovascular diseases. However, the mechanism responsible for the effects of PRR and YAP on DCM remains unclear. The purpose of this study was to determine the role of PRR in the pathological progression of DCM and whether PRR influences the pathological processes of diabetic cardiomyopathy through YAP. We first established diabetic cardiomyopathy rats model, downregulated the expression of PRR, and upregulated and downregulated the expression of YAP. The levels of myocardial inflammation and fibrosis were then measured and cardiac function was evaluated. In vitro, primary rat cardiac fibroblasts (CFs) were cultured with high glucose, with or without transfection with recombinant adenovirus expressing PRR, and GSK621 was used to observe the effect of AMPK. The levels of inflammation and fibrosis were measured in vitro. The results showed that PRR and YAP silencing alleviated myocardial inflammation and fibrosis. GSK621 blocked the effect of PRR on AMPK and YAP and improved CF inflammation and fibrosis. The inhibition of PRR expression offers a new therapeutic strategy for the treatment of DCM. The effects of PRR on the pathological process of DCM in rats may be mediated via the PRR-AMPK-YAP pathway.

## Introduction

Diabetic cardiomyopathy (DCM) is a common complication associated with diabetes. Due to its insidious onset, rapid development and poor prognosis, it has attracted increasing amounts of attention (Tan et al., 2019). In diabetic cardiomyopathy, the abnormal proliferation of cardiac fibroblasts and the deposition of extracellular matrix (ECM) aggravate the pathological process of cardiac fibrosis, thereby affecting cardiac function (Tao et al., 2019; Arow et al., 2020). Although there are many studies on the development of diabetic cardiomyopathy, the underlying pathological mechanism of diabetic cardiomyopathy is still not clear.

The pro(renin) receptor (PRR) is a specific receptor of renin, which encodes a 350 amino acid protein with a single transmembrane domain, and it has no homology with any other known membrane protein. The combination of PRR and renin improves the catalytic efficiency of the conversion of angiotensin to angiotensin I four-fold (Nguyen et al., 2002). The activity of PRR emphasizes the role of the cell surface in angiotensin II generation and opens new perspectives on the tissue renin-angiotensin system (RAS). Moreover, the role of PRR independent of the RAS has received increasing attention in recent years. Furthermore, some studies have reported that PRR is involved in diabetic and hypertensive end-organ damage and some researchers proposed that PRR was a pivotal link between the pathogenesis of diabetes mellitus and end-organ damage (Inagami et al., 2008; Connelly et al., 2011). So we suspected that PRR may play an important role in the pathogenesis of diabetic cardiomyopathy.

The transcriptional co-activator Yes-associated protein (YAP) is a major downstream effector of the Hippo pathway. The Hippo pathway, originally identified in Drosophila, is attracting intense attention as a key regulator of development, organ size, tissue regeneration and tumorigenesis (Hao et al., 2017). However, recent studies have also reported a strong association between YAP and cardiovascular disease. One study showed that YAP levels were elevated in the heart samples from patients with hypertrophic cardiomyopathy and in mice with transverse aortic constriction (TAC) (Wang P. et al., 2014), suggesting the role of nuclear YAP in hypertrophic heart disease. Another study showed elevated levels of phosphorylated YAP in heart samples from patients with ischemic or non-ischemic heart failure compared with patients with nonfailing heart samples (Leach et al., 2017), suggesting that YAP also plays an important role in heart failure. There is a study that reported that YAP, a key downstream transcriptional cofactor in the Hippo signaling pathway, is present in the mammalian heart and it plays an important role in regulating the growth and death of cardiomyocytes (CMs). Increasing lines of evidence also suggest that YAP promotes regeneration of the heart after myocardial infarction (Ikeda and Sadoshima, 2016). Moreover, there was a study that found that YAP plays a crucial role in cardiac dysfunction during pressure overload (Ikeda et al., 2019). Although there are many lines of evidence suggesting that YAP is closely involved in cardiovascular disease, it is still not clear whether YAP is involved in the pathological process of diabetic cardiomyopathy.

Recent research has found that AMPK plays an important role in maintaining cardiovascular processes and inhibiting disease progression. A study showed that aberrant AMPK signaling is involved in the pathogenesis of atherosclerosis, hypertension coronary heart disease and cardiovascular complications of diabetes mellitus (Wu and Zou, 2020). Previous study showed that PRR could regulate the phosphorylation of AMPK (Akhtar and Siragy, 2019).

In view of the relationship between PRR, YAP and cardiovascular diseases, we hypothesized that PRR and YAP may be involved in the development of DCM through PRR-AMPK-YAP Pathway. Therefore, we designed and conducted a series of experiments to verify our hypothesis that PRR and YAP may aggravate the pathological process of DCM and lead to further cardiac dysfunction.

## Materials and Methods

### Recombinant Adenoviruses Expressing PRR

An open reading frame (ORF) for PRR gene amplification was constructed by GenePharma Company (Shanghai, China) and the ORF was inserted into the pDC316 plasmid vector to construct pDC316-PRR. Then, the pDC316-PRR plasmid or pDC316-EGFP, and an adenovirus vector, were co-transformed into *Escherichia coli* to produce recombinant adenoviruses expressing PRR (Ad-PRR) or EGFP (Ad-EGFP).

### Recombinant Adenoviruses Expressing shRNA Targeting PRR

Short hairpin RNA (shRNA) fragments corresponding to PRR were constructed and introduced into recombinant adenoviruses by GenePharma Company (Shanghai, China) to form adenoviruses-coated PRR shRNA fragments (Ad-PRR-shRNA). The sequence of shRNA targeting the rat PRR refers to our previous research (Xiong et al., 2019). Scramble-shRNA coated by adenoviruses (Ad-SC-shRNA) served as a negative control.

### Recombinant Adenoviruses Expressing YAP

The amplified open reading frame (ORF) of the YAP gene designed by the Genechem Company (Shanghai, China) was constructed and then used to create recombinant adenoviruses that express YAP (Ad-YAP). Recombinant adenoviruses (Ad-EGFP) expressing EGFP were used as a negative control.

### Recombinant Lentivirus Expressing shRNA Targeting YAP

Short hairpin RNA (shRNA) fragments corresponding to YAP were prepared and introduced into a recombinant lentivirus by Genechem Company (Shanghai, China) to form a lentivirus-coated YAP shRNA fragment (LV-YAP-shRNA). The sequence of the shRNA targeting the rat YAP 3’-UTR was 5’-GCTGCCACCAAGTT-3’ according to study by Wang et al. (2012). Scramble-shRNA coated by lentivirus (LV-SC-shRNA) served as a negative control.

### Animal Model

120 male 8-week-old Wistar rats (200 ± 12 g) obtained from Shandong University Animal Center were maintained in a room with an ambient temperature of 25 ± 2°C and 50 ± 10% humidity and free access to standard rodent chow and water, under a 12 h light and 12 dark cycle. The animals were used in the experiments after a 1-week acclimatization period.

For the PRR silencing experiment, the rats were randomly divided into four groups: control group (*n* = 15), DCM group (*n* = 15), DCM+Ad-SC-shRNA group (Ad-SC-shRNA group, *n* = 15), and DCM+Ad-PRR-shRNA group (Ad-PRR-shRNA group, *n* = 15). After fasting for over 12 h, the rats were given a single intraperitoneal injection of streptozotocin (STZ) (65 mg/kg) to construct a diabetes model. Wistar rats with blood glucose levels > 11.1 mmol/L and typical clinical symptoms a week after the injection were considered diabetic rat models. The rats in the Ad-SC-shRNA group and Ad-PRR-shRNA group were injected with 1 × 10^9^ PFU of either the Scramble-shRNA or PRR-shRNA recombinant adenoviruses (dissolved in 100 μl normal saline) per rat via the tail vein twelve weeks after the injection of STZ. Two weeks after the adenovirus injection, 4 rats were randomly selected from each of the two groups to measure the efficiency of PRR silencing, and the remaining rats were used for the subsequent experiments. Four groups of rats, including the control group, DCM group, Ad-SC-shRNA group, and Ad-PRR-shRNA group were euthanized under anesthesia (intraperitoneal injection of 10% chloral hydrate, 300 mg/kg) 4 weeks after the injection of recombinant adenovirus. All of the rat hearts were collected for further experiments.

For the YAP overexpression experiment, the rats were randomly divided into four groups: control group (*n* = 15), DCM group (*n* = 15), DCM+Ad-EGFP group (Ad-EGFP group, *n* = 15), and DCM+Ad-YAP group (Ad-YAP group, *n* = 15). The establishment of the diabetic rat model was the same as above. The rats in the Ad-EGFP group and Ad-YAP group were injected with 1 × 10^9^ PFU of either the Ad-EGFP or Ad-YAP recombinant adenoviruses (dissolved in 100 μl normal saline) per rat via the tail vein twelve weeks after the injection of STZ. The detection of viral transfection efficiency and subsequent experimental operations were the same as that of the PRR silencing experiment.

For the YAP silencing experiment, the animals were randomly divided into the following four groups: control group (*n* = 15), DCM group (*n* = 15), DCM+Scramble-shRNA group (LV-SC-shRNA, *n* = 15), and DCM+YAP-shRNA group (LV-YAP-shRNA, *n* = 15). The establishment of the diabetic rat model was the same as above. The rats in the LV-SC-shRNA group and the LV-YAP-shRNA group were injected with 1 × 10^6^ PFU of either the Scramble-shRNA or YAP-shRNA recombinant lentivirus (dissolved in 100 μl normal saline) per rat via the tail vein. Four weeks after the lentivirus injection, 4 rats were randomly selected from each of the two groups to measure the efficiency of YAP silencing, and the remaining rats were used for subsequent experiments. The subsequent experimental operations were the same as that for the PRR silencing experiment.

All of the animal experiments protocols were approved by the Animal Care and Use Committee of Shandong Provincial Hospital, Shandong University and were conducted in accordance with the National Institutes of Health Guidelines for the Care and Use of Laboratory Animals. All of the animal studies have been performed in accordance with the ethical standards laid down in the 1964 Declaration of Helsinki and its later amendments.

### Histology and Morphometric Analyses

The myocardial tissue was paraffin-embedded and sliced into 4.5-μm-thick histological sections for subsequent experiments. Some of the sections were stained with hematoxylin and eosin (HE) as well as subjected to Masson trichrome staining. The sections were stained with Masson’s trichrome and the collagen components were quantitated by measuring the proportion of area positively stained with Masson’s trichrome to the total area in the section.

### Histopathology and Immunohistochemistry

Firstly, the histopathological sections were incubated overnight at 4°C with primary antibodies against rat PRR (1:100, Abcam, United Kingdom), YAP (1:200, Cell Signaling Technology, United States), Collagen I (1:200, Abcam, United Kingdom), Fibronectin (1:200, Abcam, United Kingdom), PAI-1 (1:100, Affinity, United States), TGF-β (1:100, Affinity, United States), IL-1β (1:200, Abcam, United Kingdom), IL-18 (1:100, Affinity, United States), profilin-1 (1:100, Affinity, United States), IL-8 (1:200, Abcam, United Kingdom), and TNF-α (1:100, Affinity, United States). Negative controls were treated with PBS. The sections were heated at 37°C for 30 min, then incubated with a horseradish peroxidase-labeled secondary antibody (ZSGB-Bio, China) for 20–30 min at 37°C. Finally, the sections were examined under a confocal FV 1000 SPD laser scanning microscope (Olympus Corporation, Tokyo, Japan). Quantification of immunohistochemical indicators and the collagen components with Masson’s trichrome involved use of an automated image analysis system (Image-Pro Plus 5.0; Media Cybernetics), and the positively stained area was calculated as mean percentage of the total area.

### Echocardiography Analysis

At the end of the experiment, the rats in the control group, DCM group, Ad-EGFP group, Ad-YAP group, LV-SC-shRNA group and LV-YAP-shRNA group were examined by echocardiography after anesthesia. Echocardiography was performed with a VEVO 770 echocardiography system equipped with a 25 MHz transducer (Visual Sonics, Toronto, Canada). M-mode signals were collected, and left ventricular functions were measured.

### Cell Culture

Primary rat neonatal cardiac fibroblasts (CFs) were isolated from 1- to 3-day-old Wistar rats according to the methods described in our previous report.

To determine the effect of PRR gene overexpression on the inflammation and fibrosis of in CFs, the cells were divided into four groups: pure High Glucose group, Ad-EGFP group, Ad-PRR group and Ad-PRR+GSK621 (AMPK agonist) group. The cardiac fibroblasts (2.5 × 10^4^ cells) were incubated in the presence of Ad-PRR or Ad-EGFP at 150 multiplicities of infection[MOI]. The Ad-EGFP group, Ad-PRR group and Ad-PRR+GSK621 group were infected with Ad-EGFP, Ad-PRR and Ad-PRR respectively [MOI = 150]. The cells were cultured in normal medium for another 12 h after 12 h of transfection. The Ad-PRR+GSK621 group was pre-treated with the specific AMPK agonist GSK621 (30 μM, Selleck, United States) in normal medium for 1 h. Then, the cells were stimulated in high glucose (25 mM) with GSK621 (30 μM) for 48 h (Sujobert et al., 2015).

### Western Blot Analysis

Total protein was extracted from myocardial tissue and fibroblasts. The total protein concentration was determined, and the proteins were separated by SDS-PAGE and then transferred to polyvinylidene difluoride membranes. After blocking nonspecific binding in 5% skim milk for 1.5 h, the membranes were incubated overnight at 4°C with the following primary antibodies: anti-pAMPK (1:1000, Affinity, United States), anti-AMPK (1:1000, Affinity, United States), anti-PRR (1:1000, Abcam, United Kingdom), anti-GAPDH (1:1000, Abcam, United Kingdom), and anti-YAP (1:1000, Cell Signaling Technology, United States). Then, horseradish peroxidase-conjugated secondary antibodies (1:5000, ZSGB-bio, China, ZB-2301) were used to bind to the specific antigen-antibody complexes. The densitometry of the bands was performed with ImageJ (National Institutes of Health, Bethesda, MD, United States).

### Real-Time PCR

Total RNA was isolated from the myocardial tissues and cardiac fibroblasts by using TRIzol Reagent (Invitrogen, United States). The extracted RNA was diluted in DEPC water and the concentration of extracted RNA was determined by OD260 measurements. Using Super ScriptIII (Takara, Japan) to reverse-transcribe the total RNA to cDNA. Then, cDNA was amplified using SYBR Green (Takara, Japan). And using the 2^–ΔΔCT^ method to calculate the relative gene expression.

### Enzyme Linked Immunosorbent Assay (ELISA)

The cell supernatant from the cells of the above four groups was collected for ELISA to detect the expression of Collagen I, Collagen III, IL-1β and IL-6. All measurements were performed according to the instructions of the manufacturers of the ELISA Kits. The OD value of each sample was determined at 450 nm using an Enzyme Standard Instrument (Thermo Fisher Scientific, United States) and used to calculate the concentration of the protein in the medium.

### Statistical Analysis

Statistical analyses were evaluated using GraphPad Prism 7.0 software (GraphPad Software, La Jolla, United States). All data are expressed as the mean ± standard deviation of at least three independent experiments. The differences were analyzed using one-way ANOVA. Multiple comparison between the groups was performed using S-N-K methods.

## Results

### PRR Silencing Decreased PRR and YAP Expression and Increased the Phosphorylation of AMPK in Rats With Diabetic Cardiomyopathy

**FIGURE 1:**
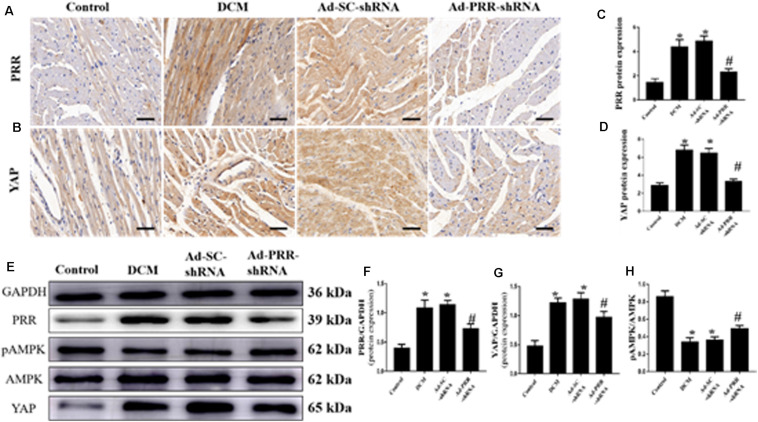
PRR silencing decreased PRR and YAP expression and increased the phosphorylation of AMPK in rats with diabetic cardiomyopathy (*n* = 5). **(A,B)** Representative immunohistochemical staining of PRR and YAP in the 4 groups (scale bar = 20 μm). **(C,D)** Quantitative analysis of the YAP protein expression shown in panels **(A,B)**. **(E)** western blot analysis of PRR, pAMPK, AMPK, and YAP protein expression in the 4 groups. **(F,G)** Quantitative analysis of the PRR and YAP protein expression shown in panel **(E)**. **(H)** Quantitative analysis of the pAMPK/AMPK shown in panel **(E)**. *p < 0.05 versus the control group; ^#^*p* < 0.05 versus the Ad-SC-shRNA group.

PRR and YAP expression in all of the rat groups was evaluated by immunohistochemical staining and western blotting. The immunohistochemical staining results showed that YAP protein expression in the DCM group was significantly upregulated compared to that in the control group; however, this upregulation was significantly alleviated in the Ad-PRR-shRNA group (Figures 1A–D *p* < 0.05). The expression of the PRR and YAP proteins, as detected by western blot, were consistent with the results of the immunohistochemical staining (Figures 1E–G *p* < 0.05). Furthermore, the western blot results showed that the phosphorylation of AMPK (pAMPK) was decreased in the DCM group compared with the control group, and PRR gene silencing increased pAMPK expression in the Ad-PRR-shRNA group (Figures 1E,H *p* < 0.05).

### PRR Silencing Decreased Myocardial Interstitial Fibrosis and Inflammation in Rats With Diabetic Cardiomyopathy

**FIGURE 2:**
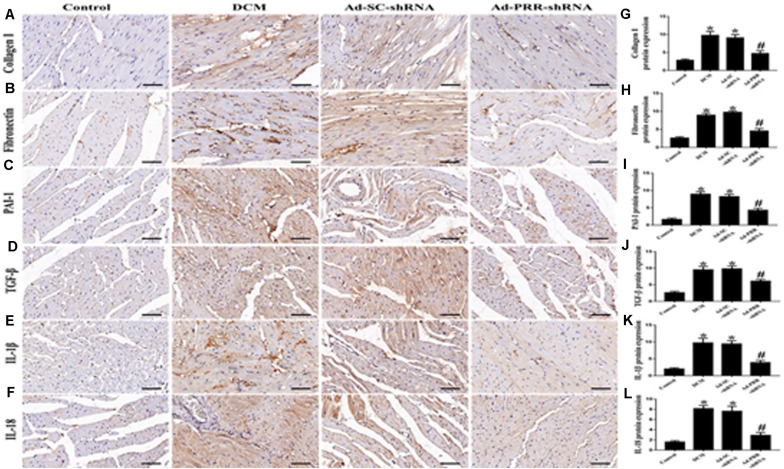
PRR silencing decreased myocardial interstitial fibrosis and inflammation in rats with diabetic cardiomyopathy (*n* = 5). **(A–F)** Representative immunohistochemical staining of Collagen I, Fibronectin, PAI-1, TGF-β, IL-1β and IL-18 in the 4 groups (scale bar = 20 μm). (**G–L)** Quantitative analysis of the Collagen I, Fibronectin, PAI-1, TGF-β, IL-1β, and IL-18 protein expression shown in panels **(A–F)**. *p < 0.05 versus the control group; ^#^*p* < 0.05 versus the Ad-SC-shRNA group.

The results of the immunohistochemical staining showed that the protein expression levels of Collagen I, Fibronectin, PAI-1 and TGF-β were significantly higher in the DCM group than in the control group (Figures 2A–D,G–J *p* < 0.05). Similarly, the protein expression levels of IL-1β and IL-18 were significantly upregulated in the DCM group compared with the control group (Figures 2E,F,K,L *p* < 0.05). The protein expression levels of Collagen I, Fibronectin, PAI-1, TGF-β, IL-1β and IL-18 were decreased in the Ad-PRR-shRNA group compared with the Ad-SC-shRNA group (Figures 2A–F *p* < 0.05).

### YAP Overexpression Increased Myocardial Interstitial Fibrosis and Inflammation in Rats With Diabetic Cardiomyopathy

**FIGURE 3:**
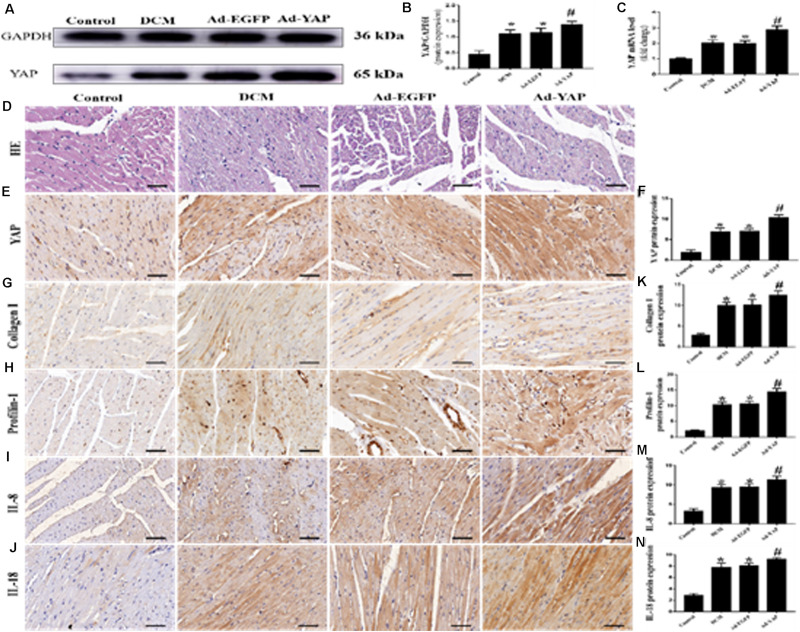
YAP overexpression increased myocardial interstitial fibrosis and inflammation in rats with DCM (*n* = 5). **(A)** western blot analysis of YAP protein expression in the 4 groups. **(B)** Quantitative analysis of the YAP protein expression shown in panel **(A)**. **(C)** YAP mRNA expression. **(D)** Representative HE staining of cardiomyocytes (scale bar = 20 μm) in the 4 groups. **(E)** Representative immunohistochemical staining of YAP in the 4 groups (scale bar = 20 μm). **(F)** Quantitative analysis of the YAP protein expression shown in panel **(E)**. **(G–J)** Representative immunohistochemical staining of Collagen I, Profilin-1, IL-8 and IL-18 in the 4 groups (scale bar = 20μm). **(K–N)** Quantitative analysis of the Collagen I, Profilin-1, IL-8 and IL-18 protein expression shown in panels **(G–J)**. *p < 0.05 versus the control group; ^#^*p* < 0.05 versus the Ad-EGFP group.

The western blot and immunohistochemical staining results showed that YAP protein expression was significantly increased in the Ad-YAP group compared with the DCM group, Ad-EGFP group and Control group (Figures 3A,B,E,F *p* < 0.05). YAP mRNA expression was statistically higher in the Ad-YAP group than that in the Ad-EGFP group, DCM group and Control group (Figure 3C *p* < 0.05).

The immunohistochemical staining results were consistent with the results of the western blots and PCR analysis. The protein expression levels of Collagen I, Profilin-1, IL-8 and IL-18 were higher in the Ad-YAP group than in the DCM group, Ad-EGFP group and Control group (Figures 3G–J,K–N *p* < 0.05). HE staining was applied in the 4 groups (Figure 3D). Rats in the Control group revealed regular collagen network structure. In contrast, rats in Ad-YAP group, Ad-EGFP group and DCM group revealed cardiac fibrosis, with destroyed and irregular collagen network.

### YAP Silencing Decreased Myocardial Interstitial Fibrosis and Inflammation in Rats With Diabetic Cardiomyopathy

**FIGURE 4:**
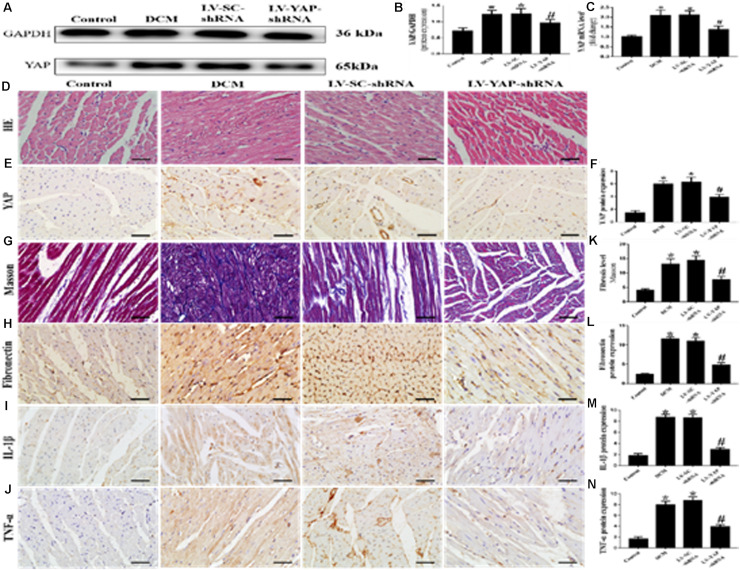
YAP silencing decreased myocardial interstitial fibrosis and inflammation in rats with DCM (*n* = 5). **(A)** Western blot analysis of YAP protein expression in the 4 groups. **(B)** Quantitative analysis of the YAP protein expression shown in panel **(A)**. **(C)** YAP mRNA expression. **(D)** Representative HE of the 4 groups (scale bar = 20 μm). **(E)** Representative immunohistochemical staining of YAP in the 4 groups (scale bar = 20 μm). **(F)** Quantitative analysis of the YAP protein expression shown in panel **(E)**. **(G)** Representative Masson’s trichrome staining of the myocardium in the 4 groups (scale bar = 20 μm). **(F–J)** Representative immunohistochemical staining of Fibronectin, IL-1β and TNF-α in the 4 groups (scale bar = 20 μm). **(K)** Quantitative analysis of the fibrosis level shown in panel **(G)**. **(L–N)** Quantitative analysis of the Fibronectin, IL-1β and TNF-α protein expression shown in panels **(H–J)**. *p < 0.05 versus the control group; ^#^*p* < 0.05 versus the LV-SC-shRNA group.

Western blotting, PCR analysis and immunohistochemical staining were used to measure the protein expression of YAP in vivo. The western blot and immunohistochemical staining results showed that YAP protein expression in the DCM group was significantly upregulated compared with the control group; however, this upregulation was significantly alleviated in the LV-YAP-shRNA group (Figures 4A,B,E,F *p* < 0.05). The immunohistochemical staining results showed YAP protein expression in the DCM group was significantly higher compared with the control group; however, this upregulation was significantly alleviated by YAP gene silencing in the LV-YAP-shRNA group. Similarly, the PCR results showed that YAP mRNA expression in the DCM group was also increased compared with the control group, and YAP gene silencing significantly reduced myocardial YAP mRNA expression in the LV-YAP-shRNA group (Figure 4C *p* < 0.05).

Masson staining results showed that the LV-YAP-shRNA group had lower collagen expression than the DCM and LV-SC-shRNA groups (Figures 4G,K *p* < 0.05). The immunohistochemical staining results showed that the Fibronectin, IL-1β and TNF-α were increased in the DCM group compared with the control group, but the protein expression levels of Fibronectin, IL-1β and TNF-α were reduced by YAP gene silencing in the LV-YAP-shRNA group (Figures 4H–J,L–N *p* < 0.05). Of course, HE staining was also applied in all 4 groups (Figure 4D).

### YAP Is Involved in Cardiac Function in Rats With Diabetic Cardiomyopathy

**FIGURE 5:**
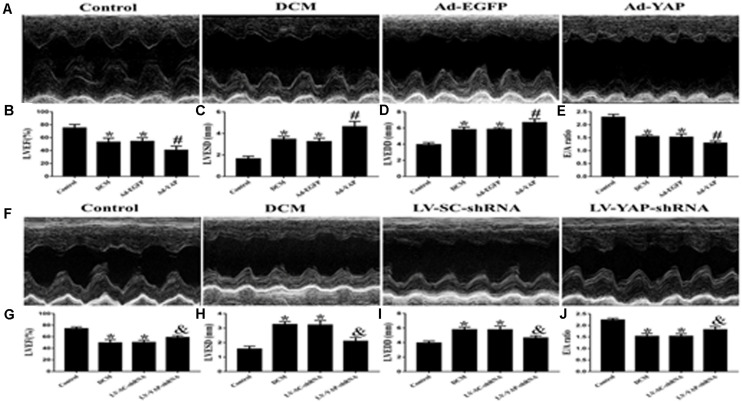
The effect of YAP overexpression or YAP silence on the cardiac function in rats with diabetic cardiomyopathy in two separate experiments. **(A)** M-mode echocardiograms in the 4 groups (Ad-YAP group, Ad-EGFP, DCM, and Control group) (*n* = 4). **(B)** (LVEF) in the 4 groups. **(C)** LVESD in the 4 groups. **(D)** LVEDD in the 4 groups. **(E)** E/A ratio respective levels in the 4 groups. **(F)** M-mode echocardiograms in the 4 groups (LV-YAP-shRNA group, LV-SC-shRNA group, DCM group and Control group). **(G)** LVEF in the 4 groups. **(H)** LVESD in the 4 groups was increased compared to that in the DCM group and the LV-SC-shRNA group. **(I)** LVEDD in the 4 groups. **(J)** E/A ratio respective levels in the 4 groups. *p < 0.05 versus the control group in the lower panels; ^#^*p* < 0.05 versus the Ad-EGFP group; ^&^*p* < 0.05 versus the LV-SC-shRNA group.

At the end of the experiment, echocardiography was used to evaluate the cardiac function of all of the rat groups (Figures 5A,F). Firstly, we observed the effect of YAP overexpression on cardiac function in DCM rats. The results showed that the LVEF was much lower in the DCM group and Ad-EGFP group than it was in the control group. However, overexpression of YAP worsened LVEF in the diabetic cardiomyopathy rats. The LVEF in the Ad-YAP group was decreased compared to that in the DCM group and Ad-EGFP group (Figure 5B). Left ventricular end-systolic diameters (LVESD) and left ventricular end-diastolic diameters (LVEDD) were increased in the DCM group compared with the control group, which were higher in the Ad-YAP group than in the DCM and Ad-EGFP groups (Figures 5C,D). The overexpression of YAP decreased the myocardial systolic function in DCM rats, as well as the diastolic function. The results showed that the E/A ratio was lower in the Ad-YAP group than it was in the DCM and Ad-EGFP groups (Figure 5E).

In contrast to YAP overexpression, YAP gene silencing improved cardiac function in DCM rats, including systolic function and diastolic function. The results showed that the LVEF in the LV-YAP-shRNA group was increased compared to that in the DCM group and the LV-SC-shRNA group (Figure 5G). The LVESD and LVEDD were lower in the LV-YAP-shRNA group than in the LV-SC-shRNA group (Figures 5H–I). Furthermore, the E/A ratio was improved in the LV-YAP-shRNA group more so than in the DCM group and LV-SC-shRNA group (Figure 5J).

### AMP-Activated Protein Kinase Agonist Increased *in vitro* AMPK Phosphorylation and Reduced the Fibrosis and Inflammation in Neonatal Cardiac Fibroblasts (CFs) With PRR Overexpression

**FIGURE 6:**
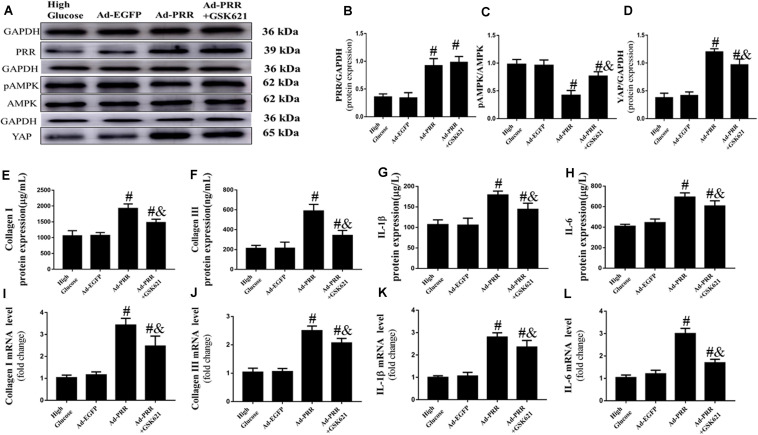
GSK621 increased AMPK phosphorylation and reduced the levels of inflammation and collagen-1 and collagen-3 in neonatal cardiac fibroblasts (CFs) with PRR overexpression (*n* = 4). **(A)** Western blot analysis of PRR, pAMPK, AMPK, and YAP protein expression in the 4 groups. **(B)** Quantitative analysis of the PRR protein expression shown in panel **(A)**. **(C)** Quantitative analysis of the pAMPK/AMPK shown in panel **(A)**. **(D)** Quantitative analysis of the YAP protein expression shown in panel **(A)**. **(E–H)** ELISA of Collagen I, Collagen III, IL-1β and IL-6 protein expression in the 4 groups. **(I–L)** The mRNA expression of Collagen I, Collagen III, IL-1β and IL-6. ^#^*p* < 0.05 versus the Ad-EGFP group; ^&^*p* < 0.05 versus the Ad-PRR group.

Firstly, we observed the changes in the expression of PRR and YAP and the level of AMPK phosphorylation. The western blot results showed that the PRR and YAP expression and the level of AMPK phosphorylation were similar in the High Glucose group and Ad-EGFP group, while the expression of PRR and YAP was increased and the level of AMPK phosphorylation was decreased in the Ad-PRR group compared to that in the Ad-EGFP group. The YAP expression was reduced and the level of AMPK phosphorylation was increased in the Ad-PRR+GSK621 group compared with the Ad-PRR group (Figures 6A–D).

The PRR overexpression aggravated the inflammation and fibrosis of CFs. The ELISA results showed that the expression of Collagen I, Collagen III, IL-1β and IL-6 were increased in the Ad-PRR group compared with the Ad-EGFP group; however, administration of GSK621 in the Ad-PRR+GSK621 group significantly decreased the levels of Collagen I, Collagen III, IL-1β and IL-6 compared with the Ad-PRR group (Figures 6E–H). The mRNA expression levels of Collagen I, Collagen III, IL-1β and IL-6 were coincident with the ELISA results (Figures 6I–L). These results demonstrated that GSK621 improved the effect of PRR overexpression in high-glucose stimulated CFs.

### Blood Glucose Levels

All of these data are from three independent experiment parts. The result showed that blood glucose levels were not statistically different among the groups in three separate experiments (Table 1).

**TABLE 1:** Blood glucose in three separate experiments in rats.

Groups	Glucose
Control	6.55 ± 0.69
DCM	24.30 ± 1.43
DCM+Ad-SC-shRNA	25.26 ± 1.81
DCM+Ad-PRR-shRNA	23.85 ± 1.68
Control	6.6 ± 0.78
DCM	25.40 ± 0.99
DCM+Ad-EGFP	24.53 ± 1.92
DCM+Ad-YAP	23.75 ± 1.25
Control	6.80 ± 0.18
DCM	27.96 ± 1.39
DCM+LV-SC-shRNA	28.02 ± 1.33
DCM+LV-YAP-shRNA	26.20 ± 1.51

## Discussion

Diabetic cardiomyopathy (DCM), as a complication of diabetes mellitus, is associated with structural defects in the heart muscle that impair ventricular performance and ultimately manifests as heart failure (Jia et al., 2018; Rabinovich-Nikitin et al., 2019). PRR and YAP are involved in diabetes and cardiovascular disease as has been previously reported by some studies. Previous reports suggested that the cardiac (pro)renin receptor is primarily expressed in the myocyte transverse tubules and its expression is increased in experimental diabetic cardiomyopathy (Connelly et al., 2011). Another study found that diastolic and systolic cardiac functions were significantly impaired in diabetic mice. Diabetes increases cardiac PRR expression and the nuclear translocation of PLZF (promyelocytic zinc finger protein), and activation of PLZF by PRR aggravates the inflammation and oxidative stress of the myocardium, which represents a novel mechanism in diabetic cardiomyopathy (Thomas et al., 2013). Of course, YAP also plays an important role in the cardiovascular system (Wang Y. et al., 2014; Leach et al., 2017).

In this study, our results showed that the expression levels of PRR and YAP were increased in the myocardium of DCM rats, suggesting that PRR and YAP are closely related to DCM. Therefore, we suspect that PRR and YAP may be involved in the pathological process of DCM.

Studies have shown that myocardial fibrosis and inflammation are important pathological manifestations of the pathogenesis of DCM. Myocardial inflammation has recently emerged as a pathophysiological process contributing to cardiac hypertrophy, fibrosis, and dysfunction in DCM (Chong et al., 2017; Frati et al., 2017). PRR is closely related to inflammation and the fibrosis of body organs. A previous study reported that PRR applied to human mesangial cells in culture increased the level of plasminogen activator inhibitor-1 (PAI-1) and then promoted fibrosis (Nguyen et al., 1996). In a mouse model of ischemia-reperfusion injury, transfection with PRR aggravated kidney dysfunction and worsened renal inflammation and fibrotic lesions (Li et al., 2017).

YAP is also involved in cardiac fibrosis and inflammation. One study reported that YAP is a mechanosensitive transcription factor that responds to changes in the actin filament organization and mediates pro-fibrotic signaling (Martin et al., 2016). YAP could aggravate cardiac fibrosis by regulating collagen expression in Ang II-stimulated cardiac fibrosis, and YAP inhibition could decrease the expression of collagen I stimulated by Ang II (Harikrishnan et al., 2019). YAP is also associated with inflammation. The inhibition of YAP can inhibit intestinal inflammation of the mouse colon in Crohn’s disease (Yu et al., 2018). Promoting the expression of YAP led to upregulation of the inflammatory cytokines TNF-α and IL-6, aggravating inflammatory lesions (Pan et al., 2019).

Our results suggested that overexpression of YAP increased the levels of inflammation and fibrosis. However, PRR and YAP gene silencing could reduce the expression of molecules involved in inflammation and fibrosis in DCM rats, and then reduce the levels of inflammation and fibrosis in the myocardium of DCM rats. These results may suggest that PRR and YAP are involved in the pathological process of diabetic cardiomyopathy by affecting the levels of inflammation and fibrosis. So PRR and YAP may provide new ideas for the treatment of DCM.

Our study found that YAP also has a certain influence on cardiac function in diabetic cardiomyopathy. Diabetic cardiomyopathy is characterized by diastolic dysfunction, which may precede the development of systolic dysfunction (Boudina and Abel, 2007). In the present study, our results showed that both cardiac systolic and diastolic function were decreased in DCM rats. Left ventricular end-systolic diameters (LVESDs) and LVEDDs were significantly increased, whereas LVEFs and E/A ratios were decreased in the in the Ad-YAP group in comparison with Ad-EGFP and the DCM groups. The result indicated that overexpression of YAP aggravated the cardiac dysfunction in diabetic cardiomyopathy. Compared with the DCM group, rats in the Ad-YAP group had worse cardiac function. These results suggest that YAP can aggravate the impairment of cardiac function in diabetic cardiomyopathy. Then, we used YAP gene silencing to observe the effect of inhibiting YAP on cardiac function in diabetic cardiomyopathy. Our results showed that cardiac systolic and diastolic function were improved in the Ad-YAP-shRNA group compared with the DCM group, indicating that YAP silencing improved cardiac function in the DCM rat model.

Recent studies have also shown that YAP is closely related to cardiac function in cardiovascular disease. There was a report that showed that YAP is upregulated in human ischemic heart failure. What’s more, inhibition of the Hippo pathway, which can reverse ischemic heart failure, may have therapeutic benefits for patients with ischemic heart failure (Leach et al., 2017). Therefore, the inhibition of YAP expression in diabetic cardiomyopathy may provide a new direction for the improvement of cardiac function in patients with diabetic cardiomyopathy.

The involvement of PRR and YAP in the pathological process of diabetic cardiomyopathy may be related to AMP-activated protein kinase (AMPK). AMPK is closely associated with the pathologic progression of diabetic cardiomyopathy. As reported in a previous study, phosphorylation of AMPK (pAMPK) was reduced in diabetic cardiomyopathy mice and high glucose-treated cardiomyocytes, and inflammation and pyroptosis markers such as caspase-1 and IL-1β were significantly increased. Metformin increased pAMPK and decreased caspase-1 and IL-1β expression, thereby improving the symptoms of diabetic cardiomyopathy (Yang et al., 2019). A previous study reported that PRR could regulate the phosphorylation of AMPK. The phosphorylation of AMPK is decreased and the expression of PRR is increased in diabetic nephropathy. The phosphorylation of AMPK can be increased by inhibiting the expression of PRR (Akhtar and Siragy, 2019). In the present study, our results similarly showed that PRR expression was increased and pAMPK expression was decreased in diabetic cardiomyopathy rats compared with the control group. PRR gene silencing increased the phosphorylation of AMPK in rats with diabetic cardiomyopathy. In addition, PRR gene overexpression decreased the phosphorylation of AMPK in high-glucose stimulated neonatal cardiac fibroblasts. Our result indicated that PRR modulates the phosphorylation of AMPK in DCM.

Meanwhile, the expression of YAP is also closely related to AMPK. A previous study showed that an increase of pAMPK can inhibit the expression of YAP, while inhibiting the expression of pAMPK causes the expression of YAP to increase (Jiang et al., 2016). Furthermore, it has also been reported that AMPK activation could inhibit YAP activity in cellular energy stress, established the molecular mechanism and functional significance of AMPK in linking the cellular energy status to YAP (Mo et al., 2015). Our experimental results also confirmed this. In the present study, our results showed that the expression level of PRR increased and the expression level of pAMPK decreased in diabetic cardiomyopathy rats, leading to an increase in the expression level of YAP, which led to an increase of fibrosis and inflammation in the myocardium of diabetic cardiomyopathy rats. PRR gene silencing could improve this situation.

To further verify that PRR increases YAP expression by reducing pAMPK expression and thus participates in the pathological process of diabetic cardiomyopathy, we designed a series of in vitro cell experiments. Our results showed that in high glucose-stimulated rat primary cardiac fibroblasts (CFs), the overexpression of PRR reduced pAMPK expression and increased YAP expression, thereby aggravating the inflammation and fibrosis in CFs, while GSK621 (an AMPK agonist) ameliorated these effects. In the Ad-PRR+GSK621 group, the expression level of PRR remained unchanged, the expression of pAMPK increased, the expression of YAP decreased, and the levels of inflammation and collagen-1 and collagen-3 of CFs improved compared with the Ad-PRR group. From above study, we speculated that PRR influences the pathological process of diabetic cardiomyopathy through the PRR-AMPK-YAP pathway. In most pathological conditions, PRR mainly functions independently on RAS, such as MAPK and MAPk signaling or Wnt/β-catenin signaling et al.

In this investigation, we focused on only one pathway (PRR-AMPK-YAP pathway), and the effect of PRR on other pathways (PRR- RAS pathway) should also be explored in the future.

## Data Availability Statement

The original contributions presented in the study are included in the article/supplementary material, further inquiries can be directed to the corresponding author/s.

## Ethics Statement

The animal study was reviewed and approved by Animal Care and Use Committee of Shandong Provincial Hospital, Shandong University.

## Author Contributions

JX, XD, and SL analyzed the data. JX, XD, SL, BD, and QS wrote the manuscript. JX, XD, SL, FJ, JC, SY, and BD designed the research. All authors contributed to the article and approved the submitted version.

## Conflict of Interest

The authors declare that the research was conducted in the absence of any commercial or financial relationships that could be construed as a potential conflict of interest.
