# Supplementary material for: Effects of (Pro)renin Receptor on Diabetic Cardiomyopathy Pathological Processes in Rats via the PRR-AMPK-YAP Pathway
Source: Front Physiol. 2021 May 27;12:657378. doi: 10.3389/fphys.2021.657378 (PMC8191636; doi:10.3389/fphys.2021.657378)

Figure 1

Original images (un-cropped images)

GAPDH

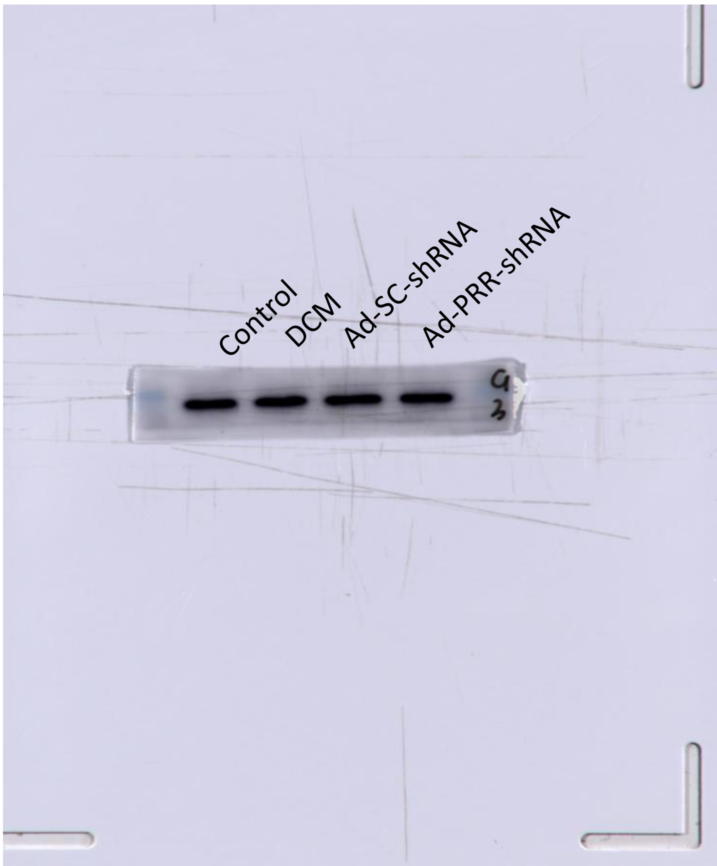

PRR

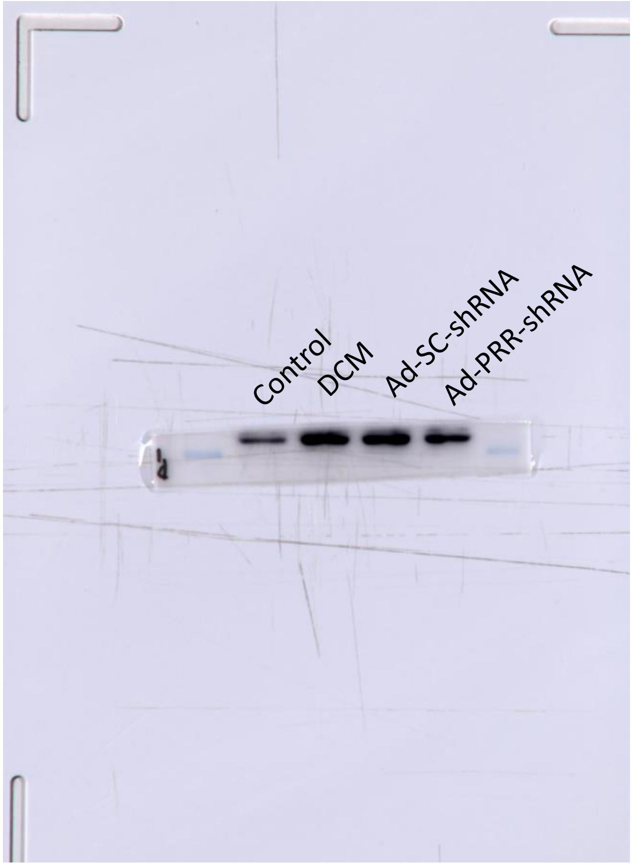

pAMPK

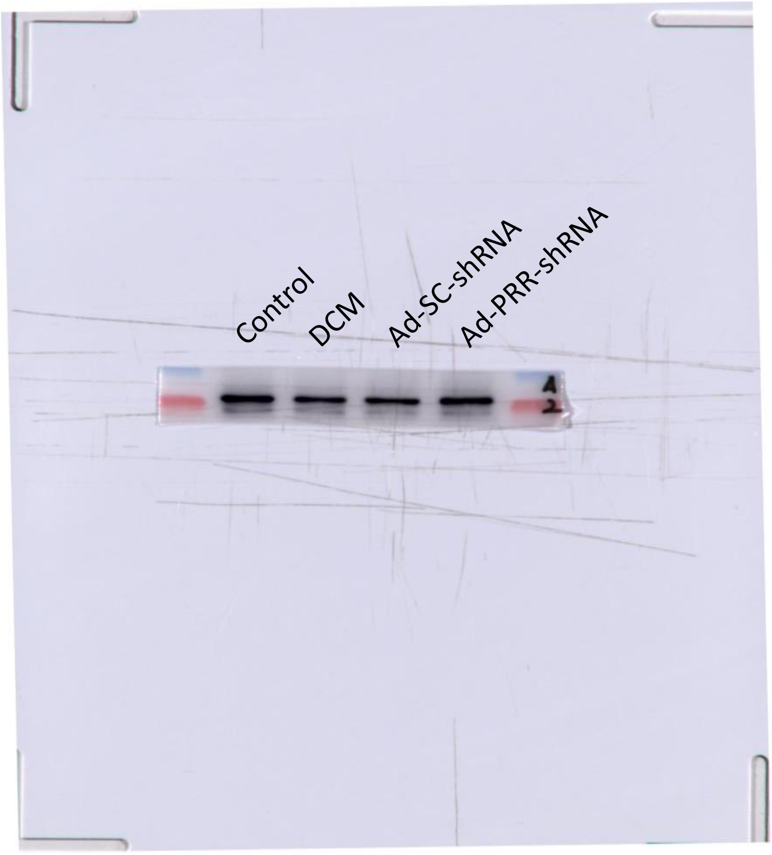

AMPK

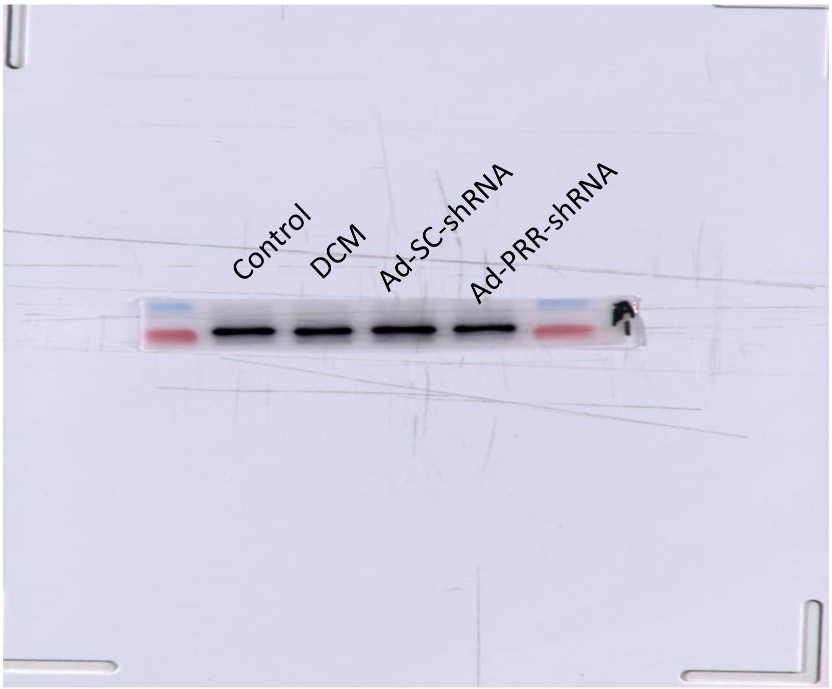

YAP

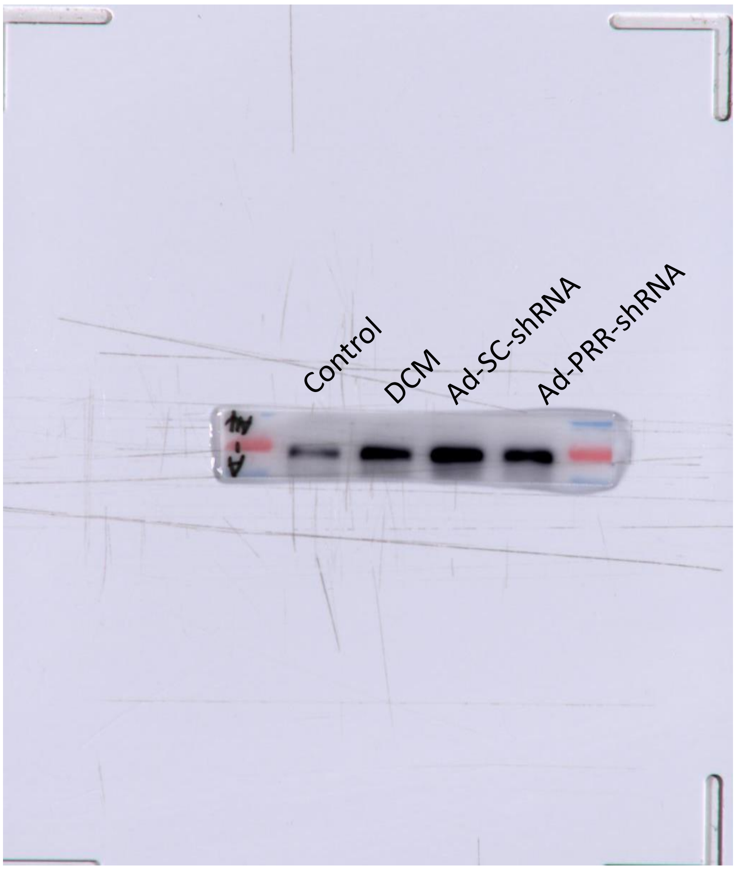

Figure 3

GAPDH

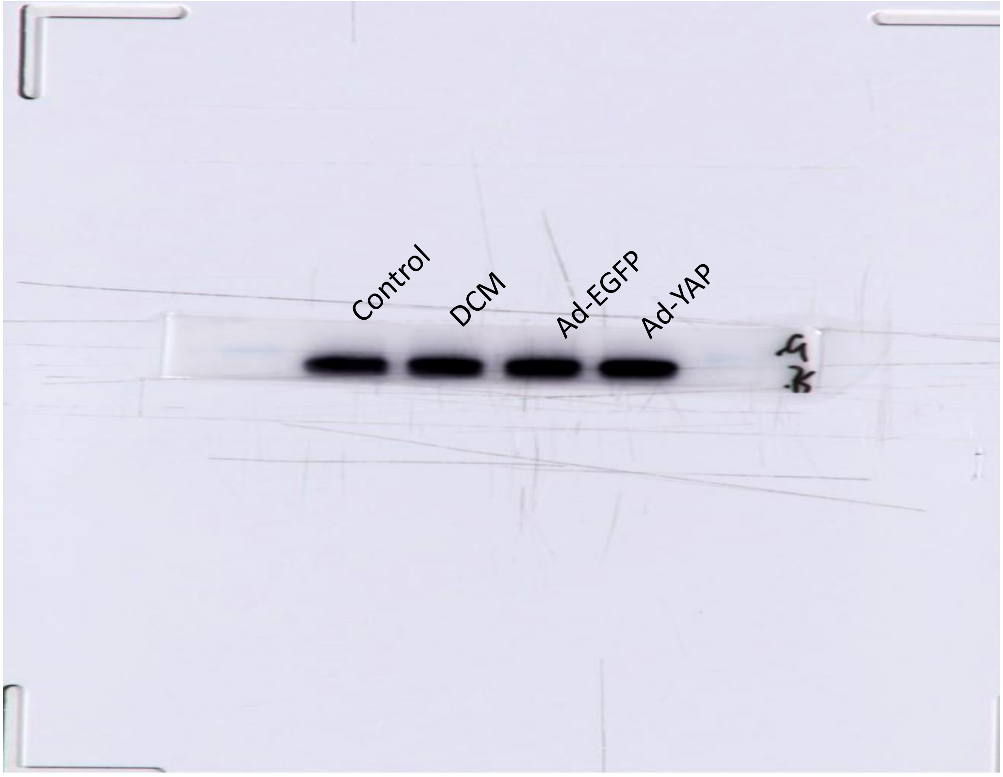

YAP

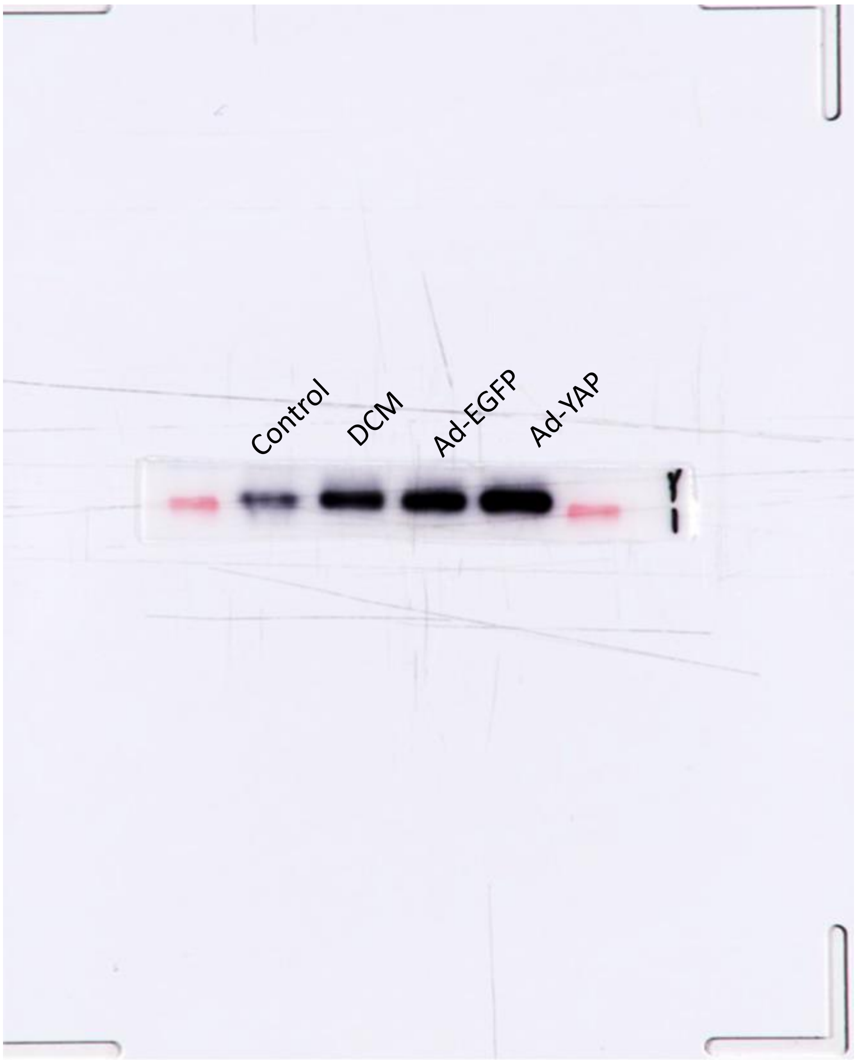

Figure 4

GAPDH

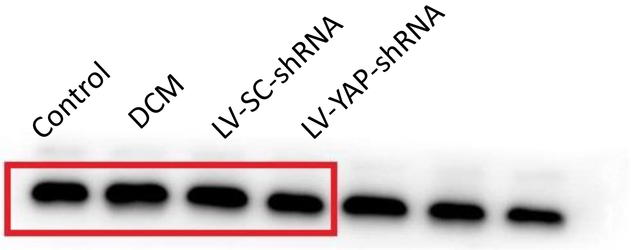

YAP

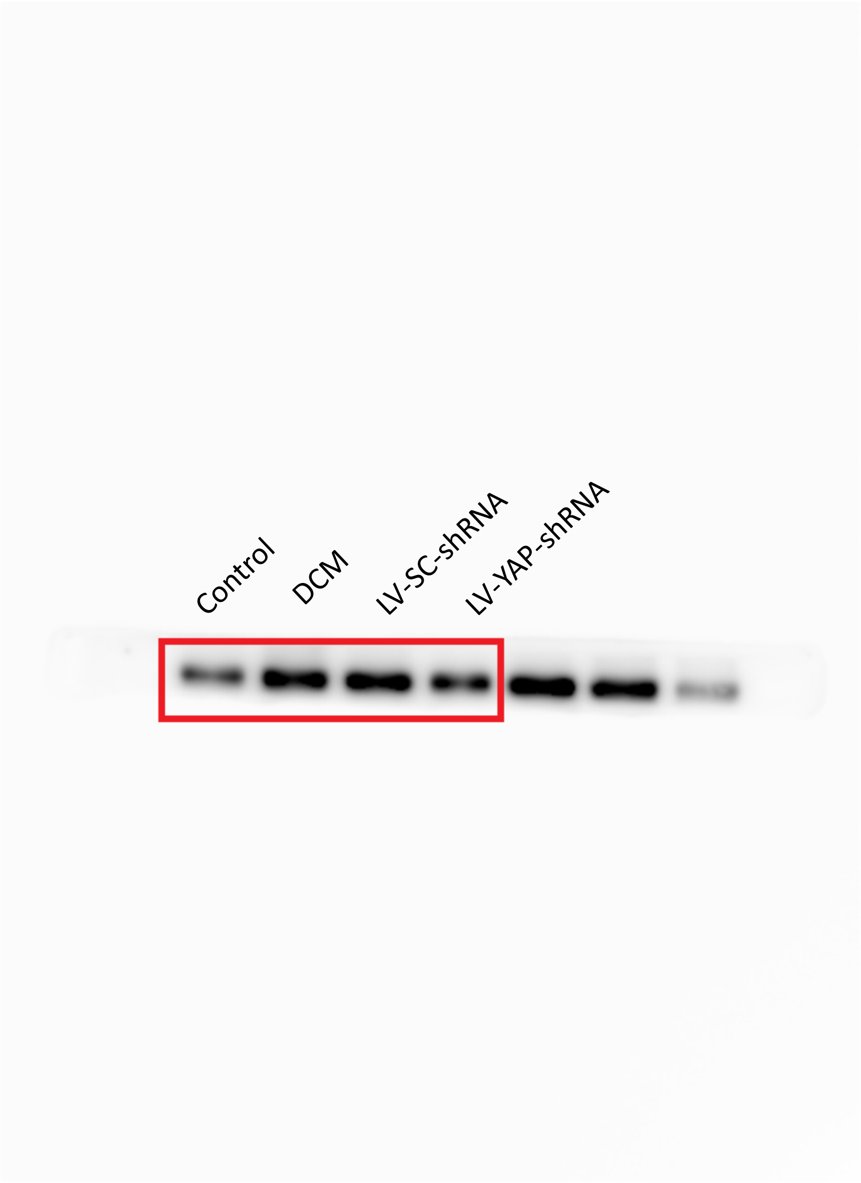

Figure 6

GAPDH

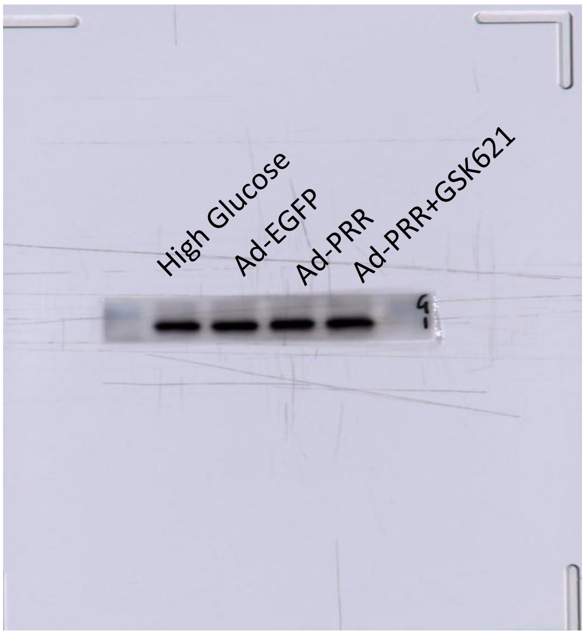

PRR

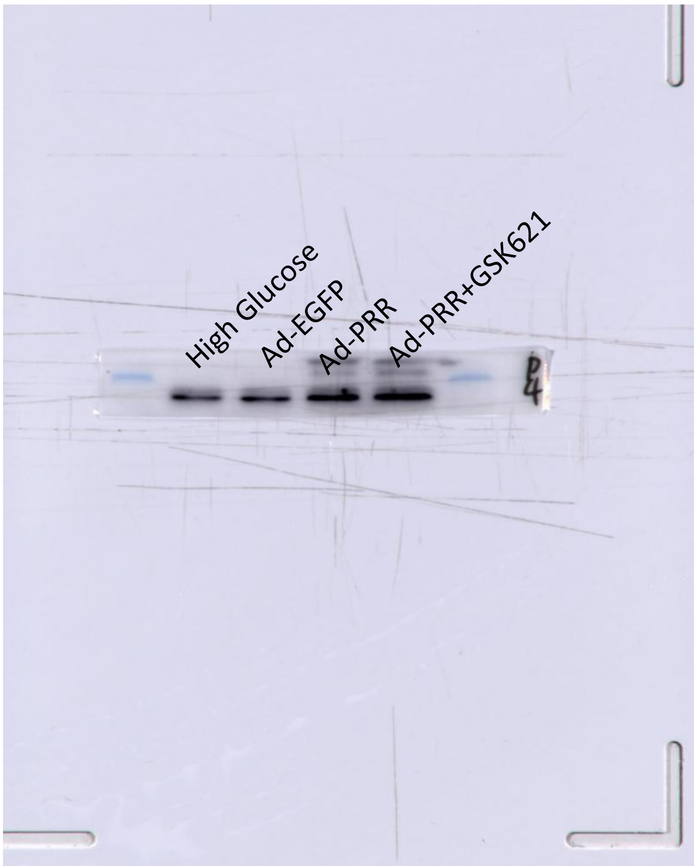

GAPDH

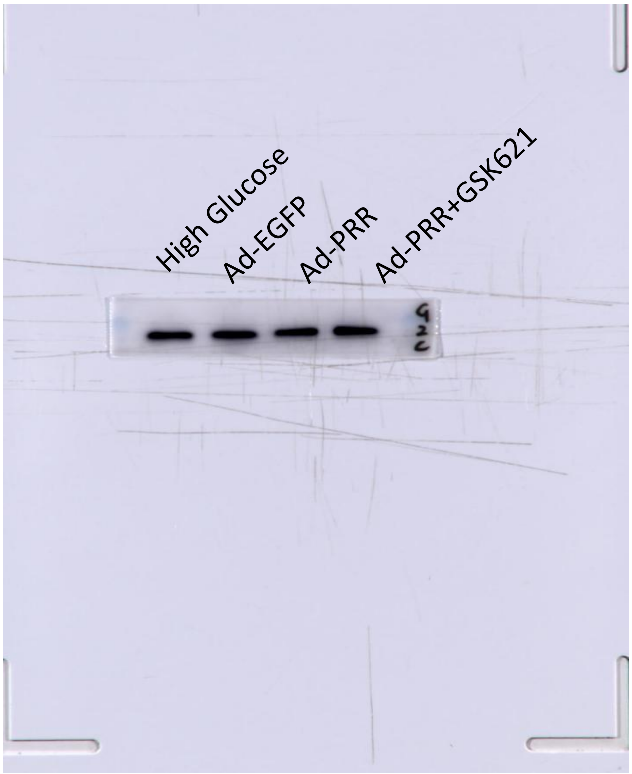

PAMPK

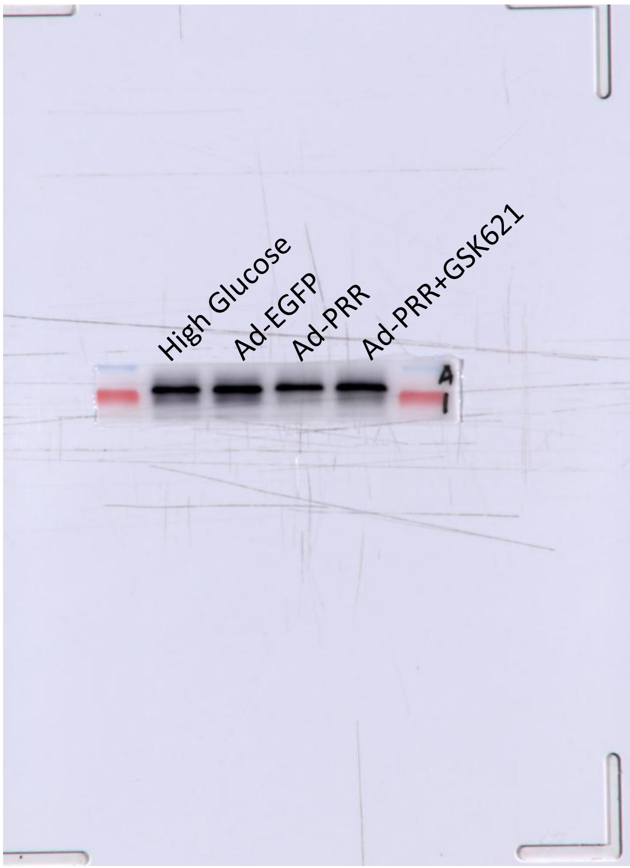

AMPK

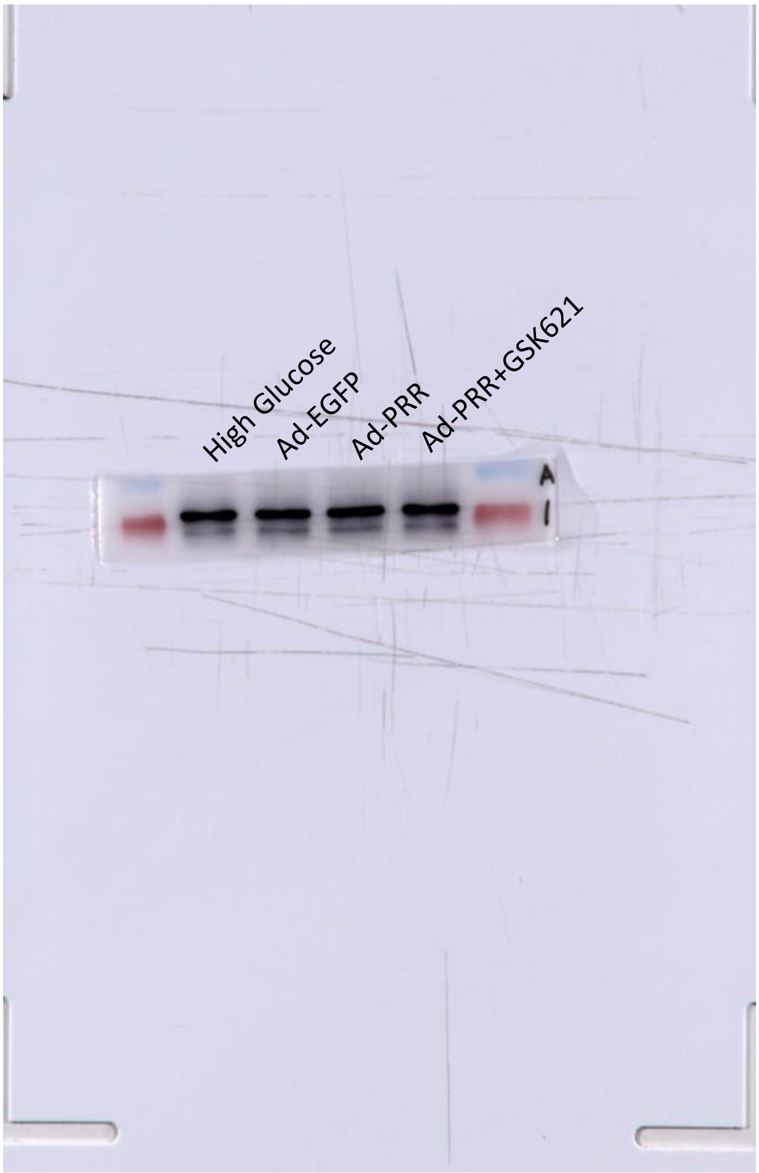

GAPDH

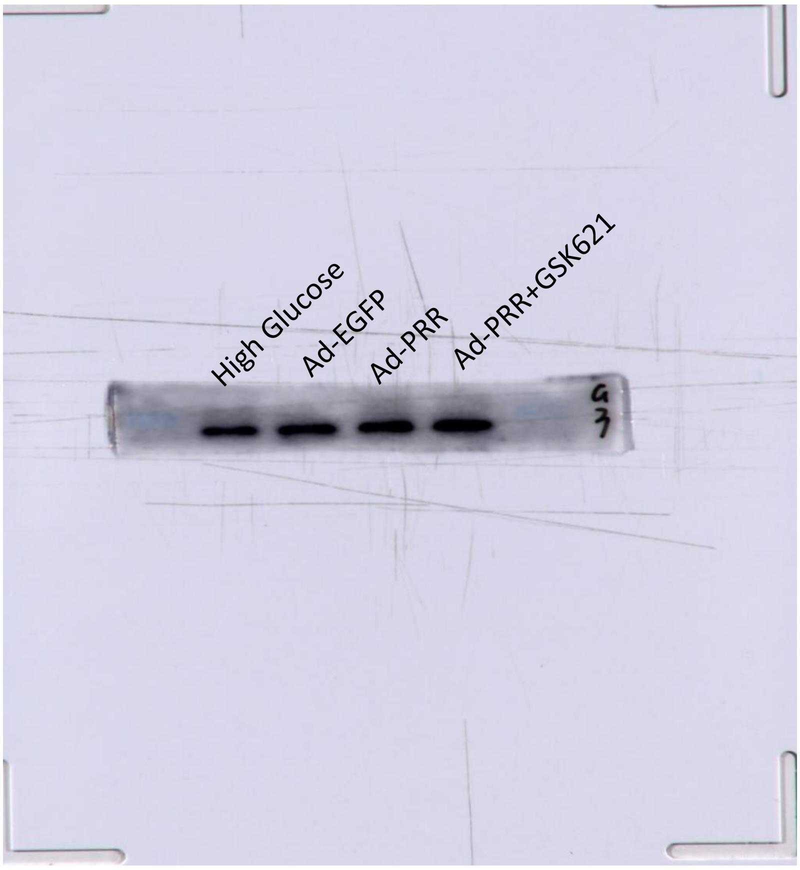

YAP

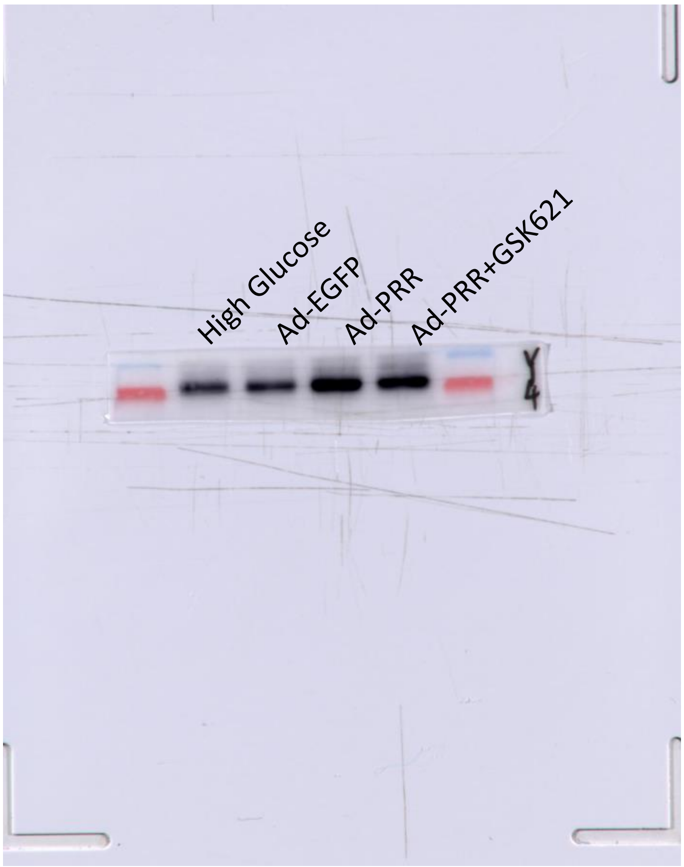

Supplement: Supplementary file 1 [file Data_Sheet_1.pdf]
