# Supplementary material for: Effects of (Pro)renin Receptor on Diabetic Cardiomyopathy Pathological Processes in Rats via the PRR-AMPK-YAP Pathway
Source: Front Physiol. 2021 May 27;12:657378. doi: 10.3389/fphys.2021.657378 (PMC8191636; doi:10.3389/fphys.2021.657378)

Original images (un-cropped images) for Immunohistochemical staining , HE staining and cardiac function

Figure1

PRR

Control

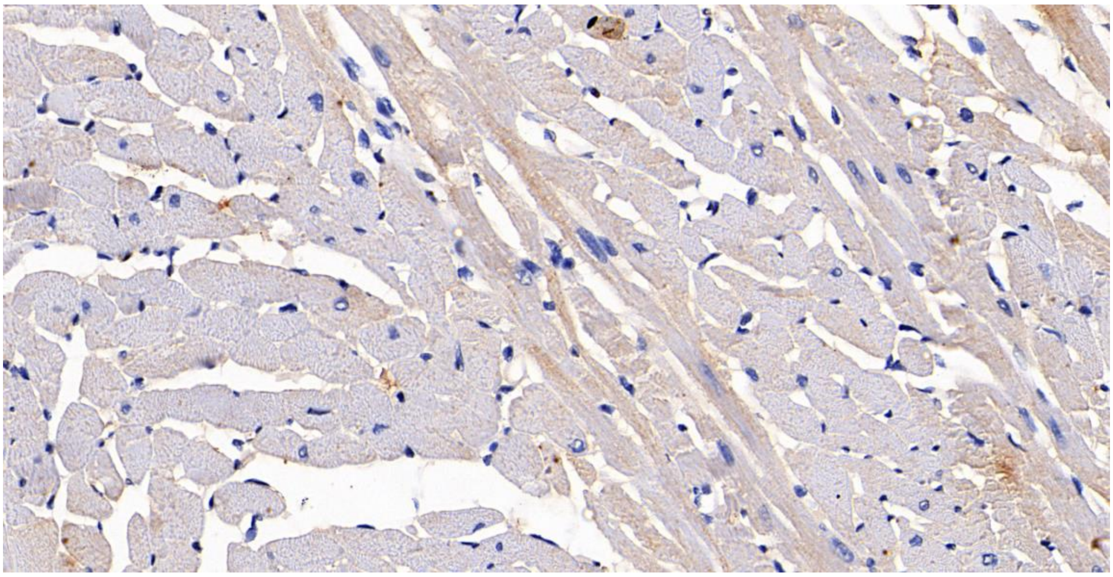

DCM

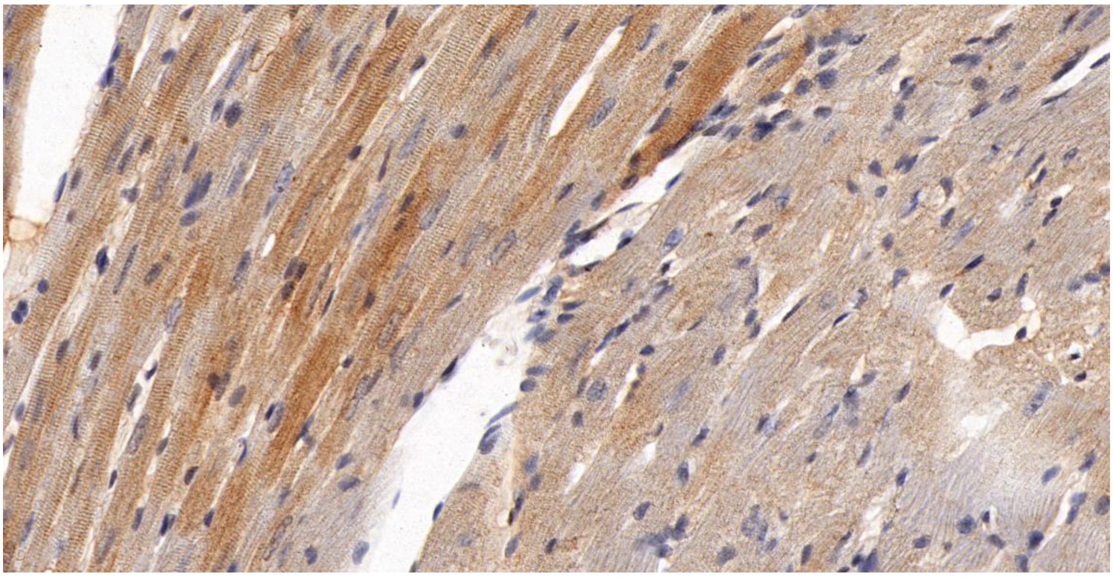

Ad-SC-shRNA

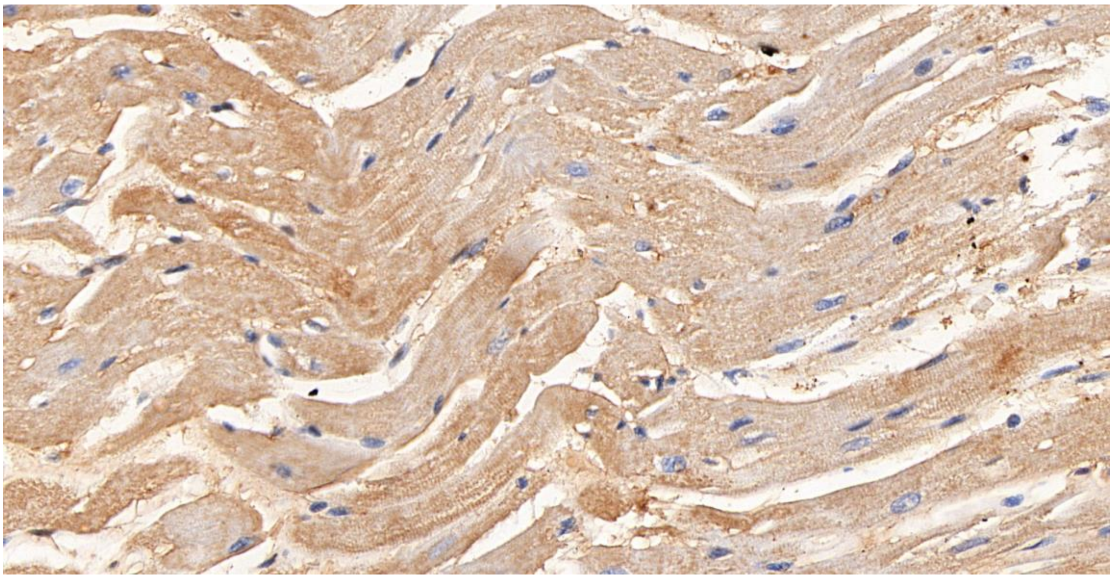

Ad-PRR-shRNA

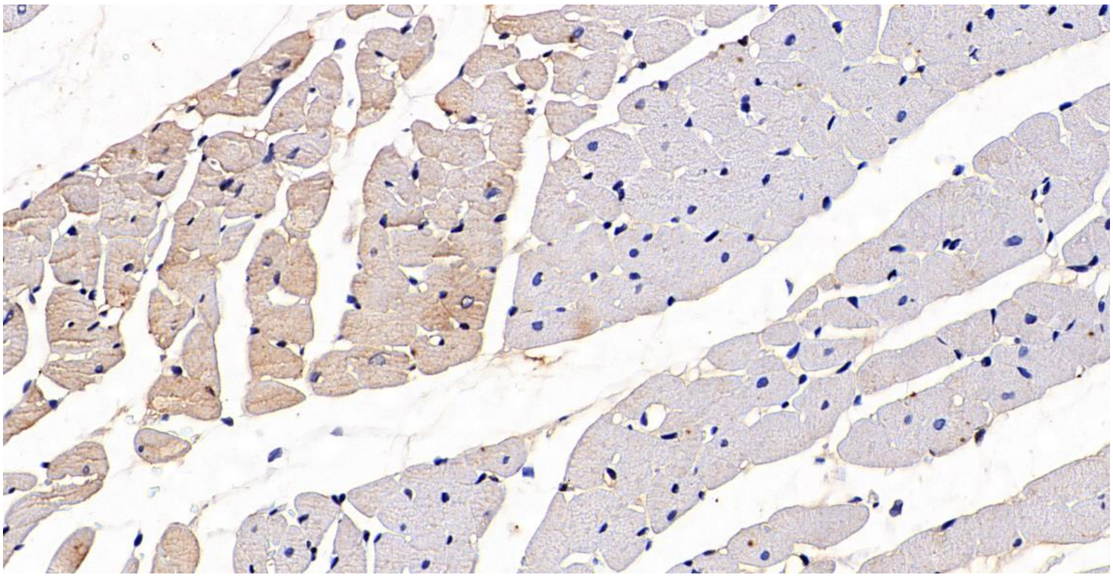

YAP

Control

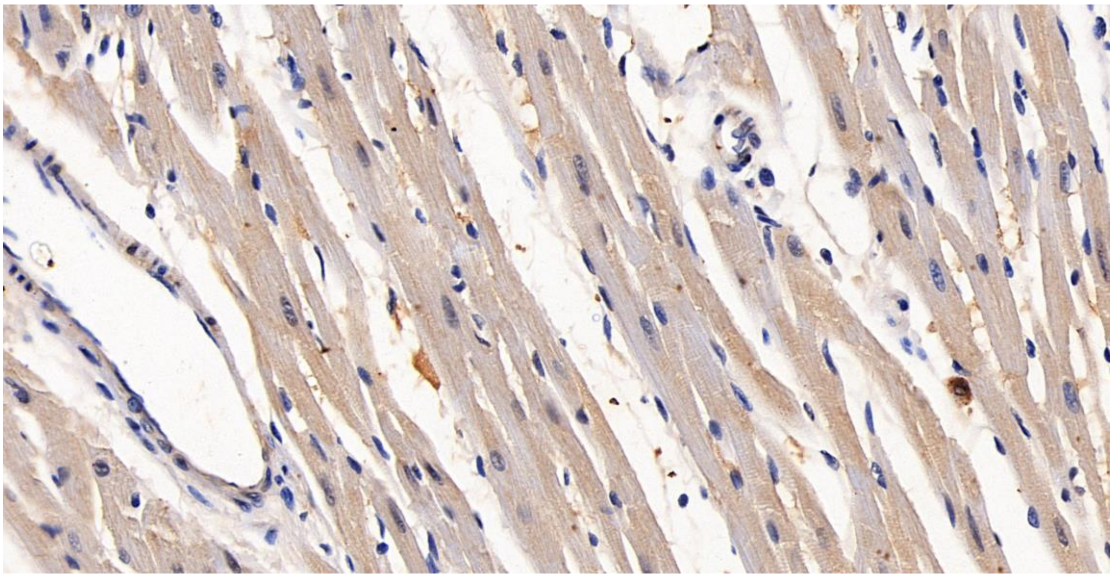

DCM:

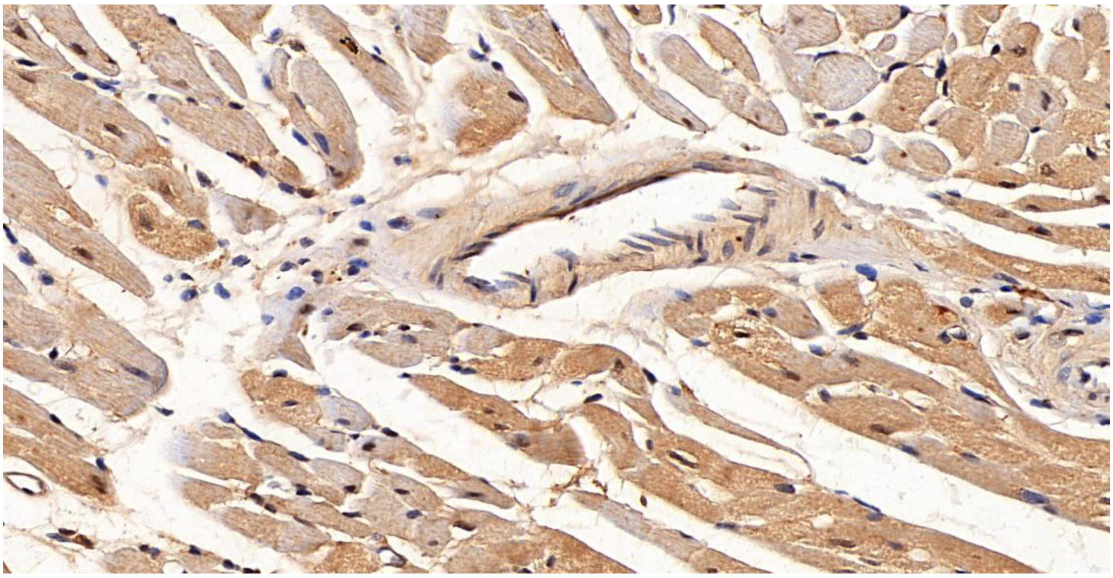

Ad-SC-shRNA:

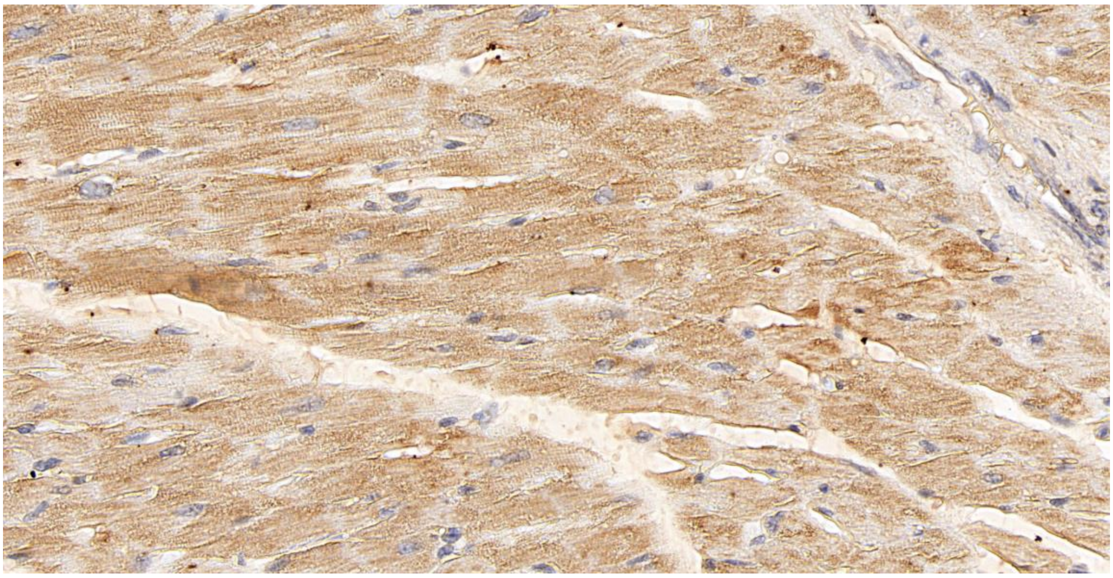

Ad-PRR-shRNA

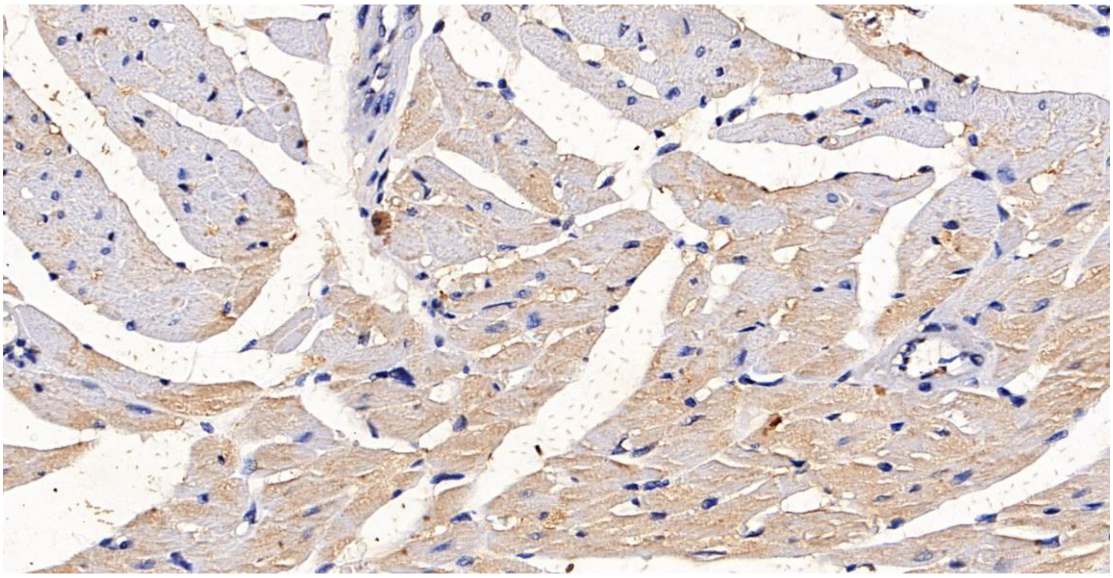

Figure2

Collagen I

Control

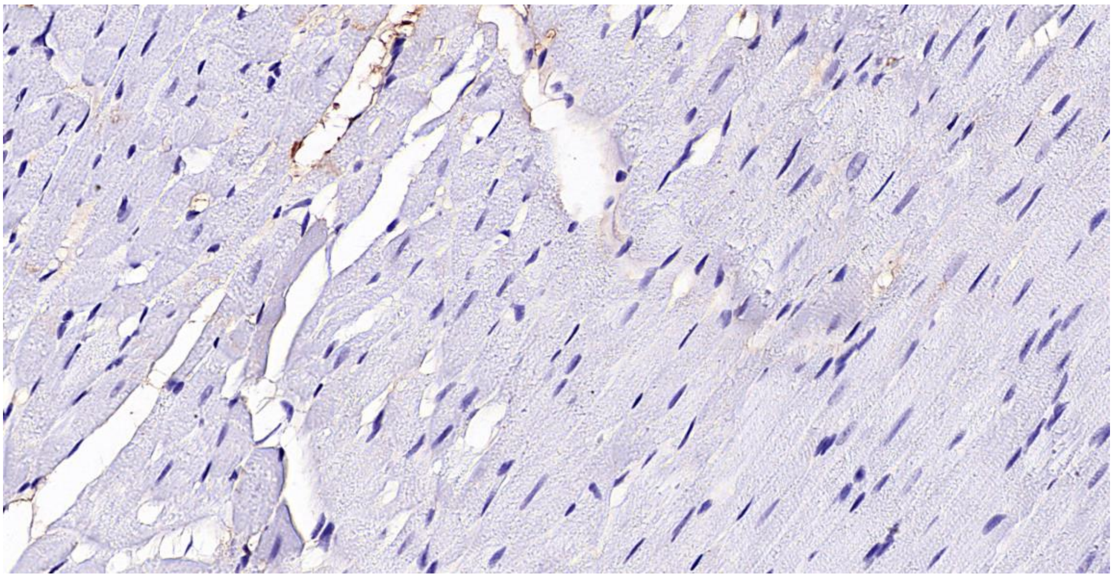

DCM:

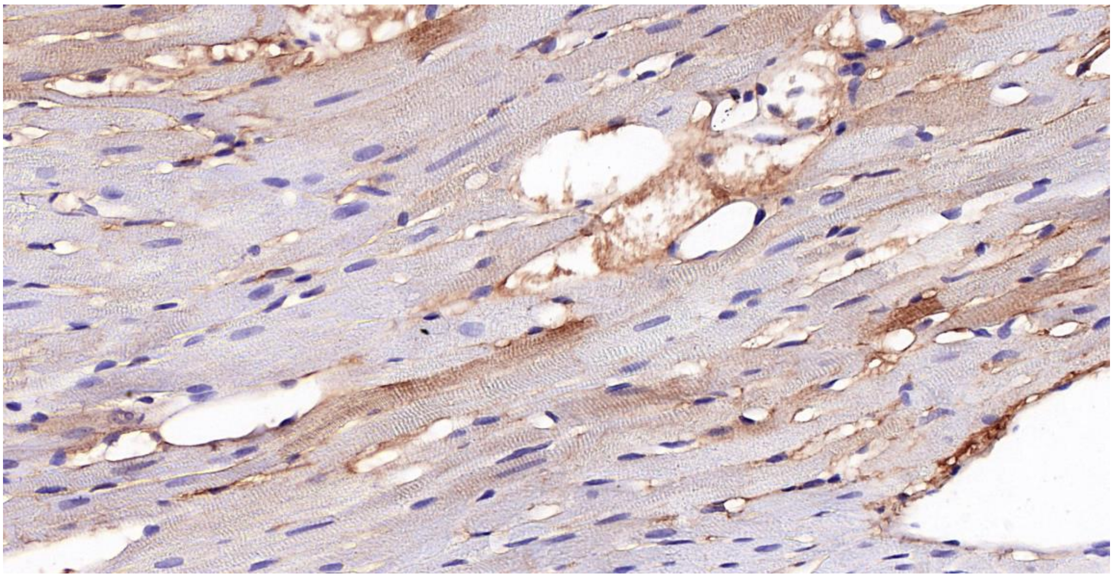

Ad-SC-shRNA

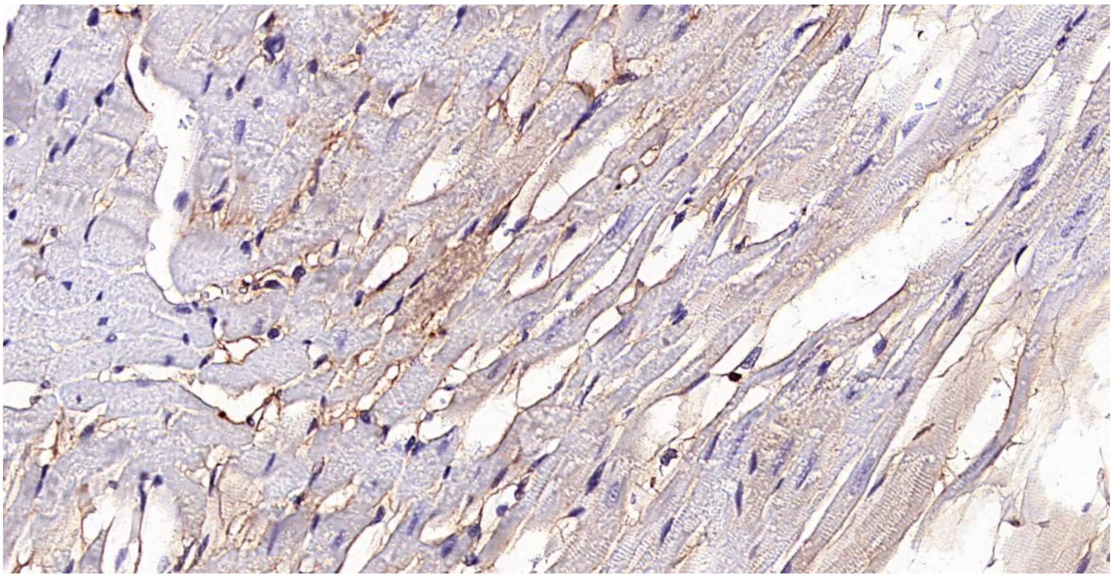

Ad-PRR-shRNA:

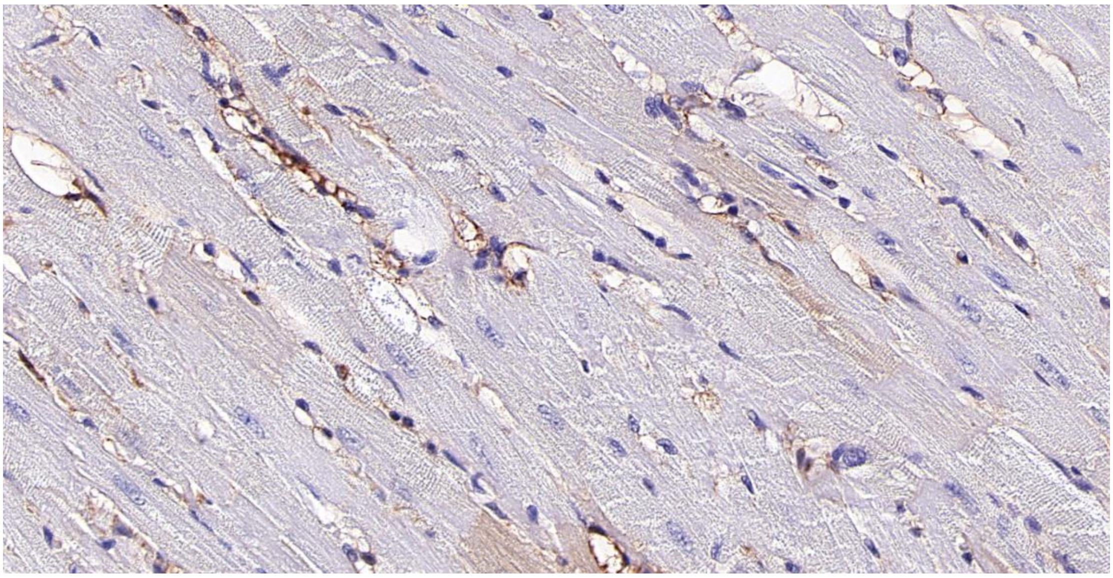

Fibronectin

Control

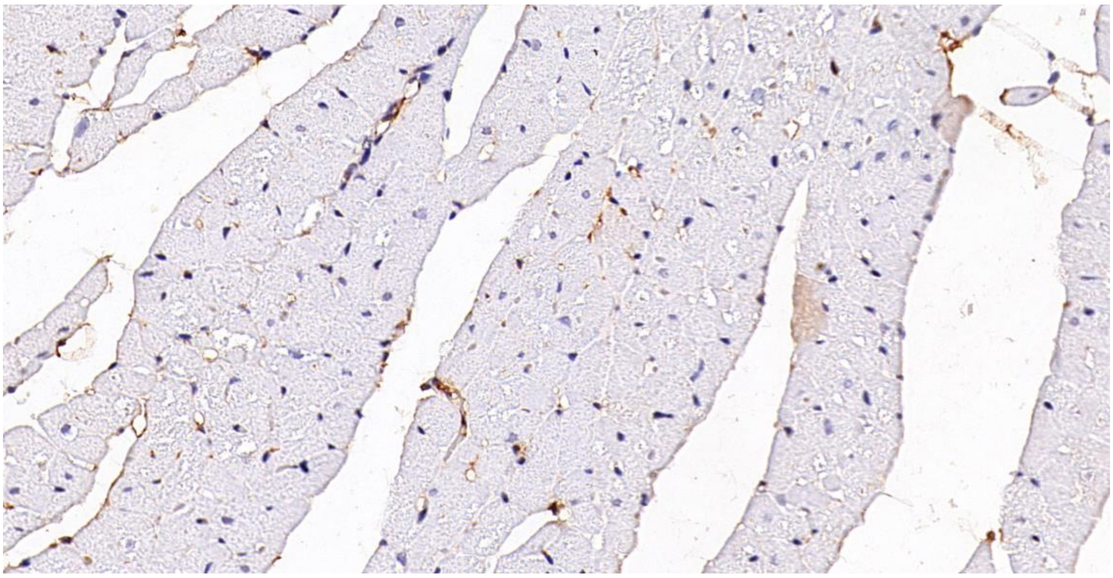

DCM

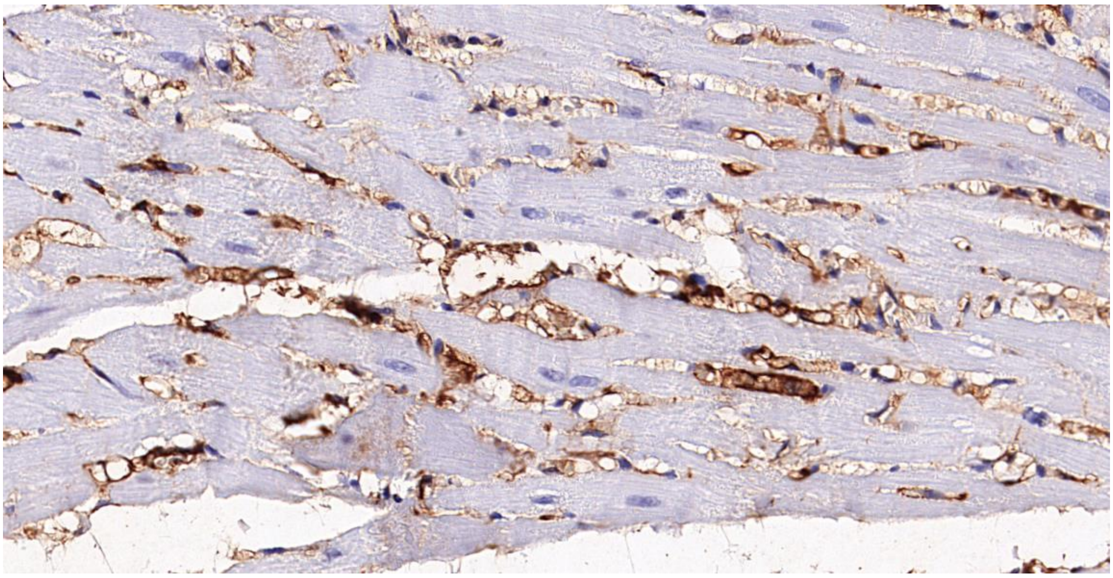

Ad-SC-shRNA

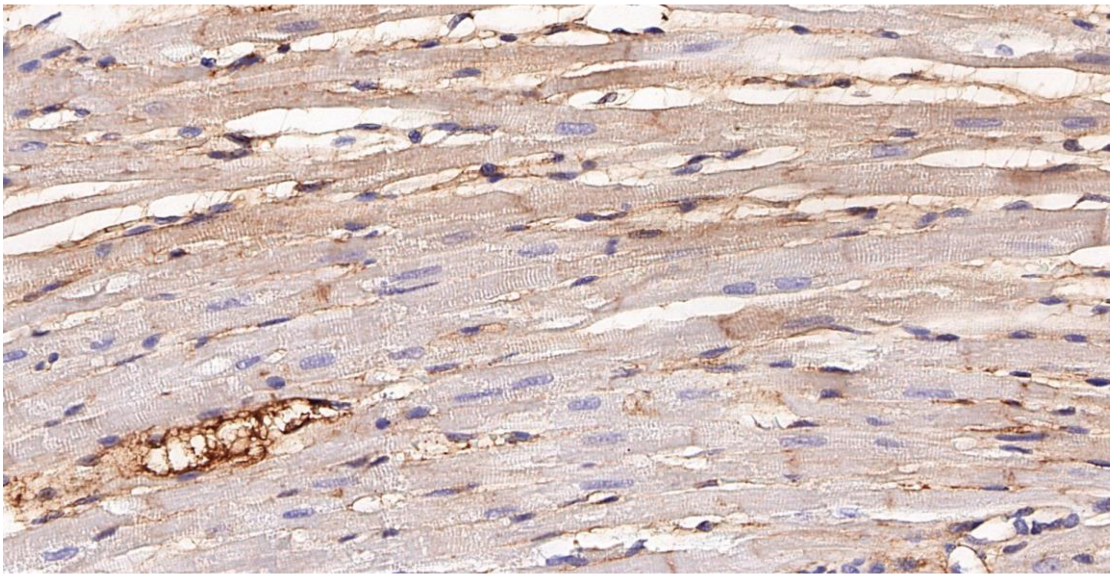

Ad-PRR-shRNA

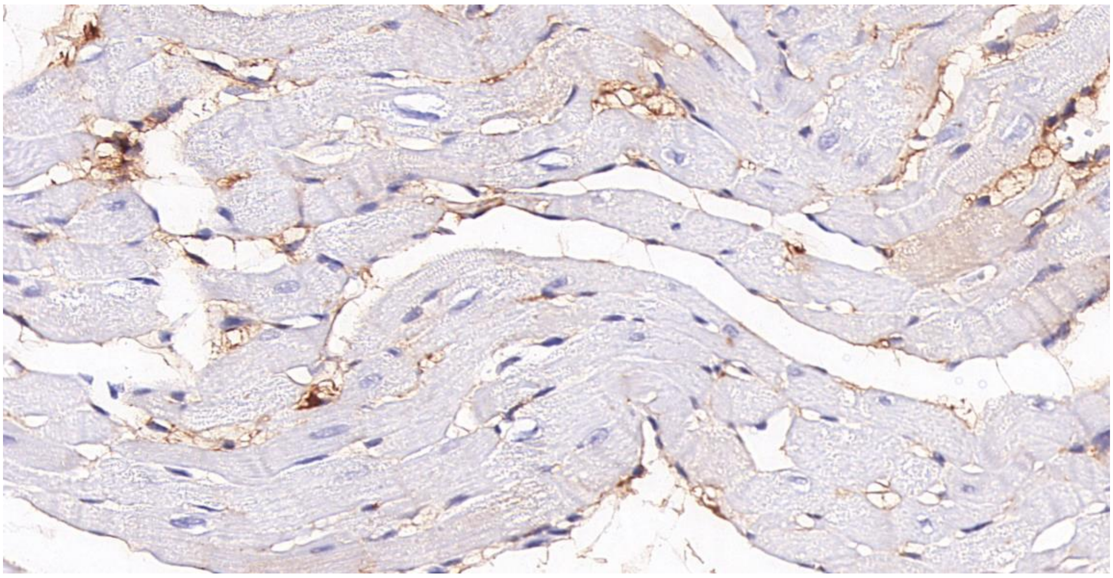

PAI-1

Control:

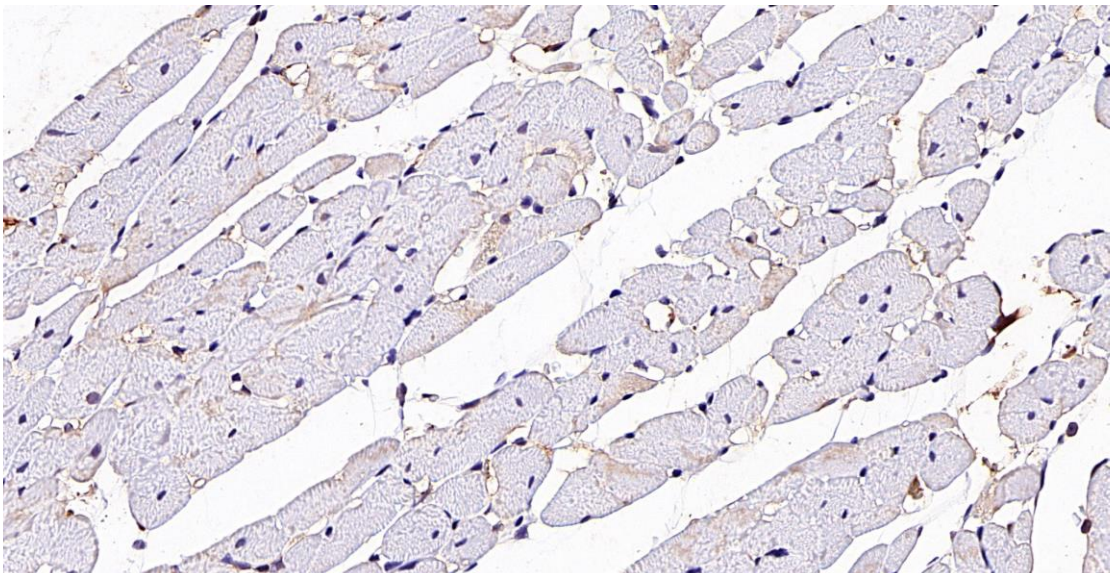

DCM

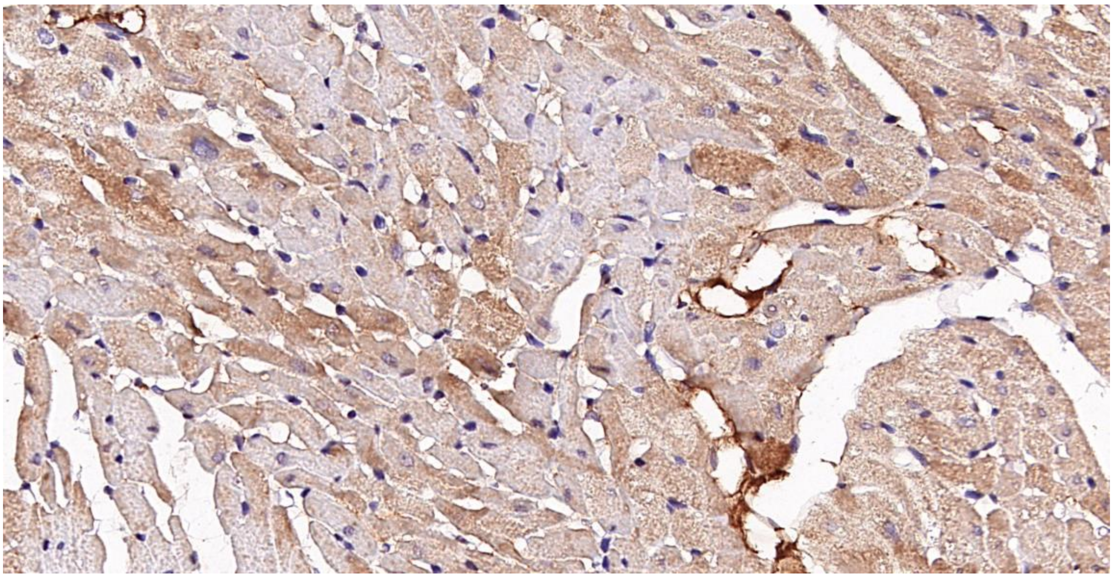

Ad-SC-shRNA

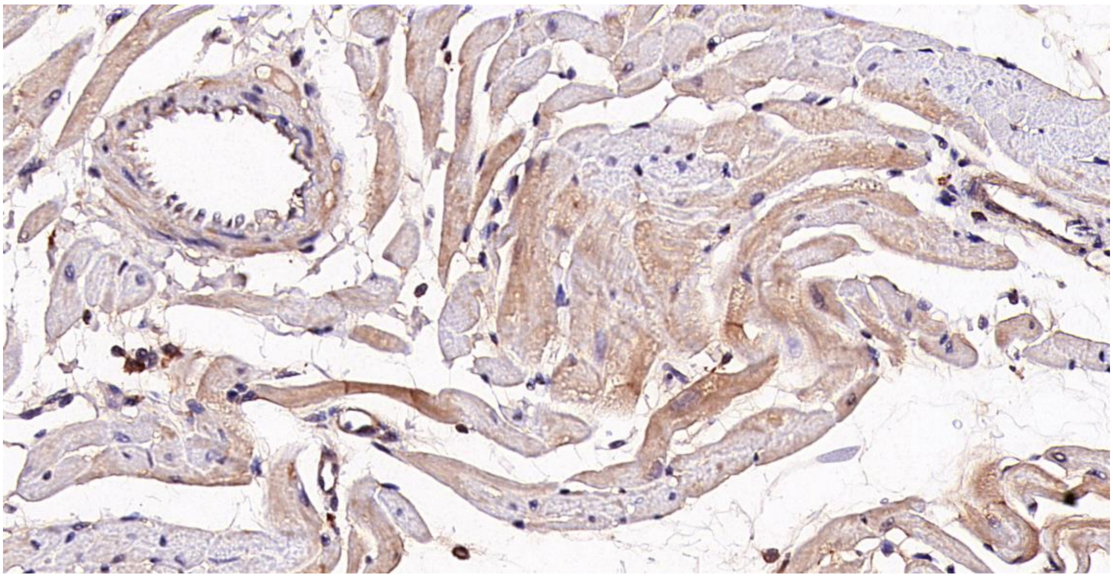

Ad-PRR-shRNA

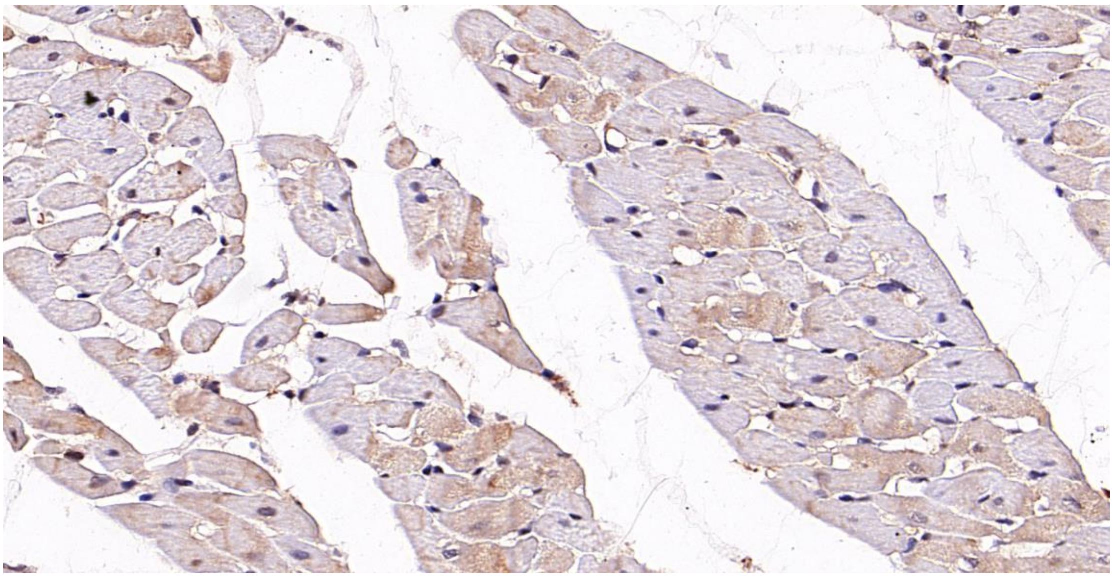

TGF-β

Control

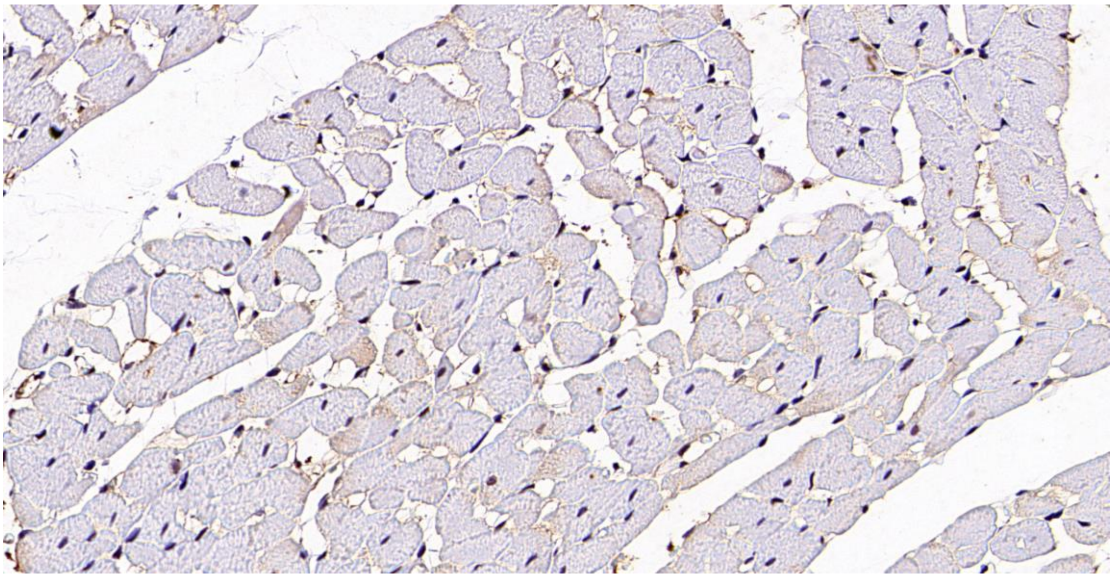

DCM

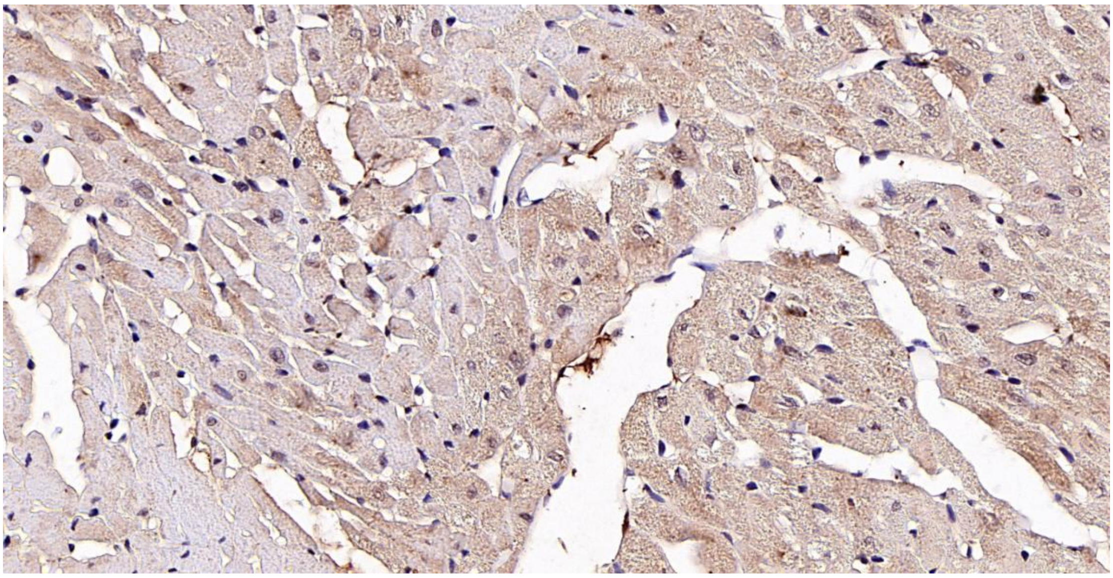

Ad-SC-shRNA

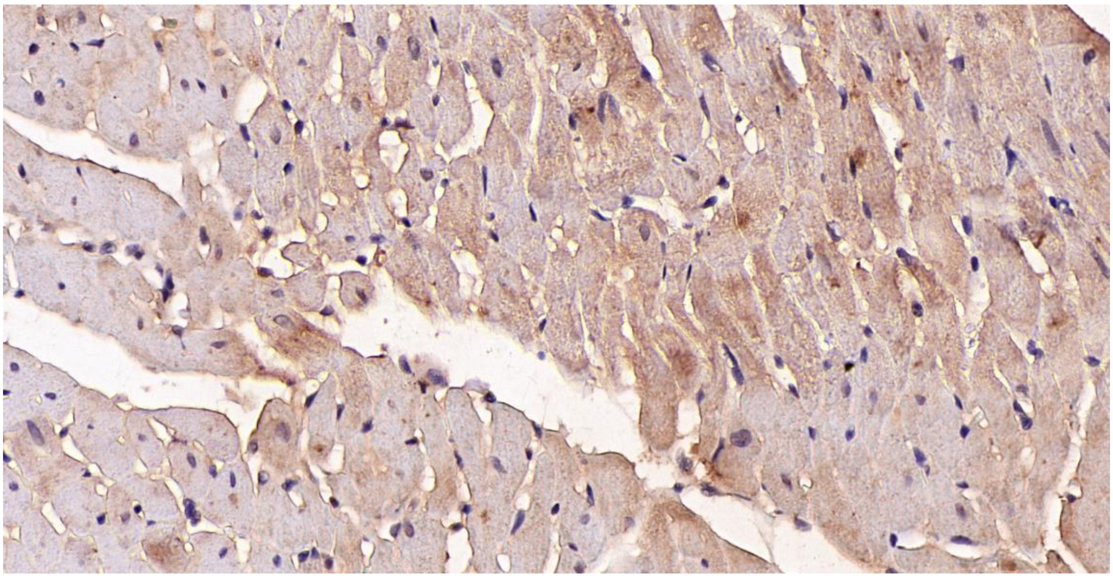

Ad-PRR-shRNA

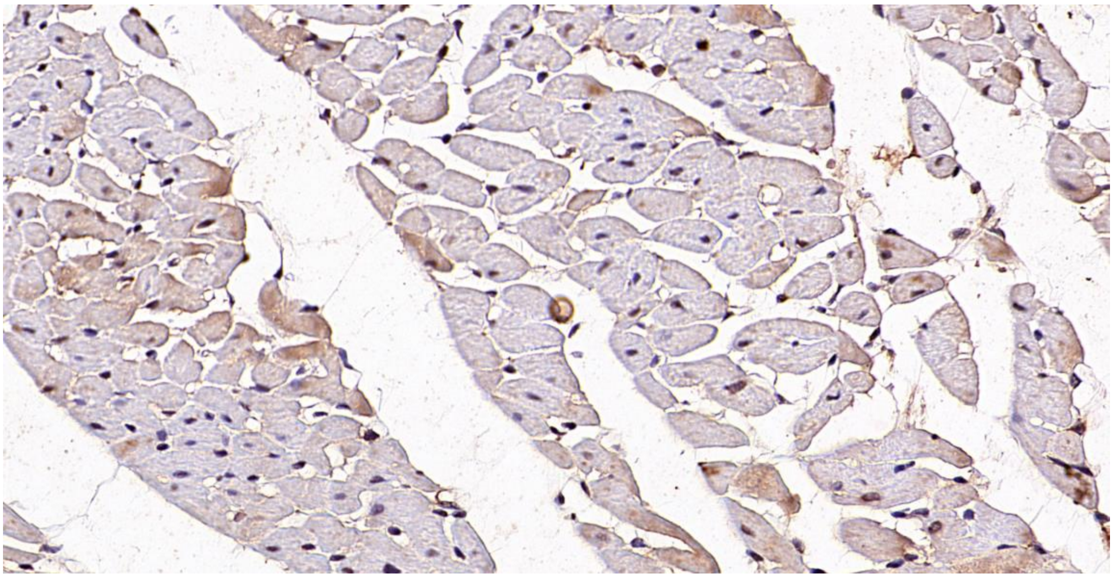

IL-1β

Control

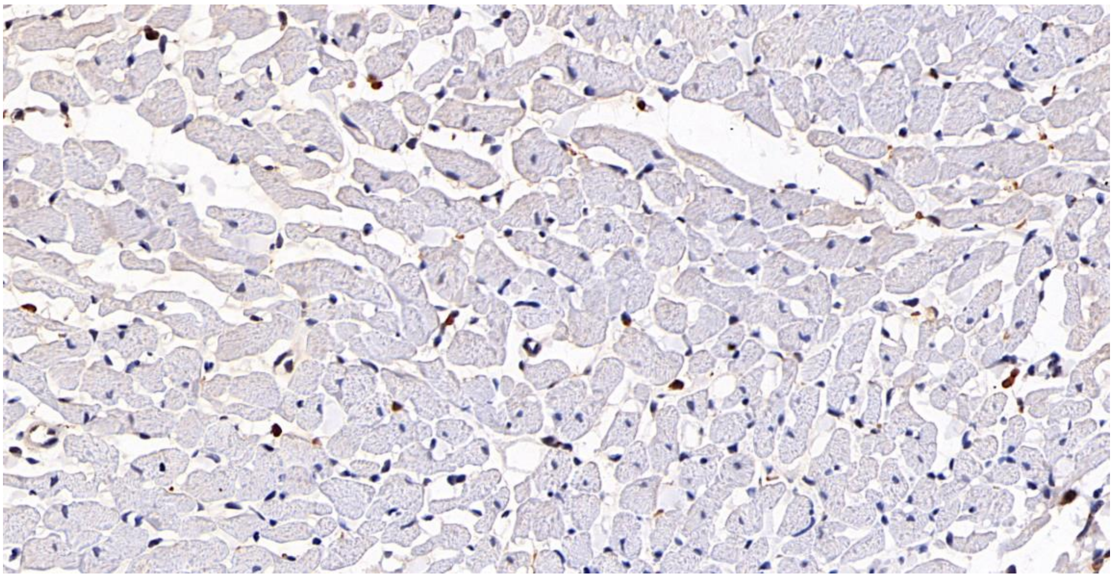

DCM

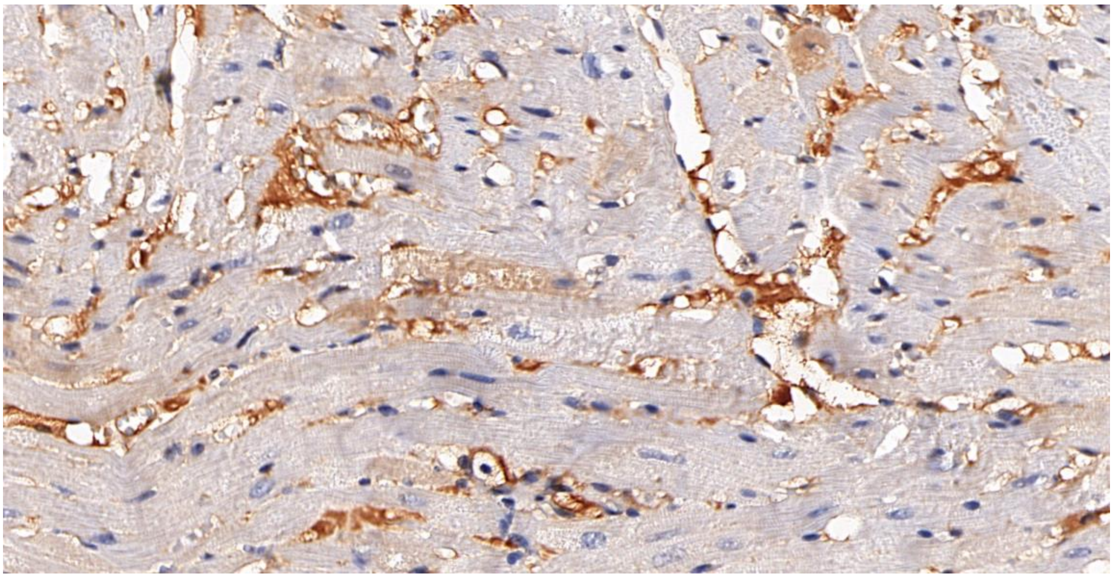

Ad-SC-shRNA

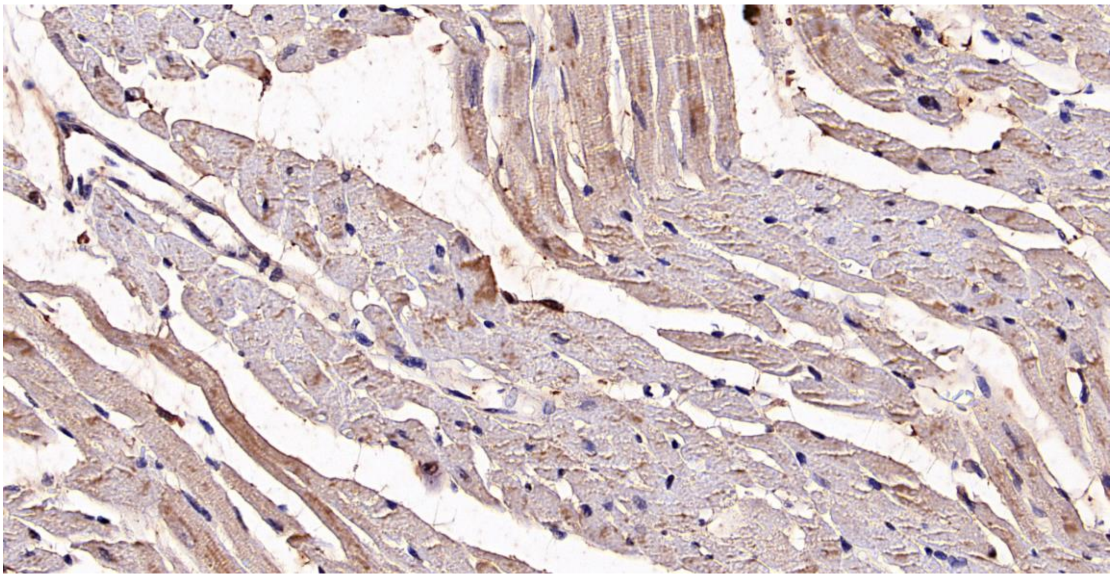

Ad-PRR-shRNA

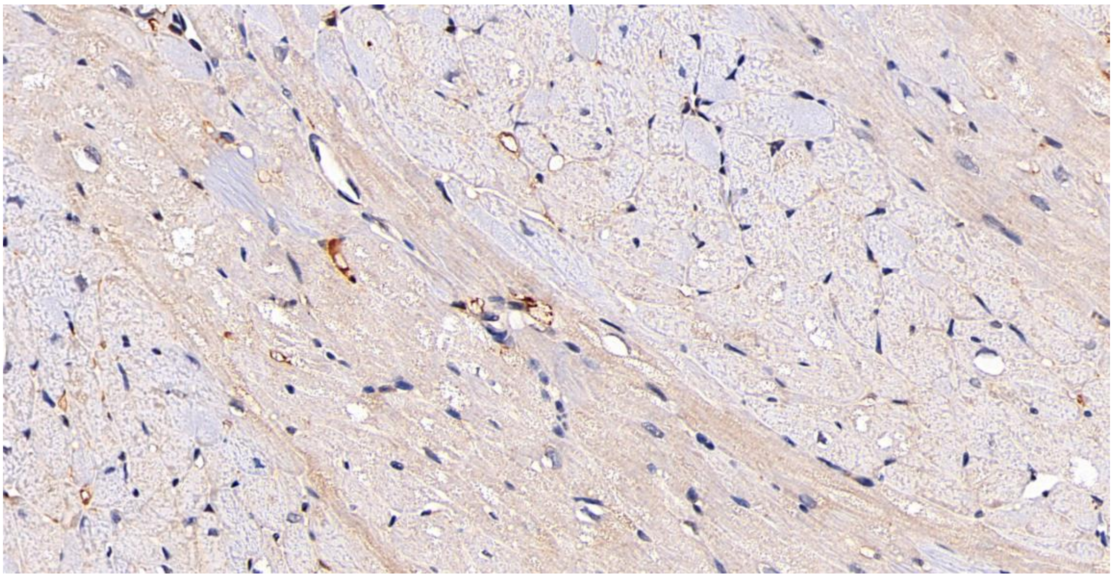

IL-18

Control

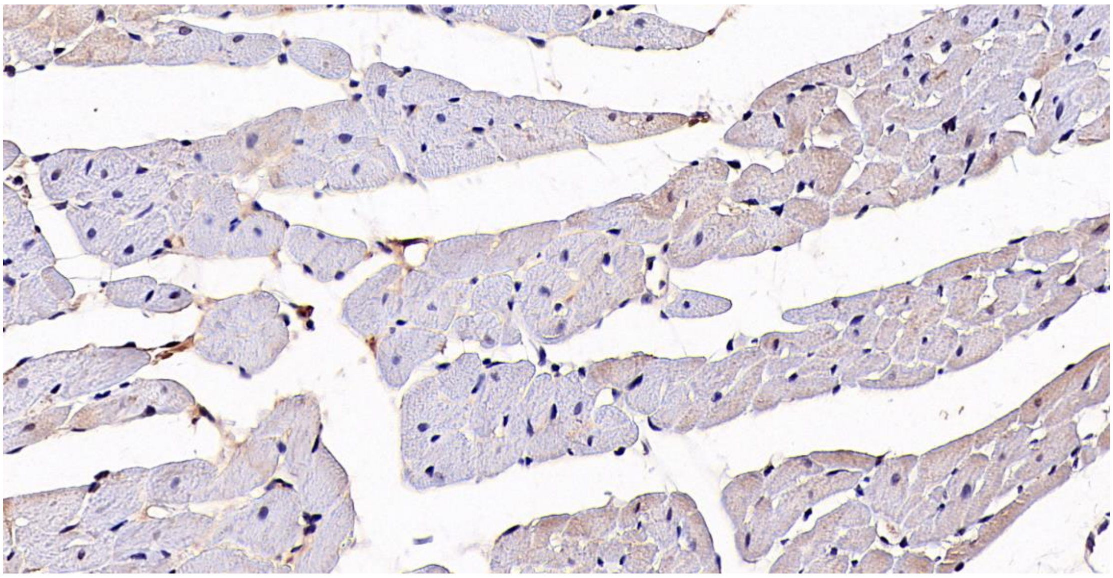

DCM

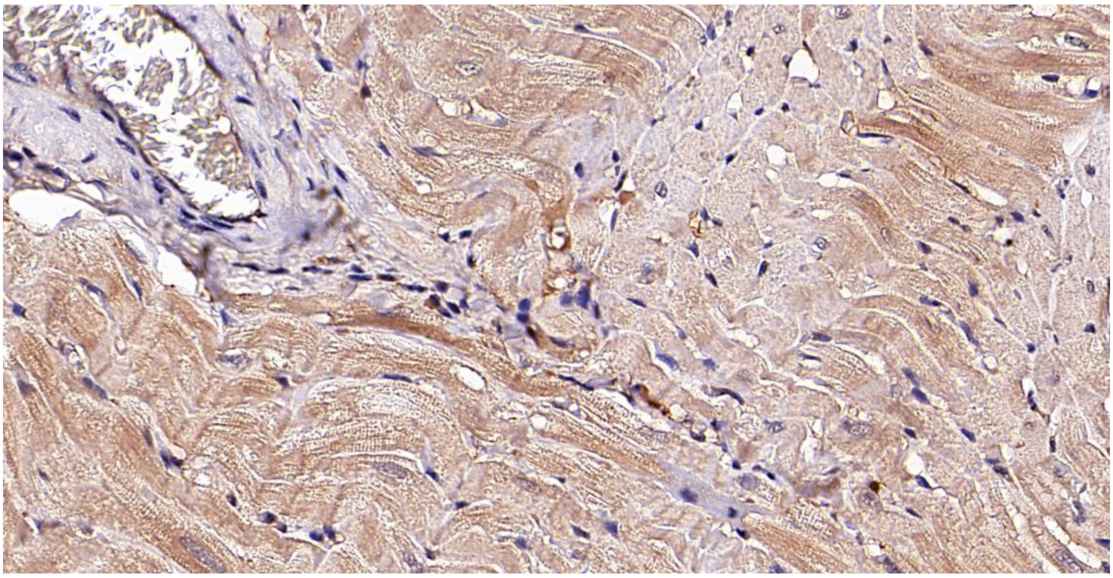

Ad-SC-shRNA

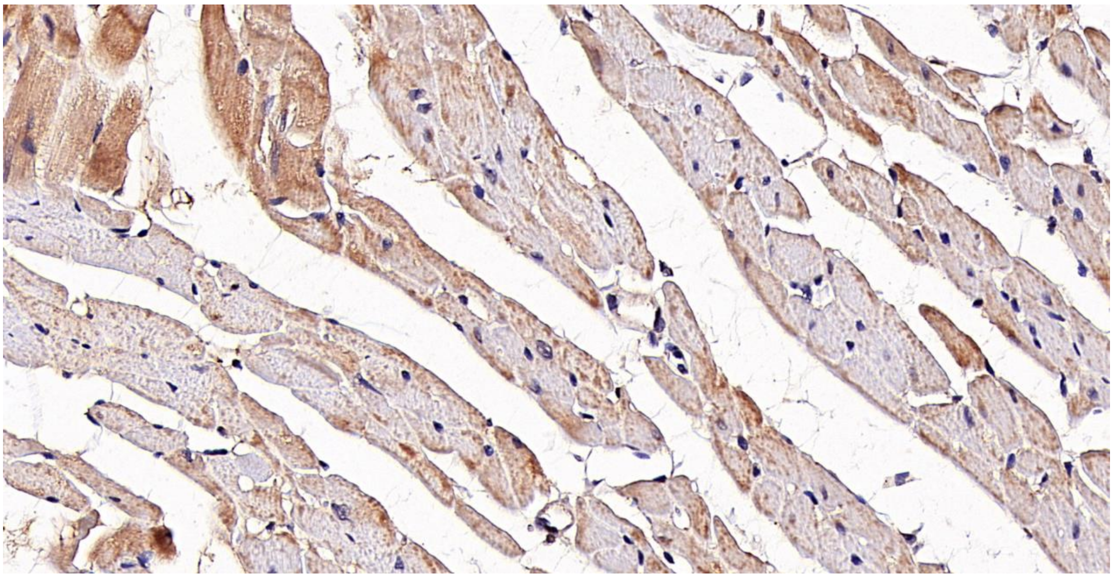

Ad-PRR-shRNA

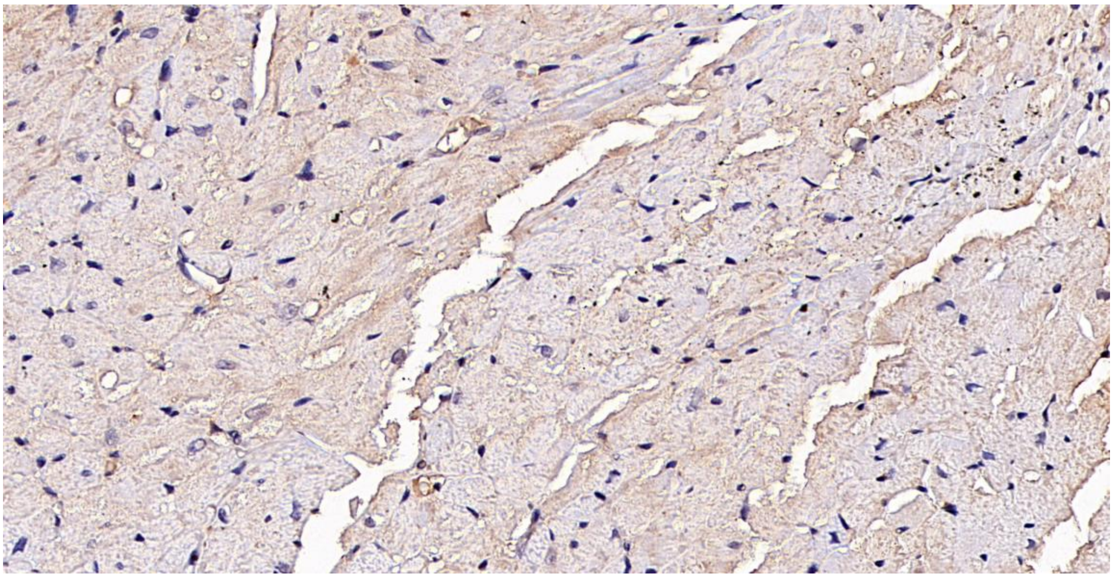

Figure3

HE staining

Control

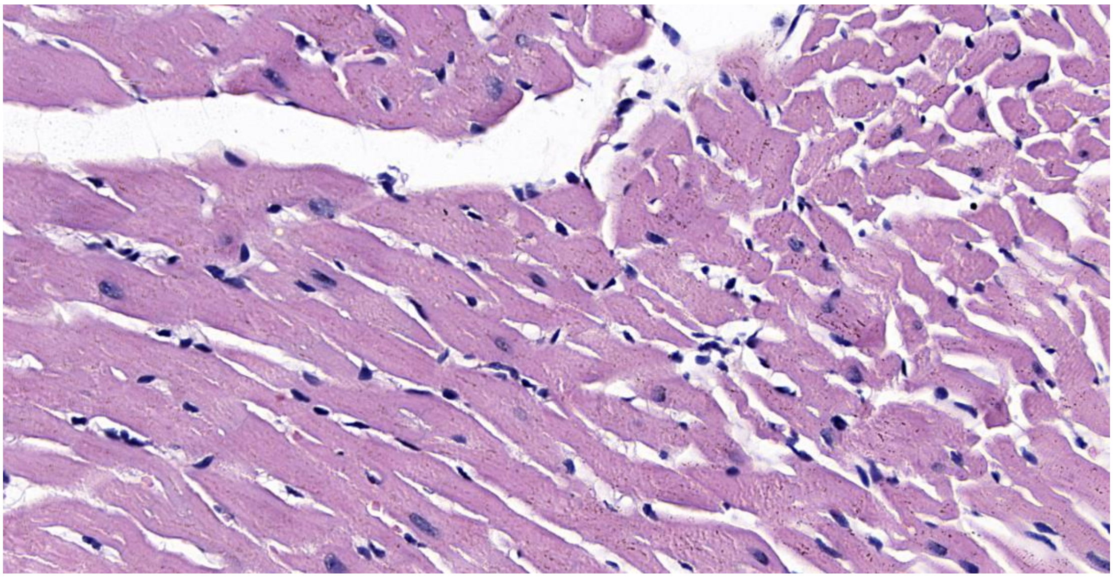

DCM

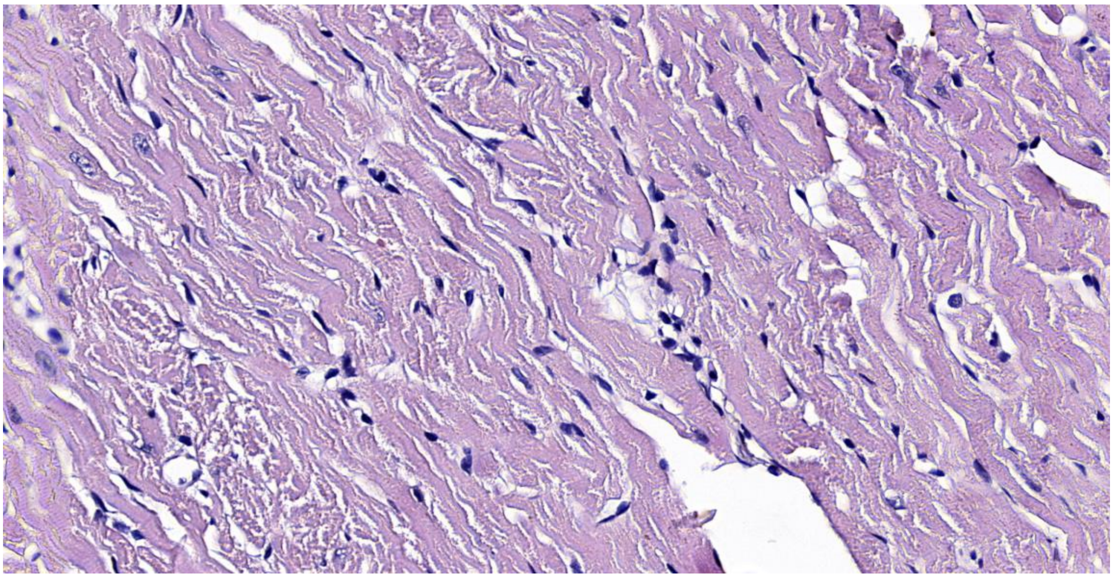

Ad-EGFP

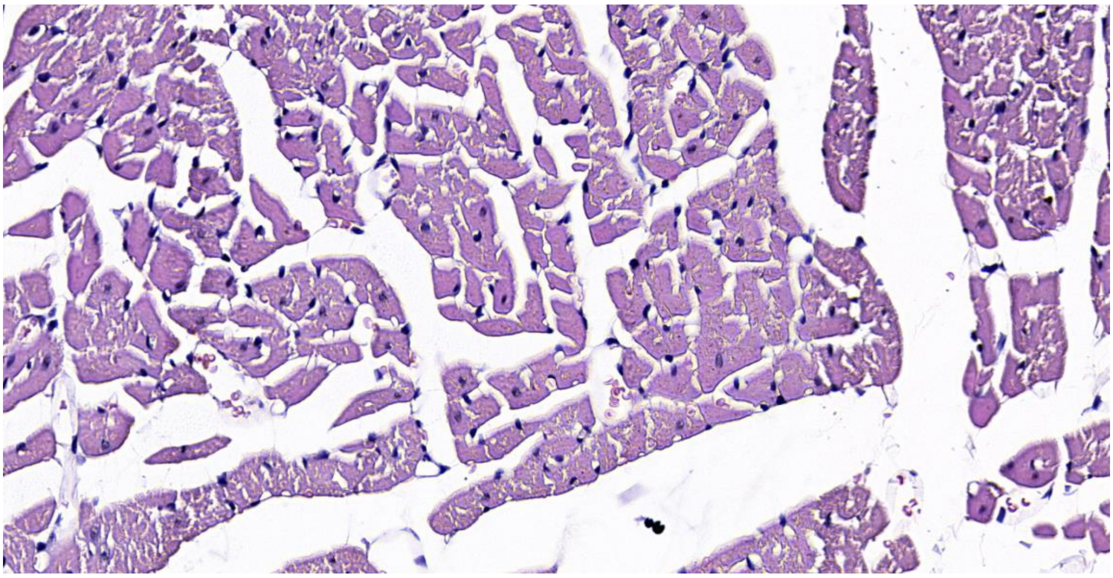

Ad-YAP

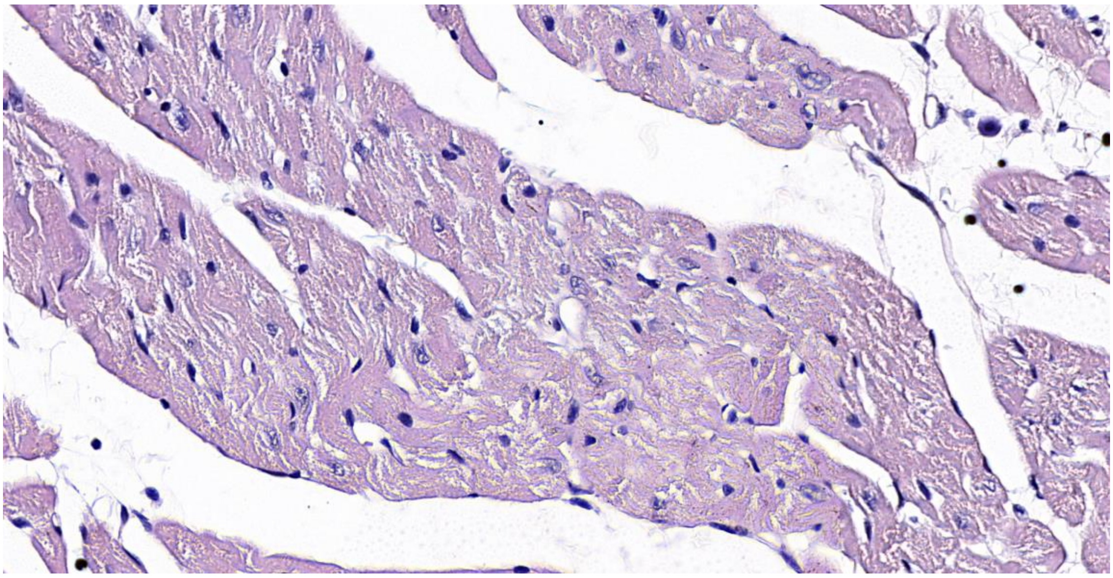

Immunohistochemical staining

YAP

Control

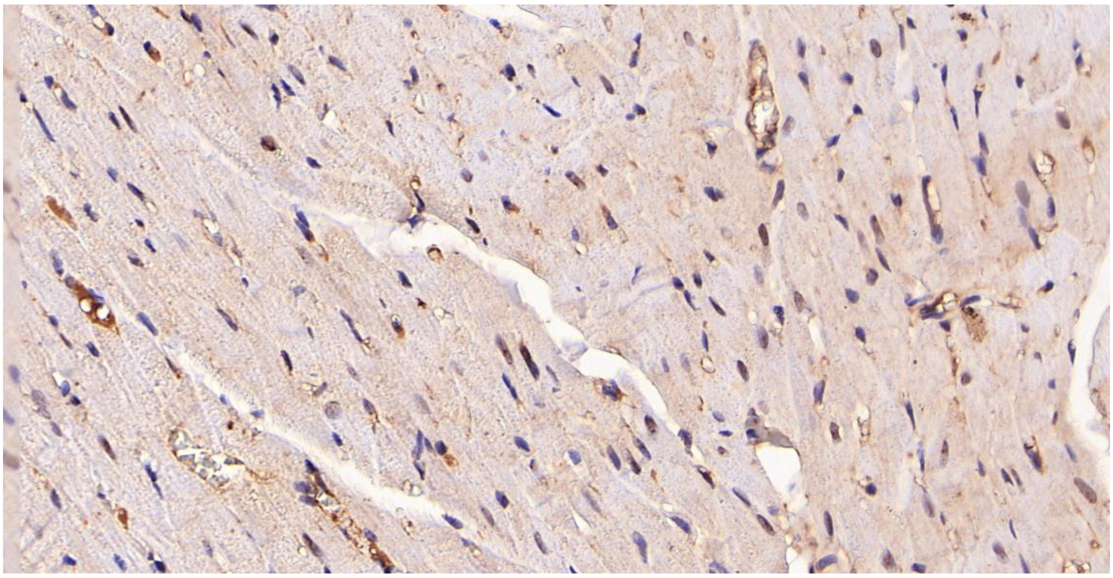

DCM

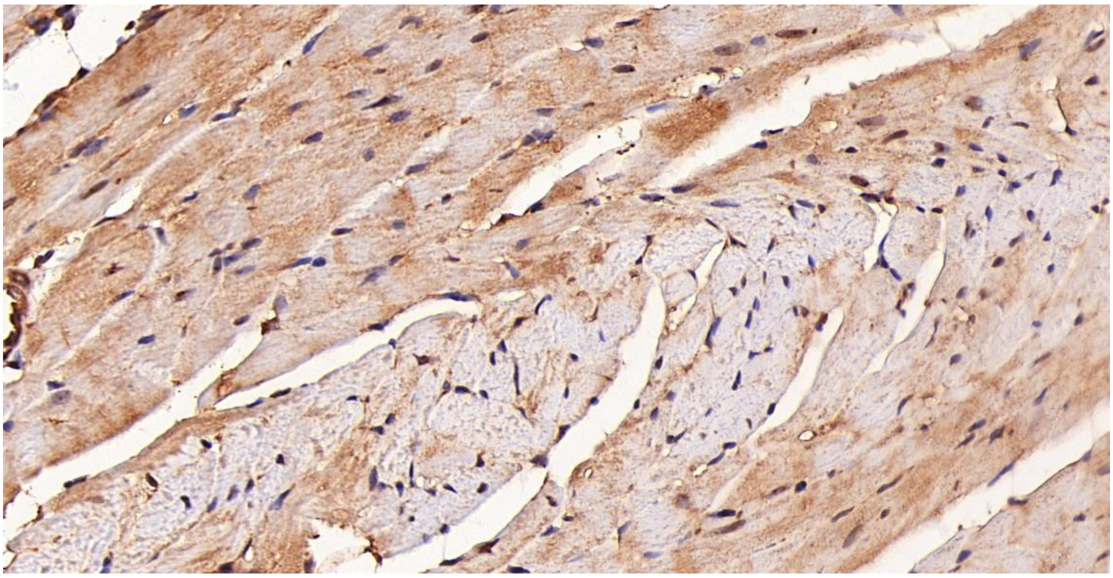

Ad-EGFP:

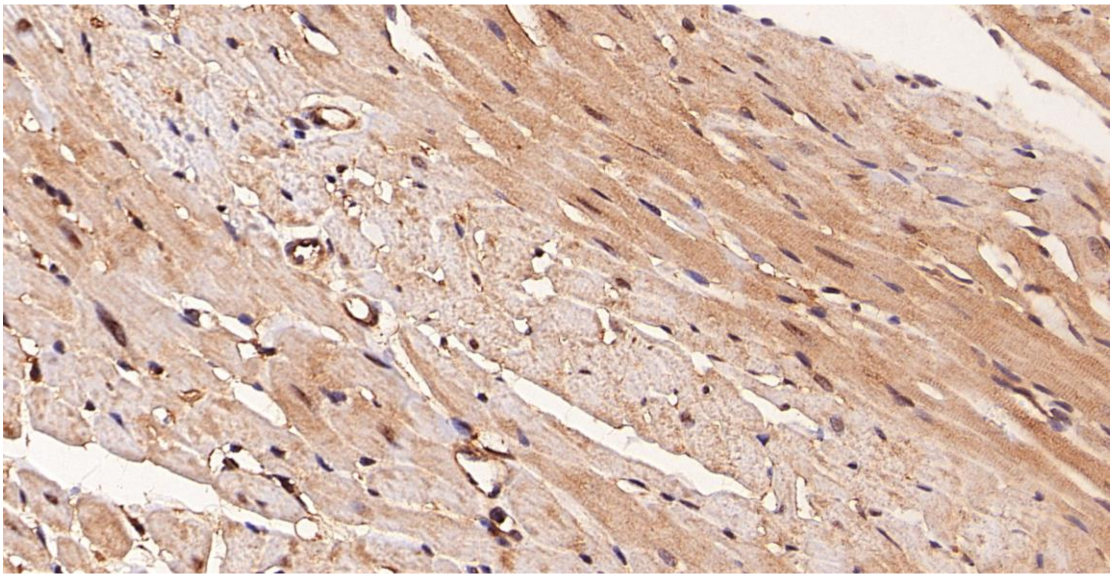

Ad-YAP

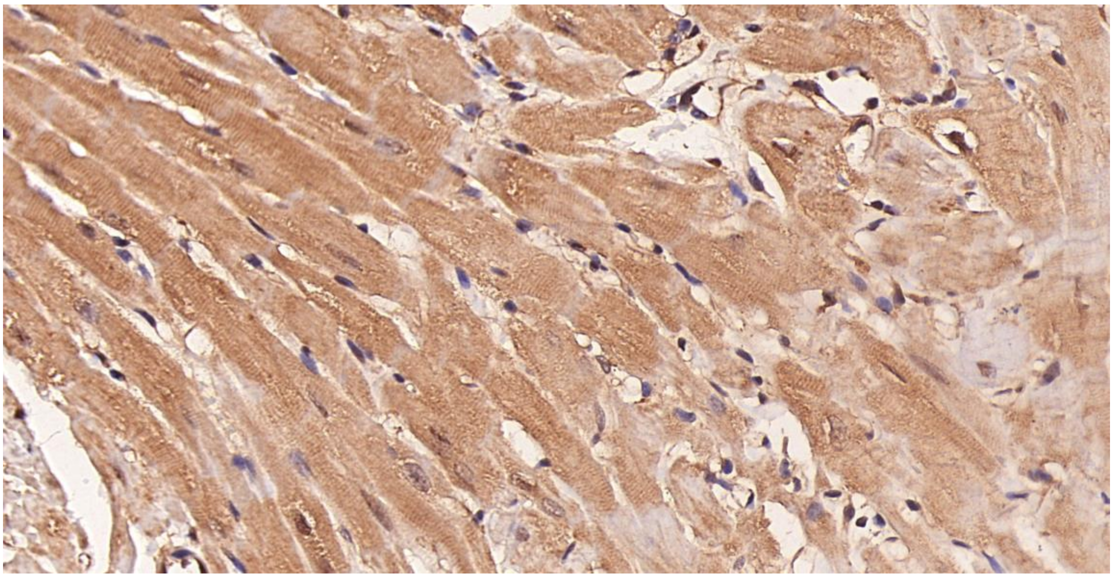

Collagen I

Control

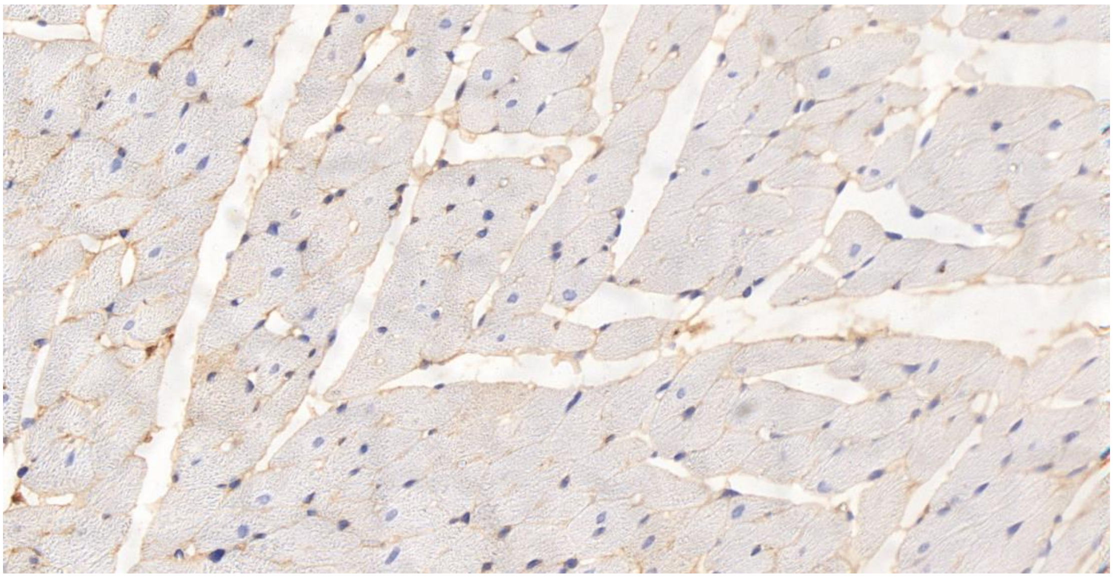

DCM

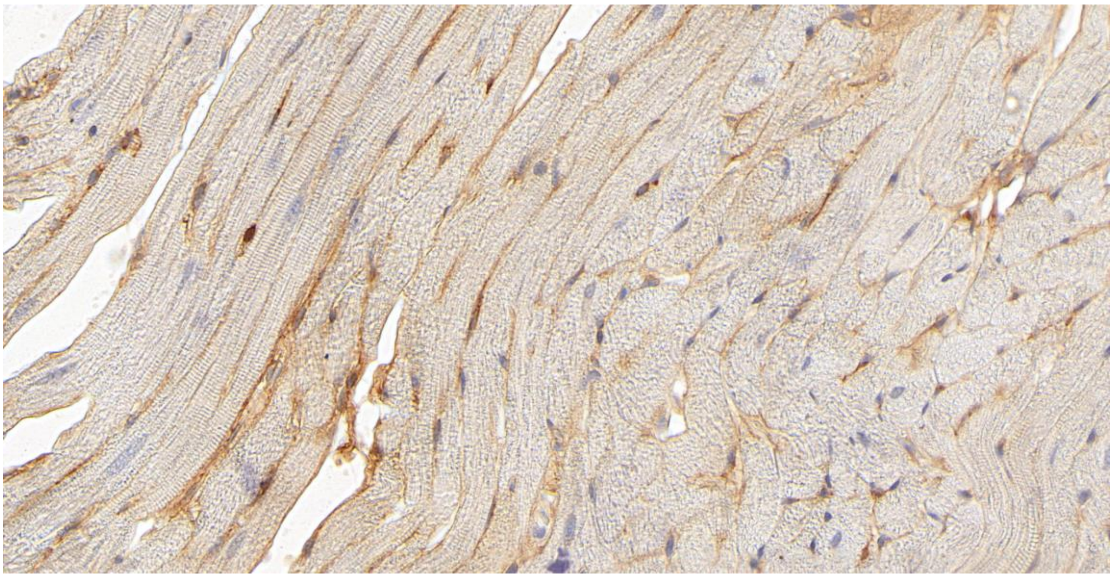

Ad-EGFP

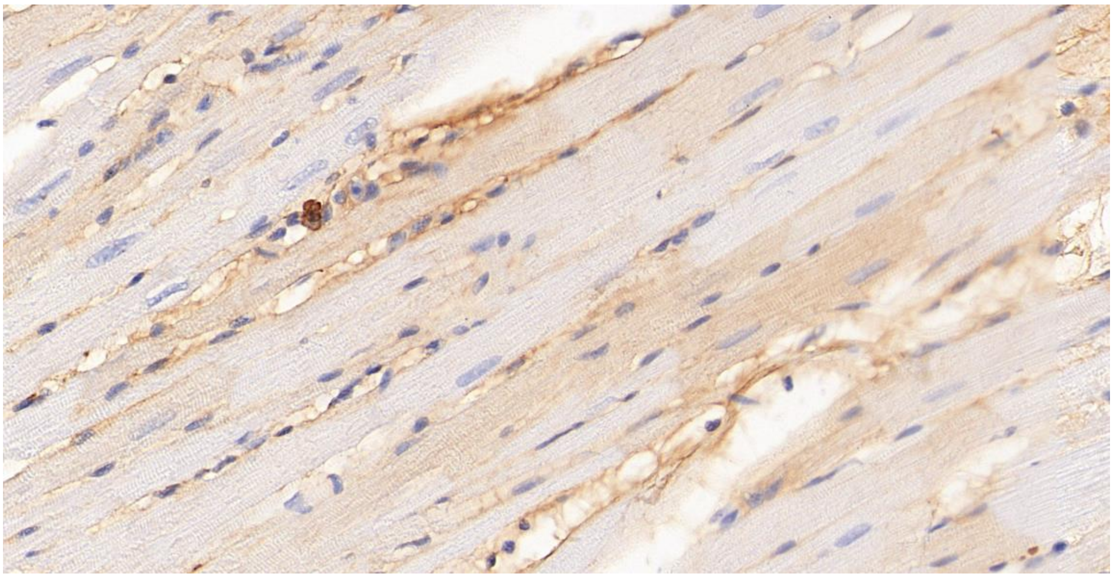

Ad-YAP

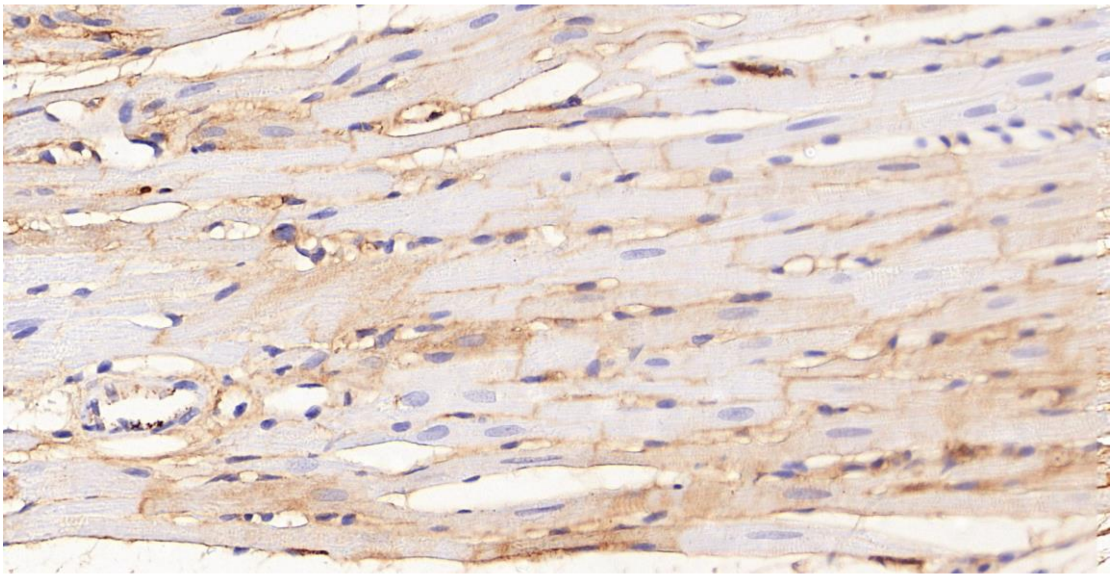

Proflin-1

Control

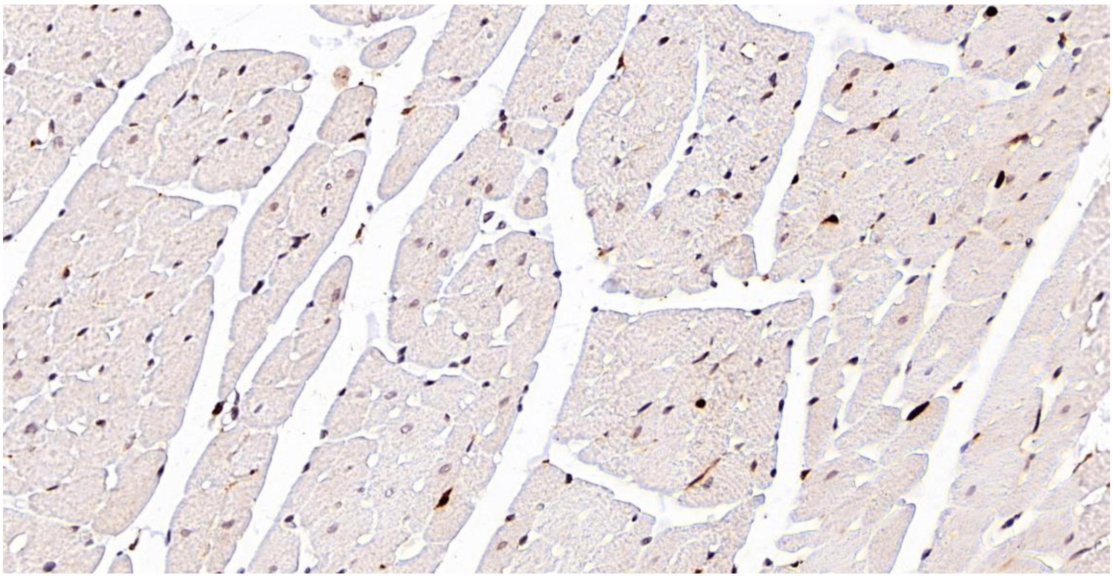

DCM

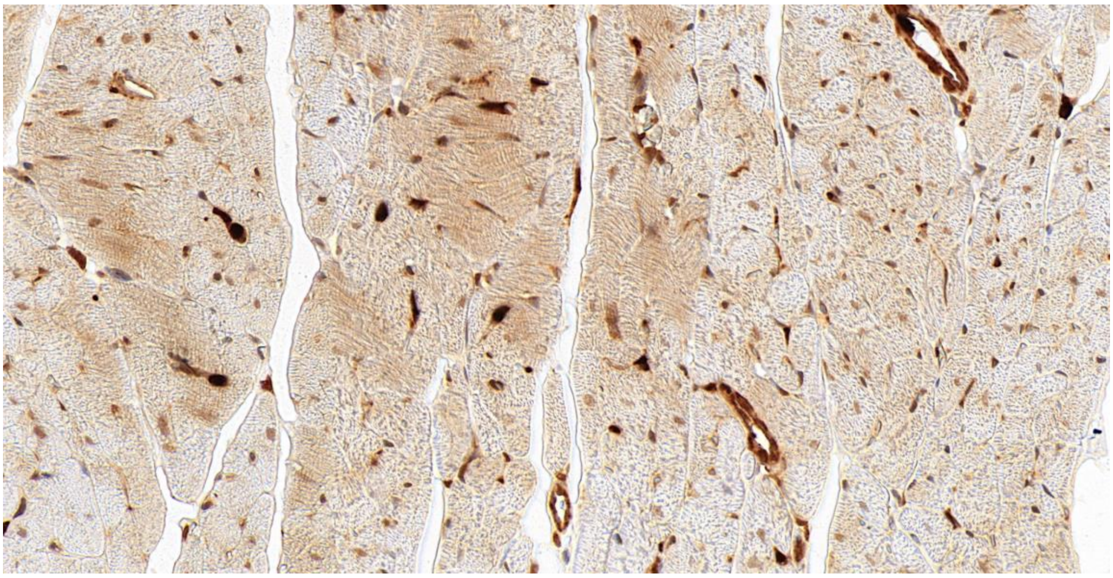

Ad-EGFP

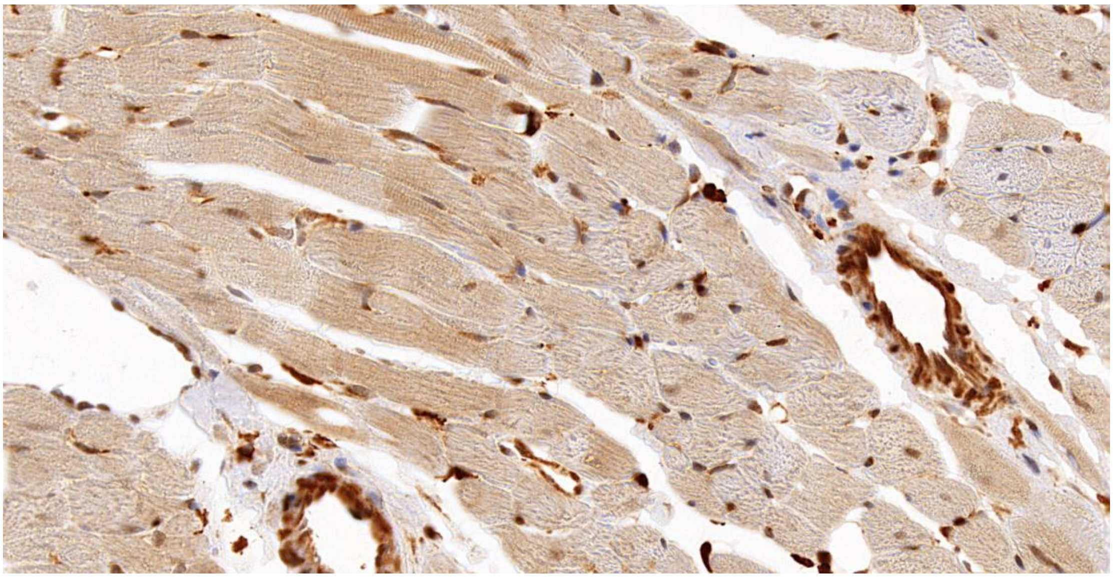

Ad-YAP

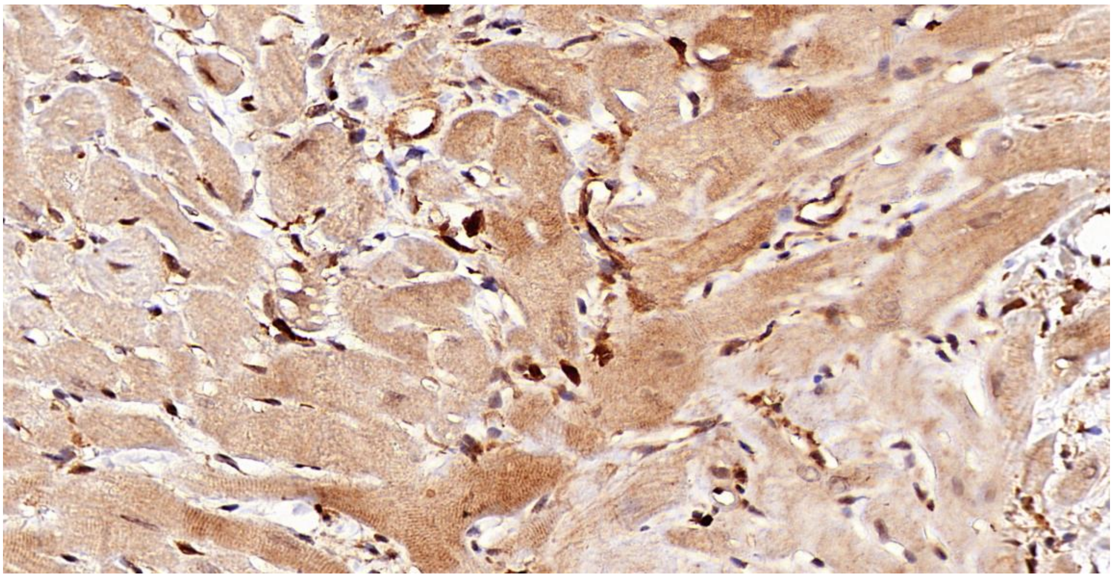

IL-8

Control

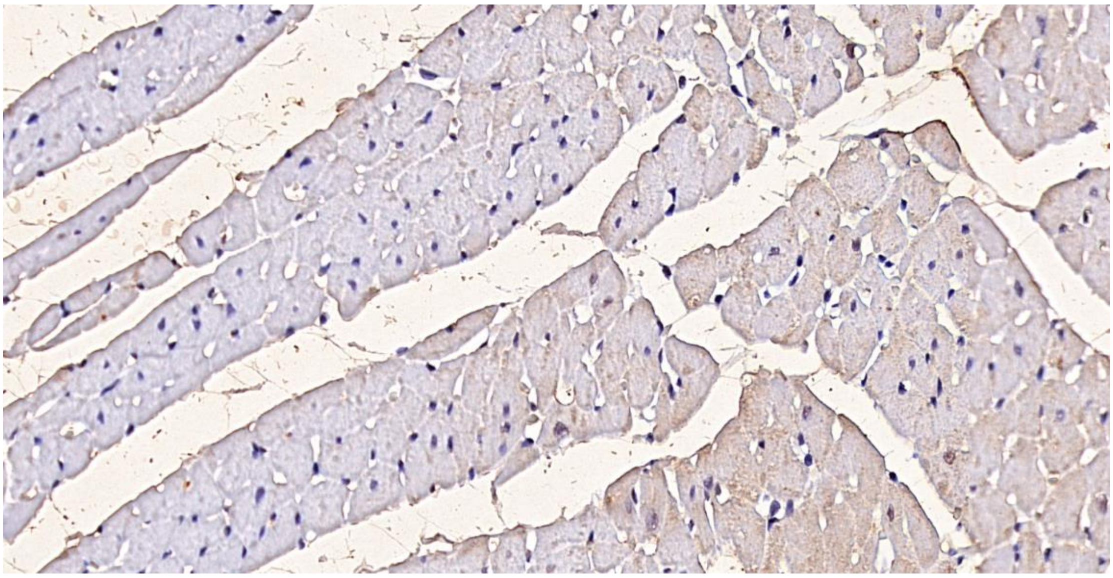

DCM

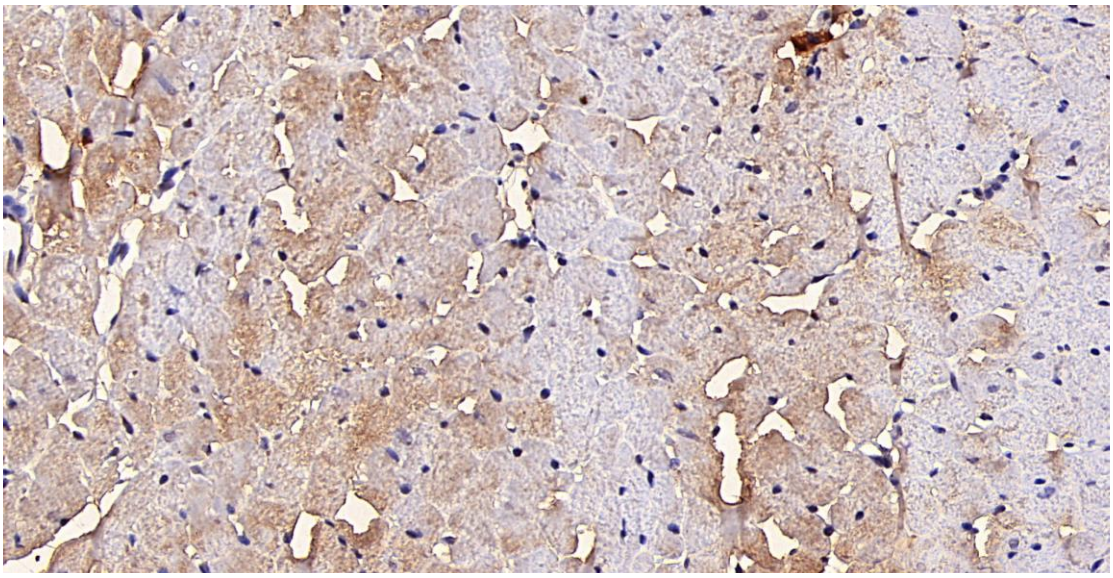

Ad-EGFP:

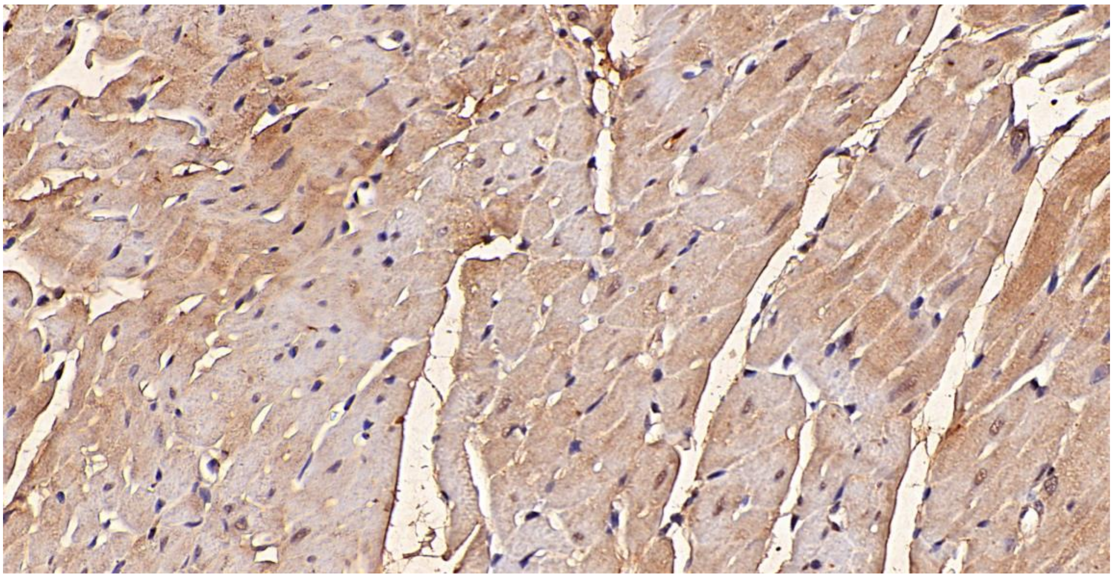

Ad-YAP

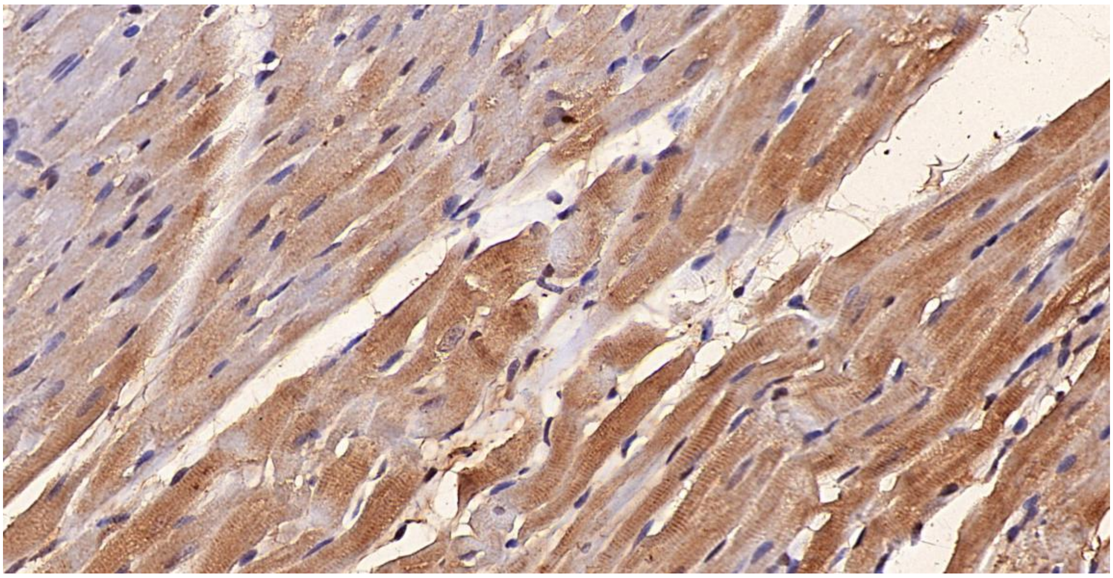

IL-18

Control

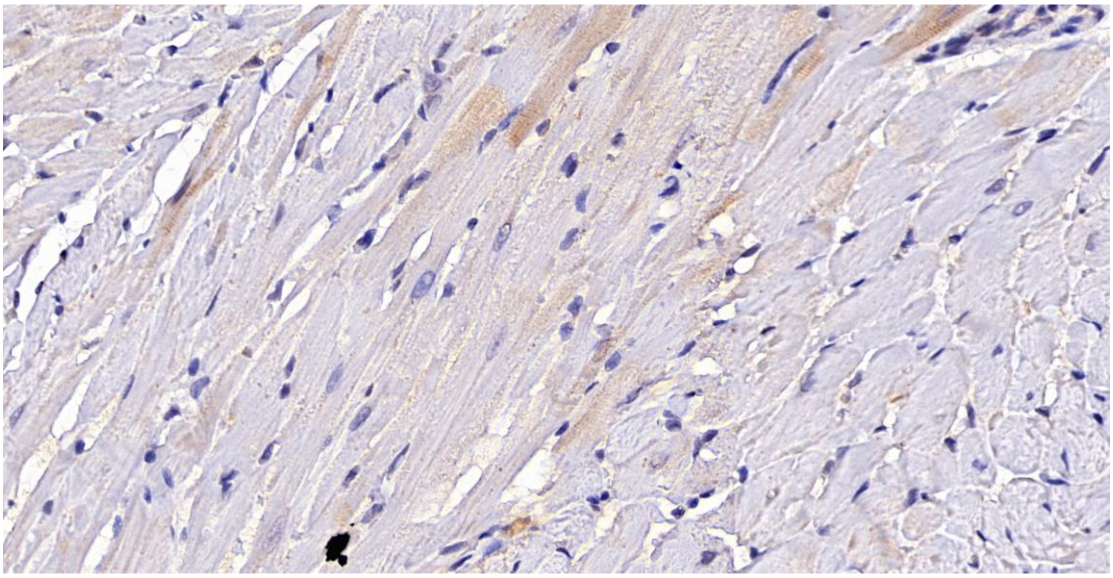

DCM:

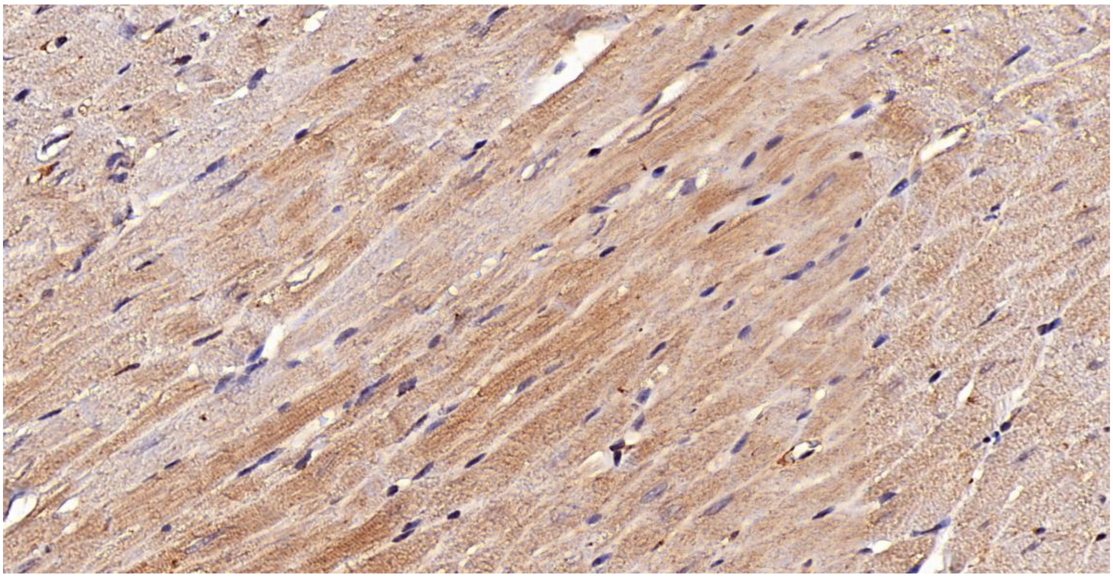

Ad-EGFP

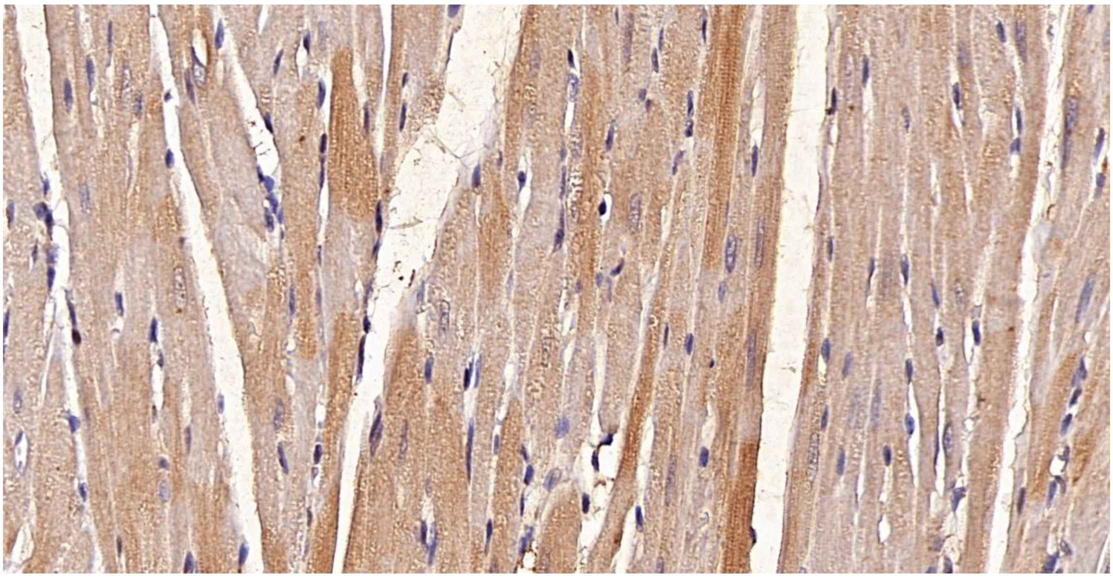

Ad-YAP

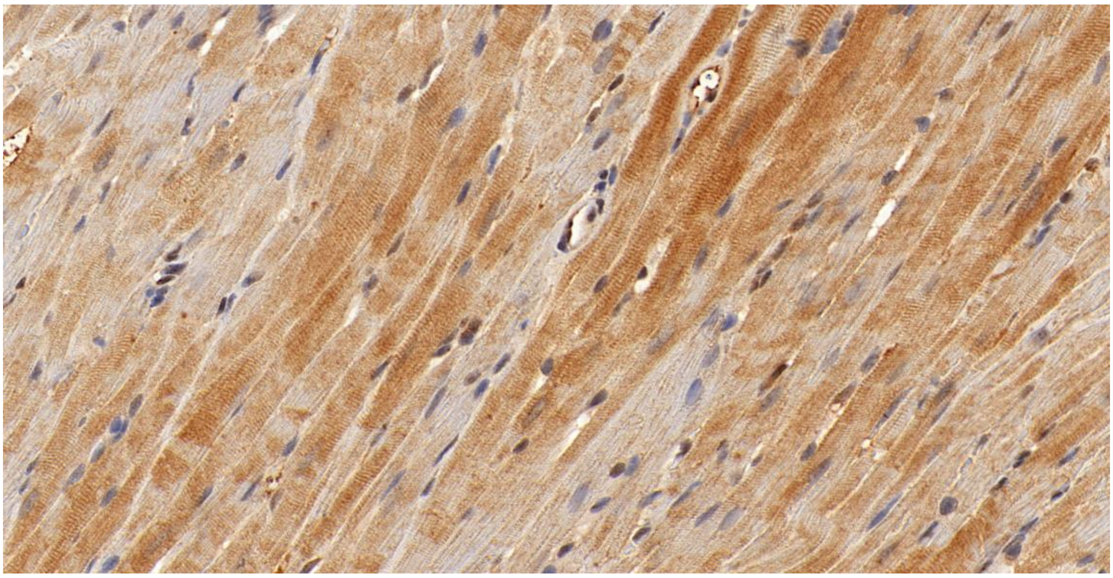

Figure4

HE

Control

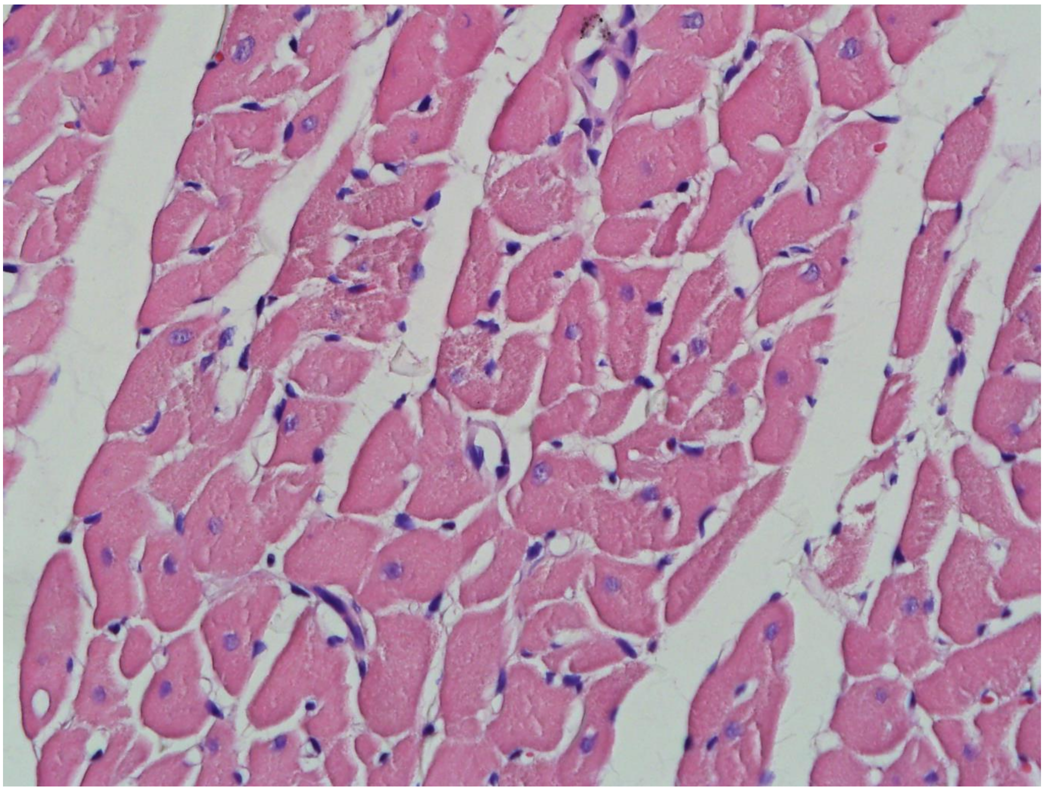

DCM

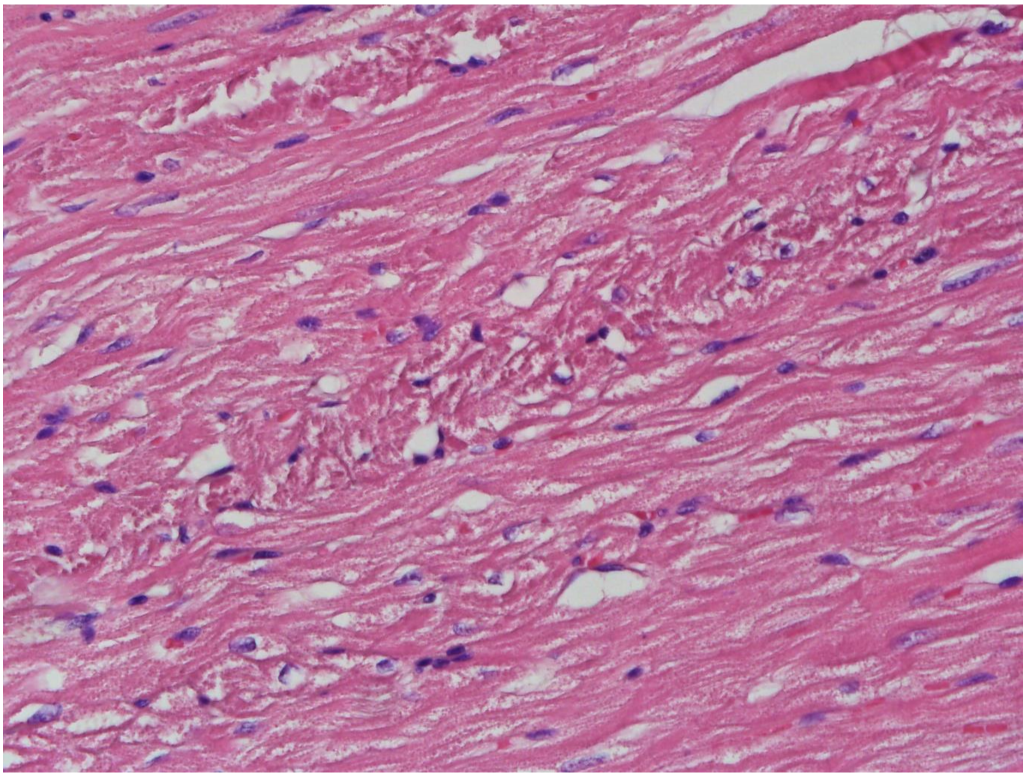

LV-SC-shRNA

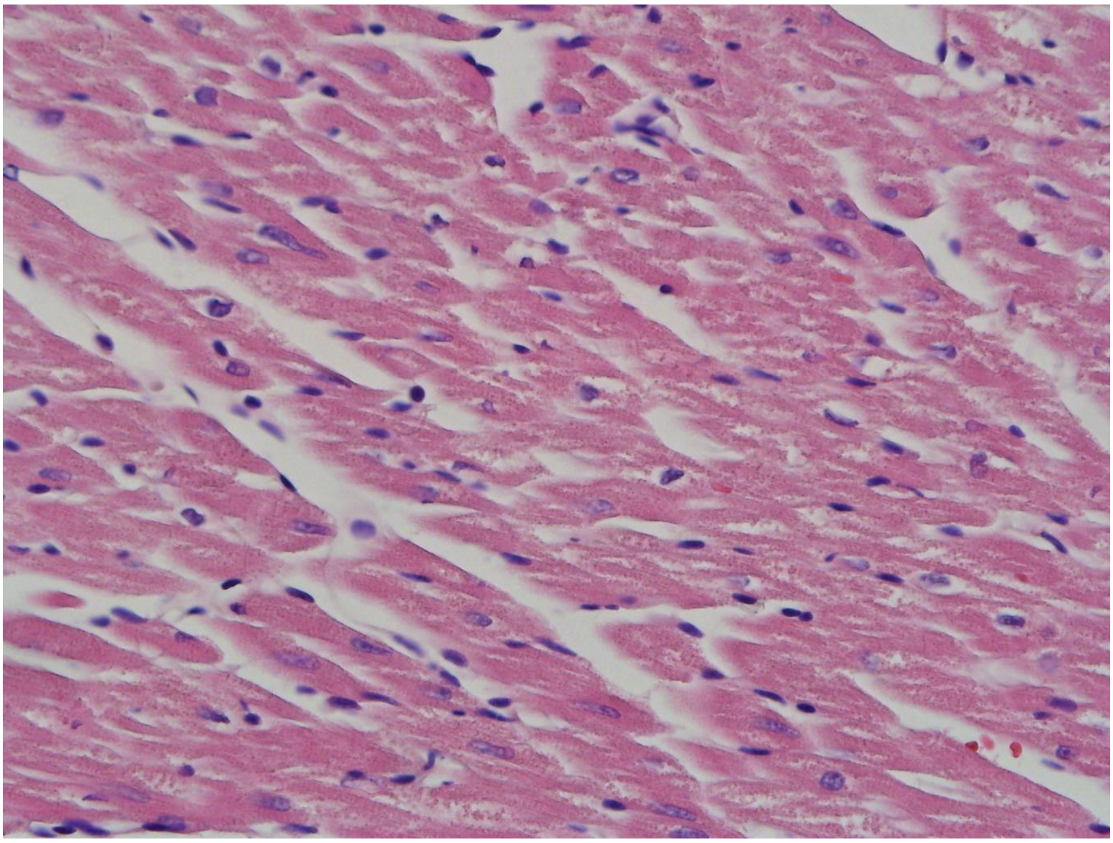

LV-YAP-shRNA

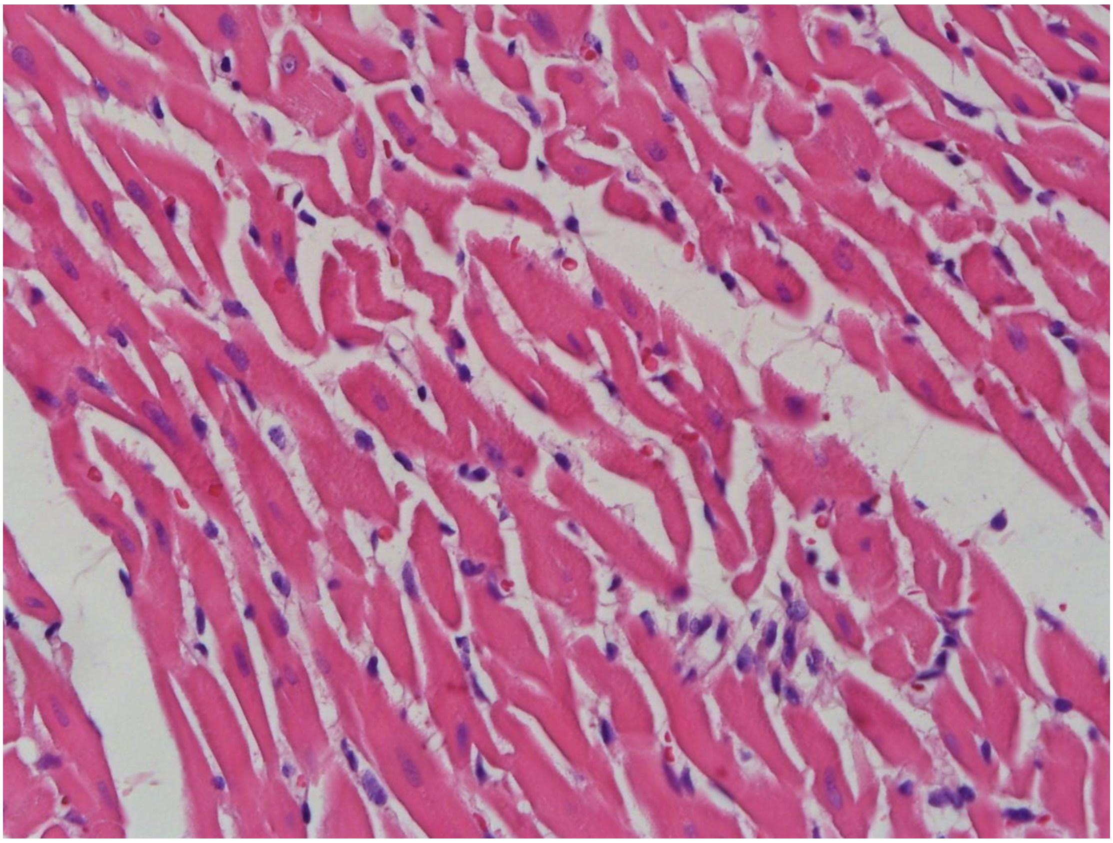

Immumohistochemical staining
YAP

Control

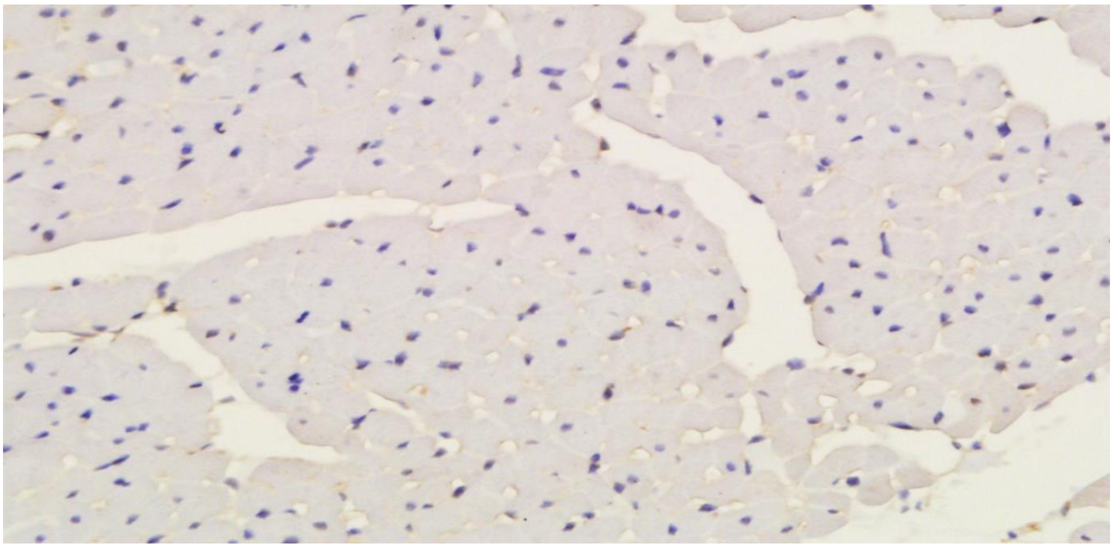

DCM

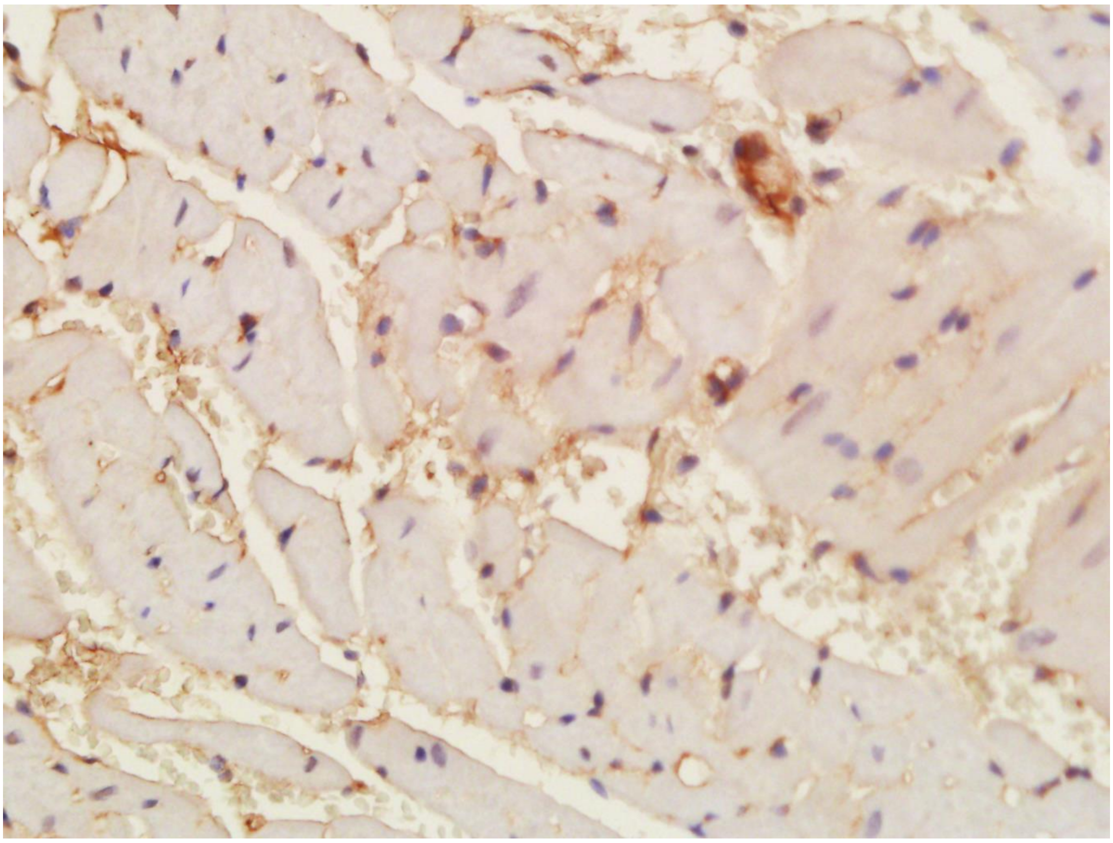

LV-SC-shRNA:

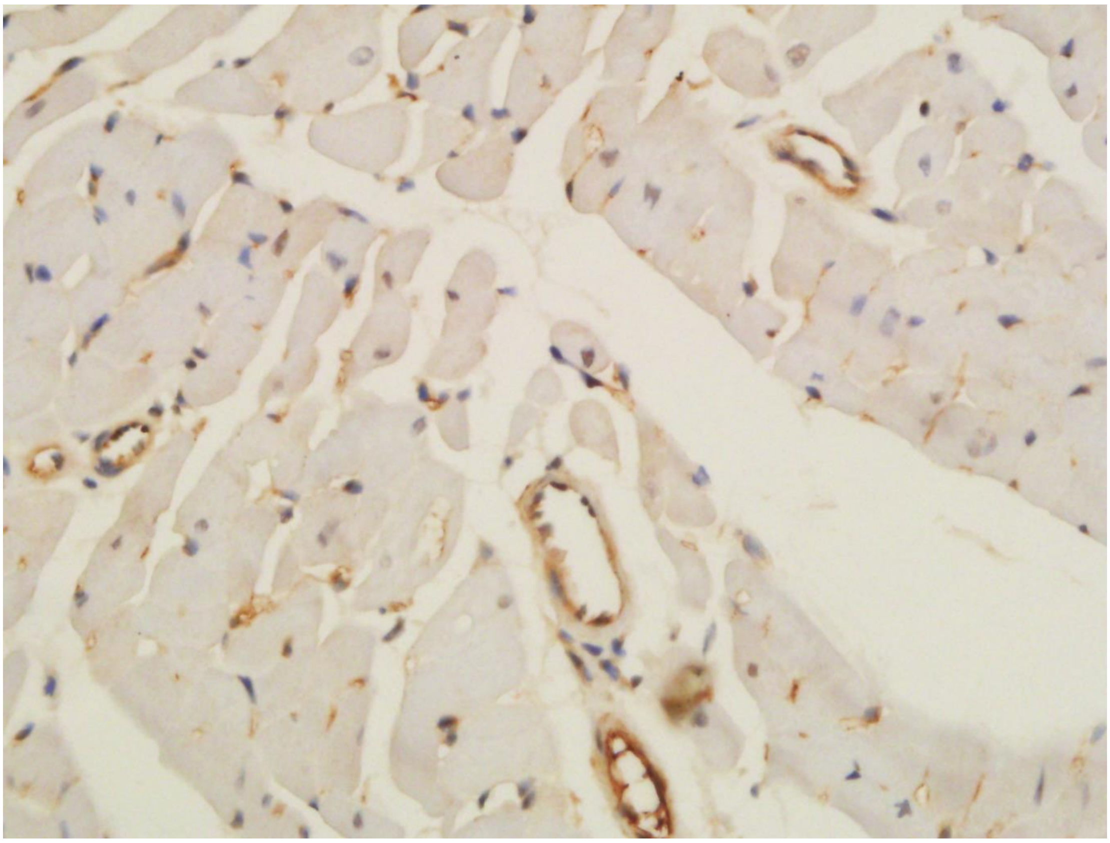

LV-YAP-shRNA

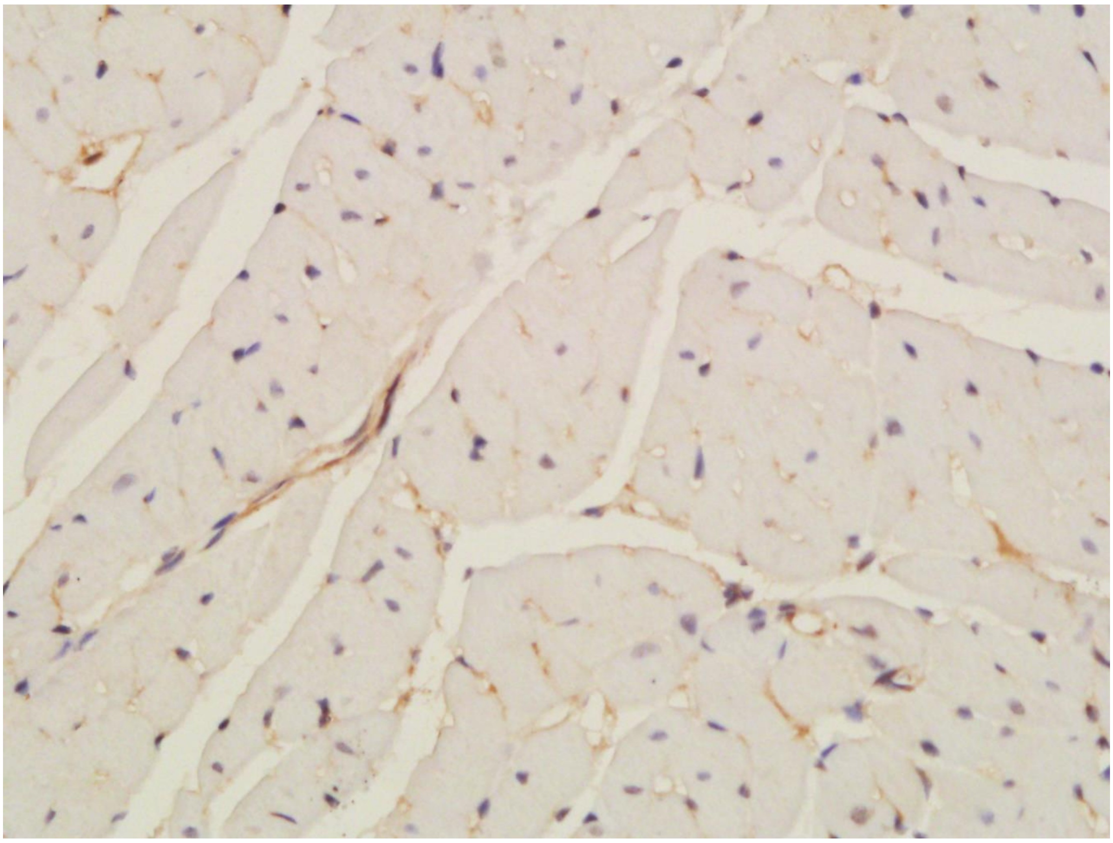

Control

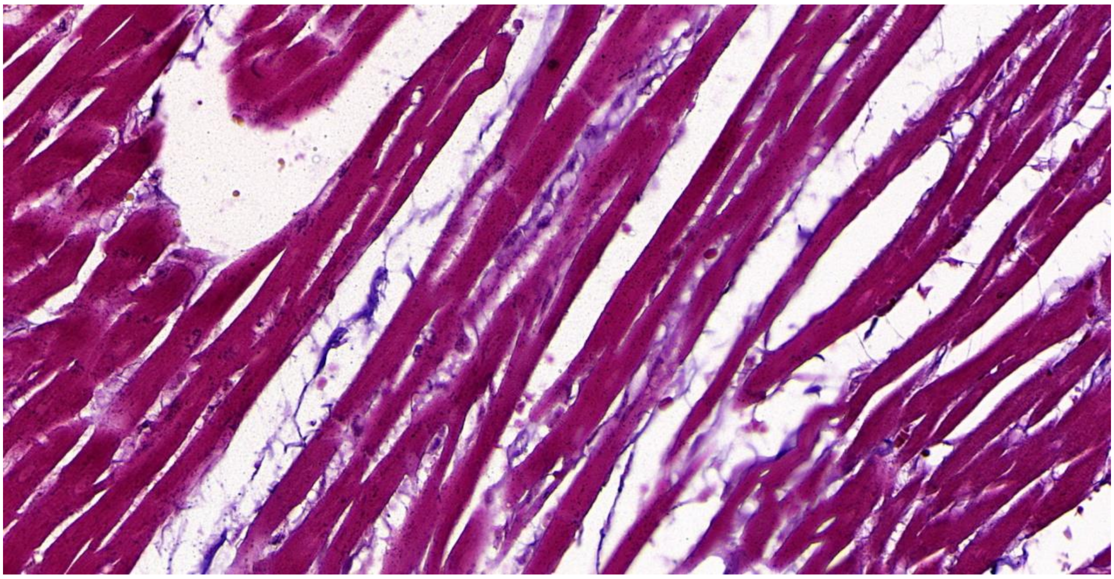

DCM

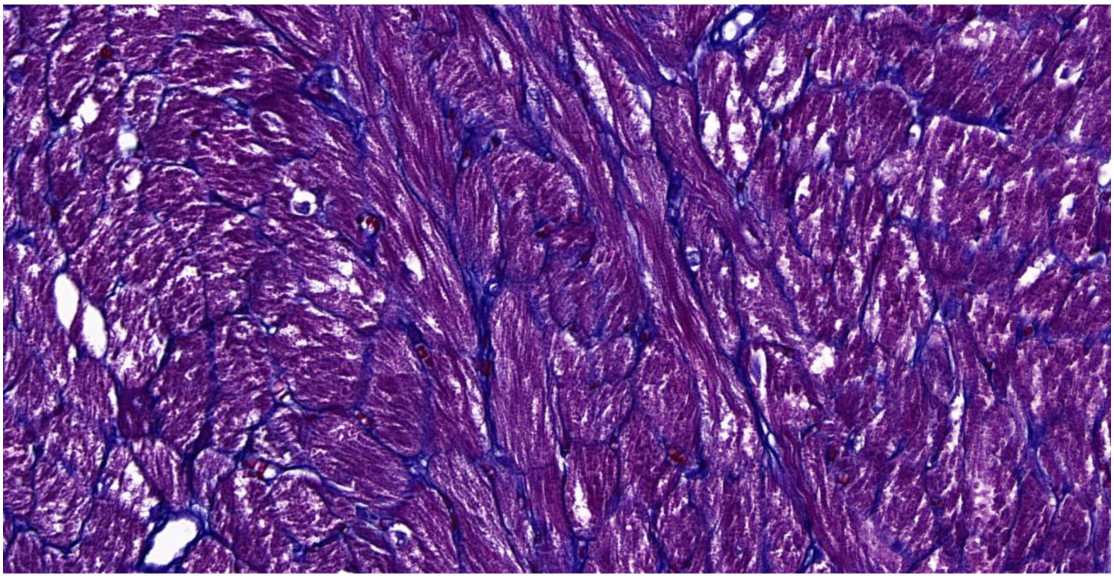

LV-SC-shRNA:

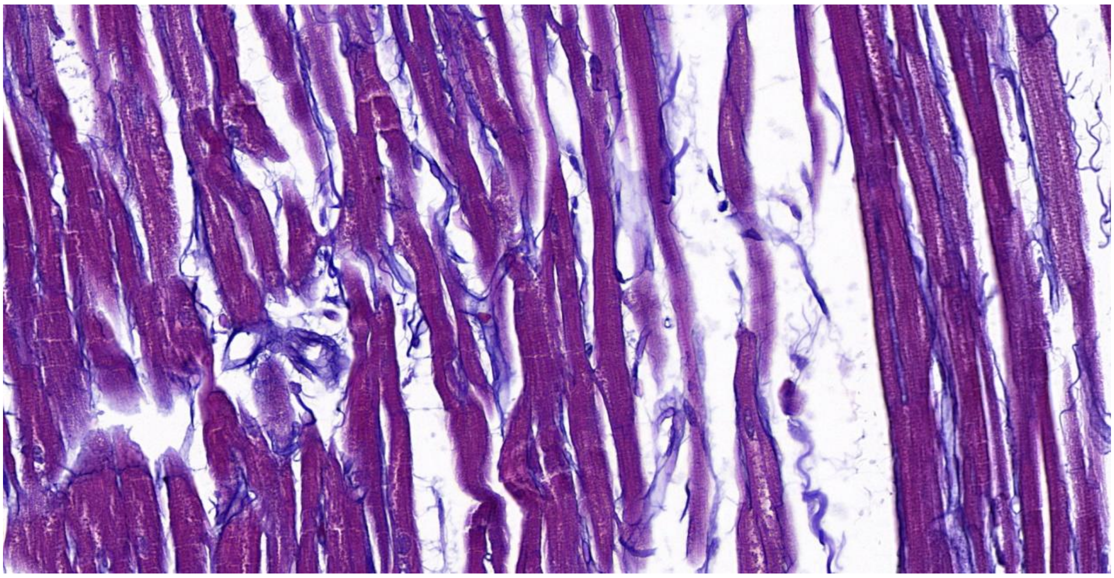

LV-YAP-shRNA:

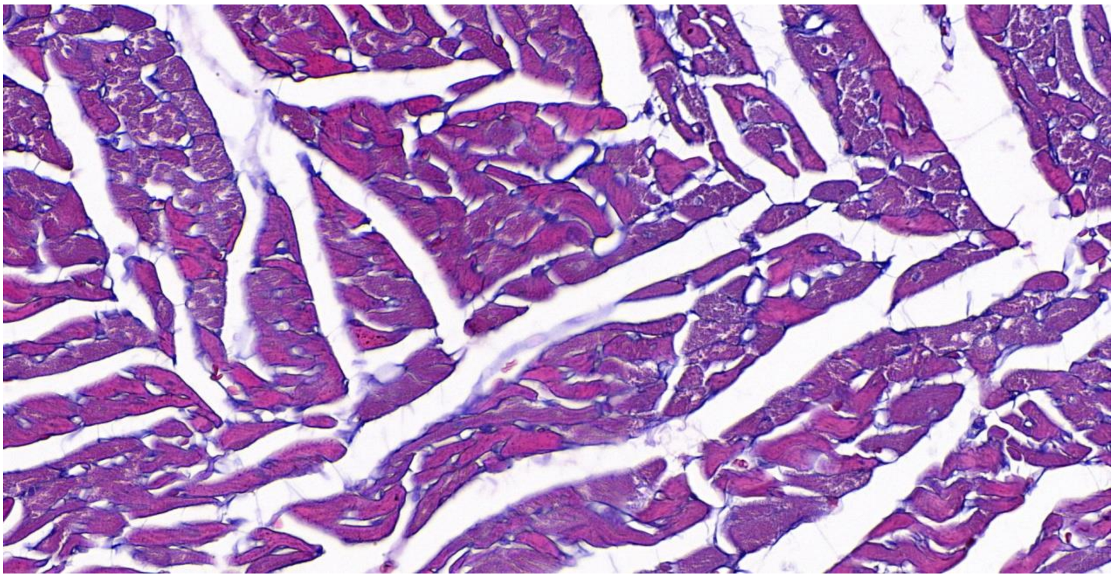

Fibronectin：

Control:

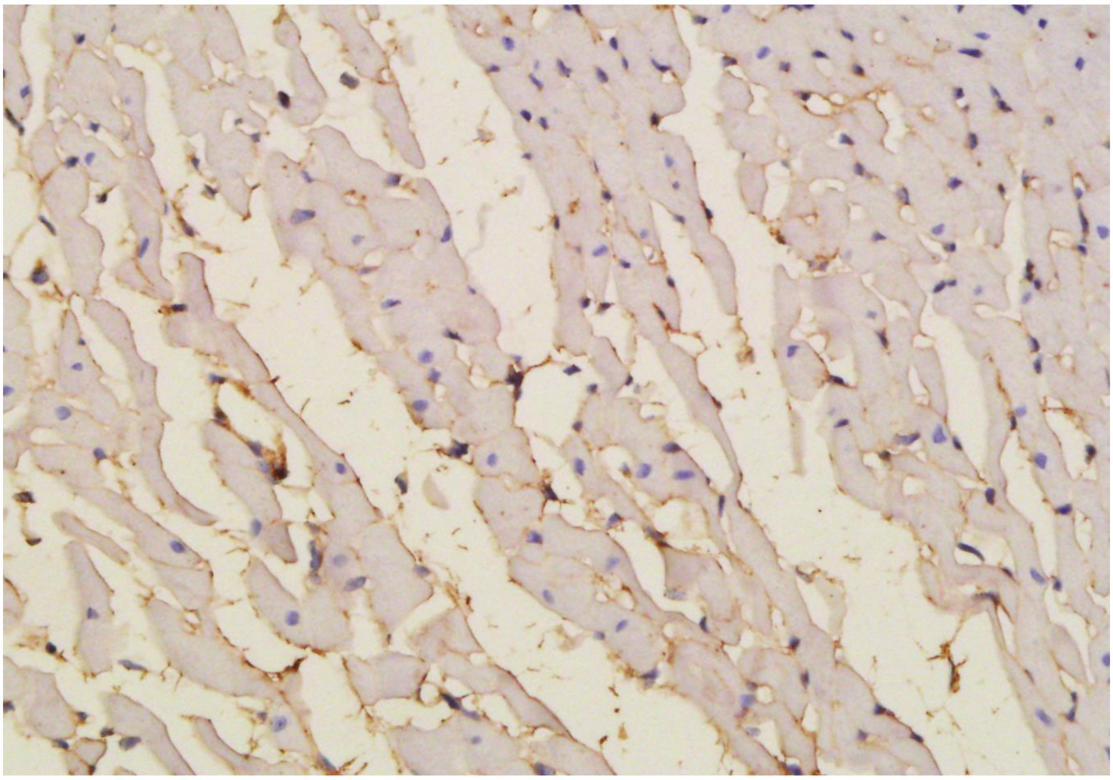

DCM

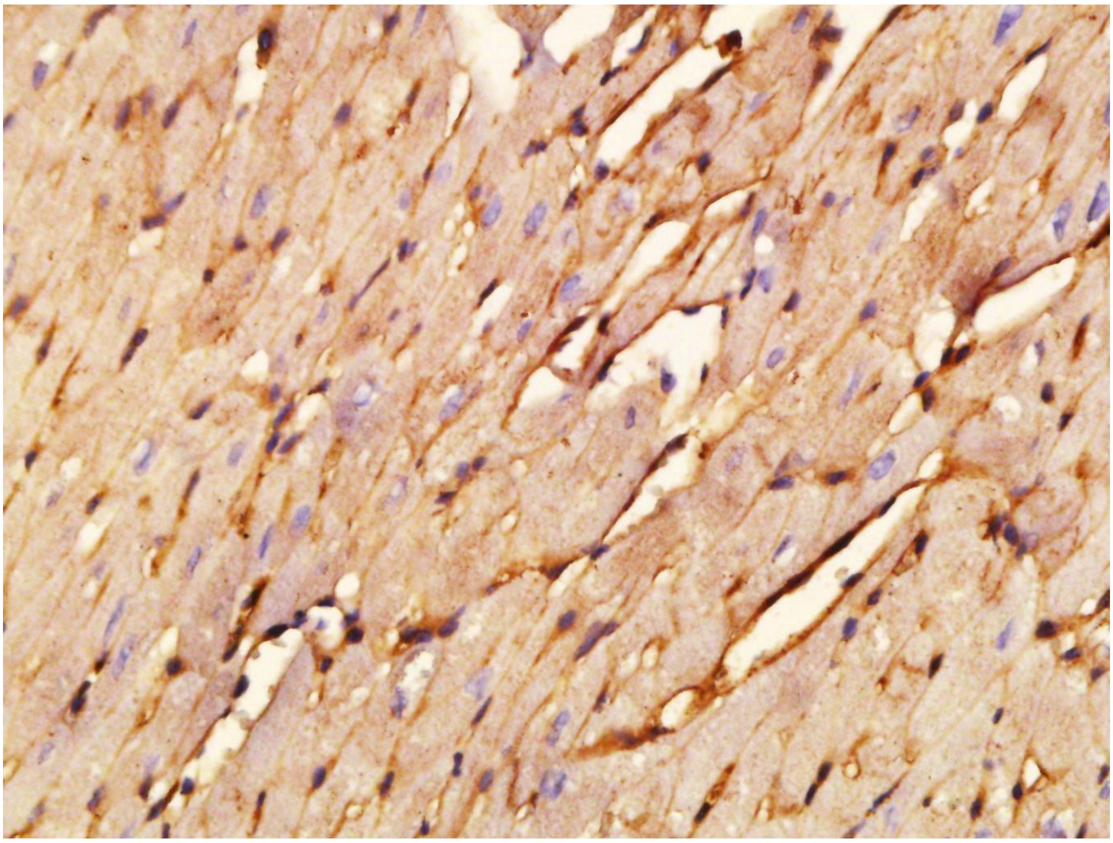

LV-SC-shRNA:

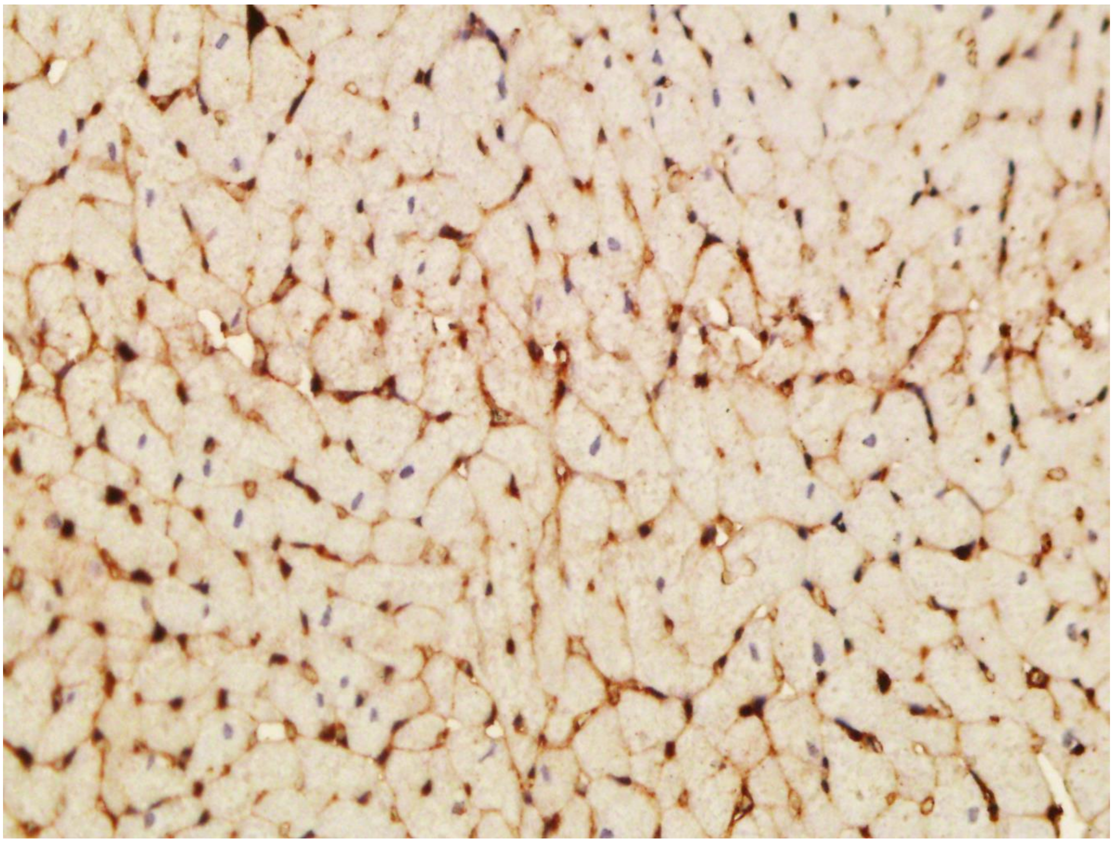

LV-YAP-shRNA:

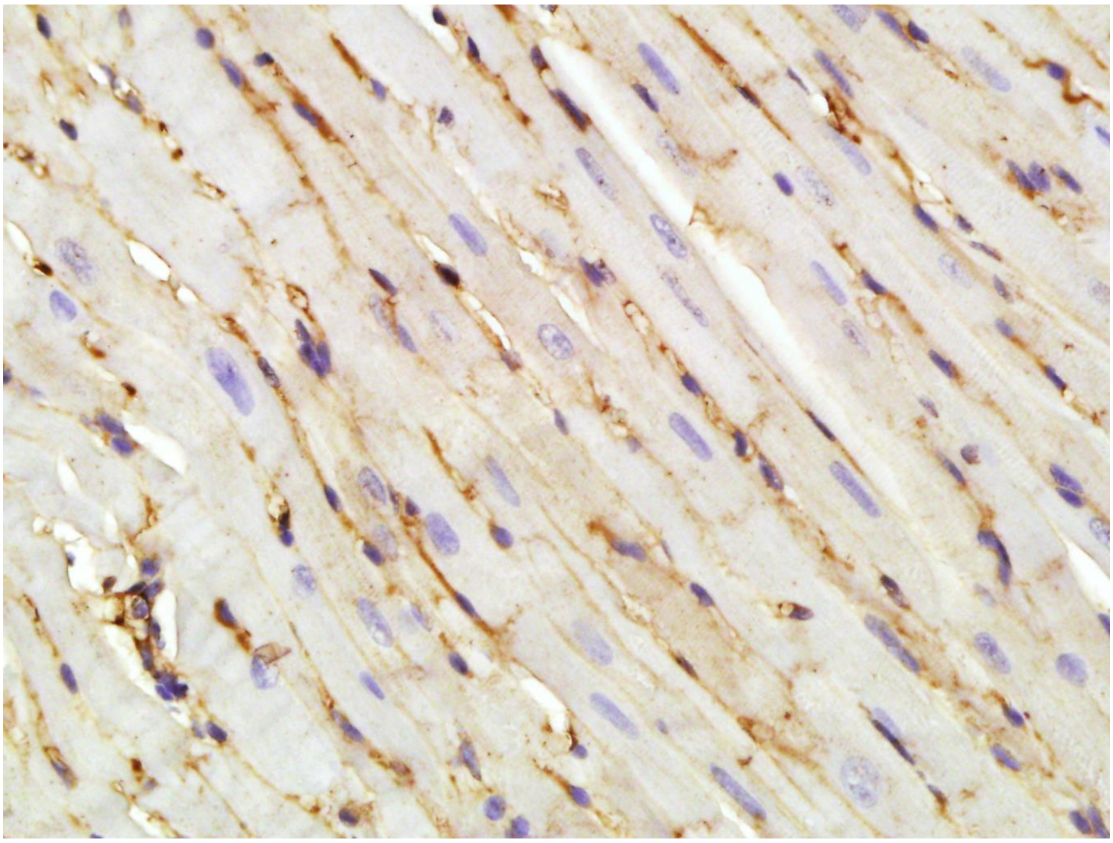

IL-1β
Control

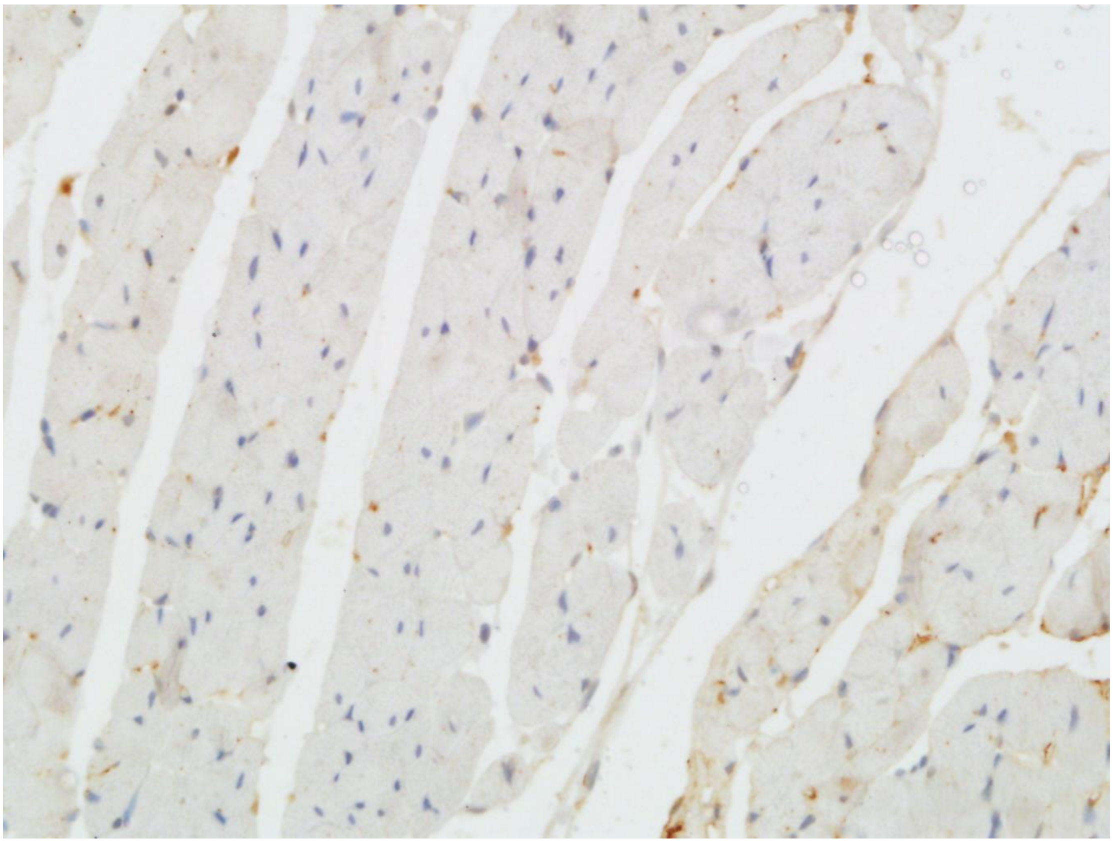

DCM

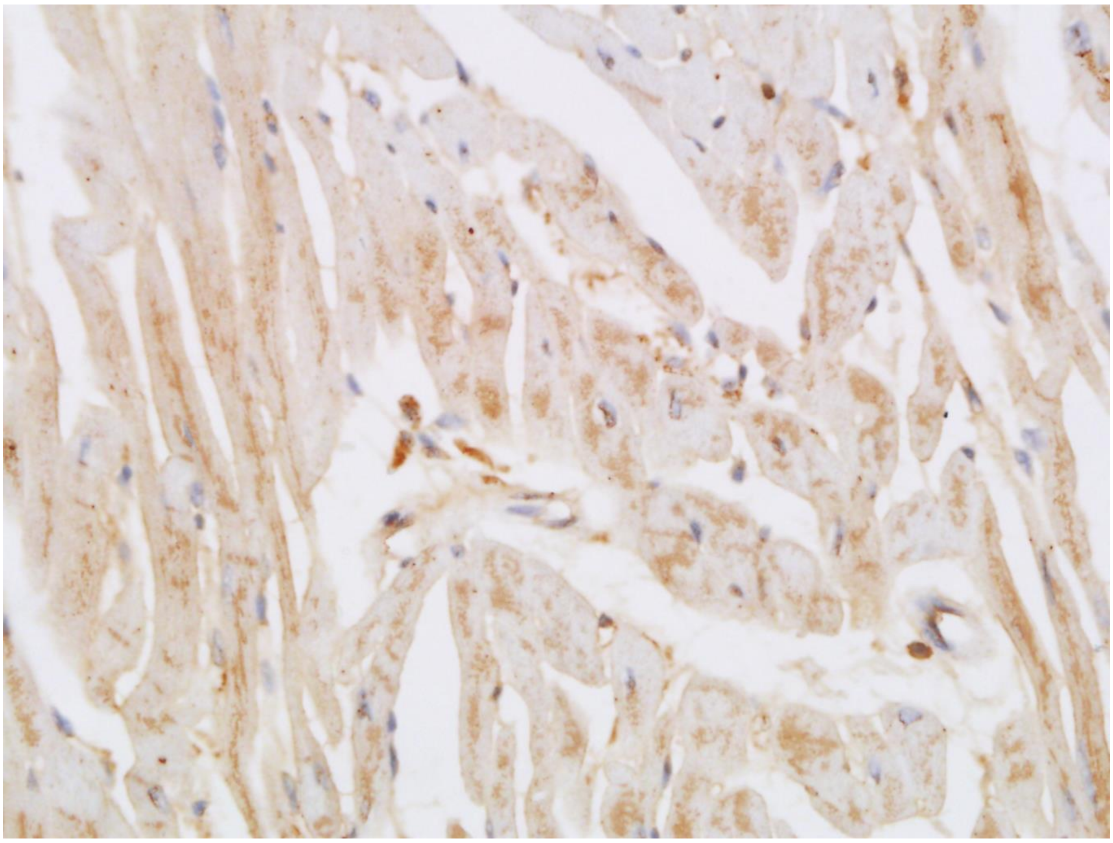

LV-SC-shRNA:

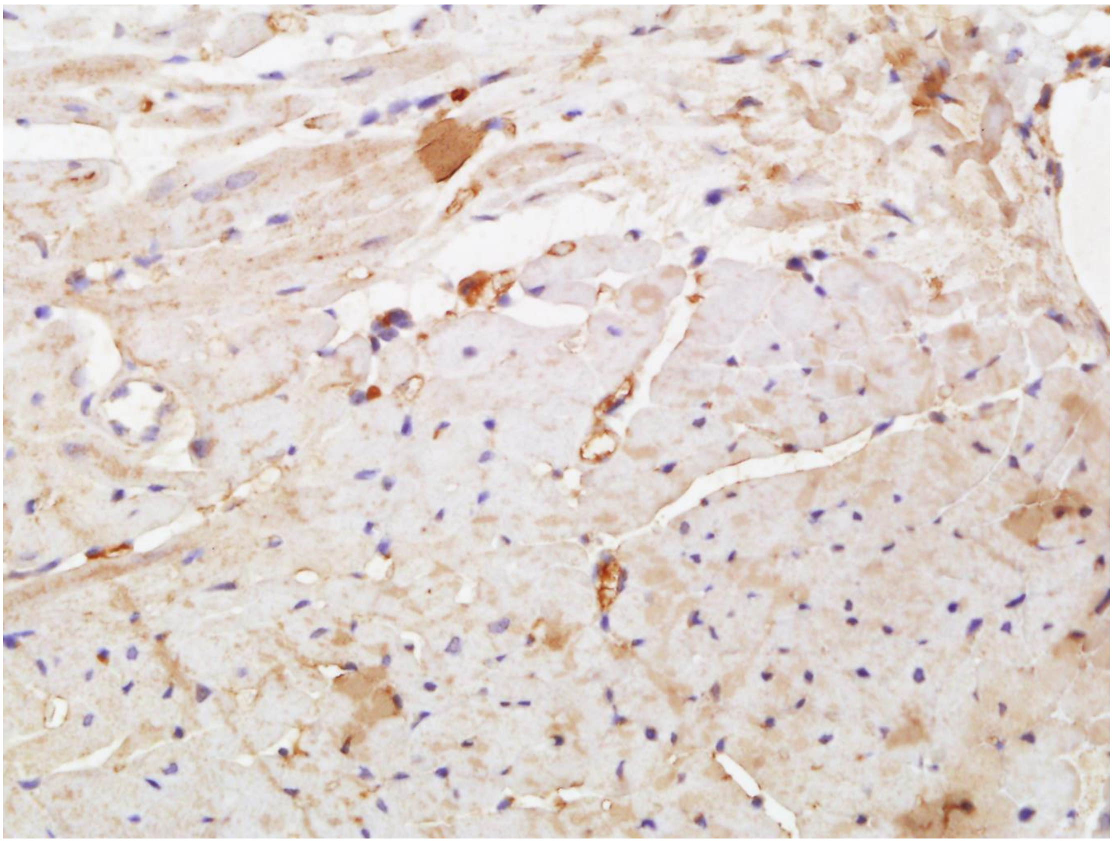

LV-YAP-shRNA

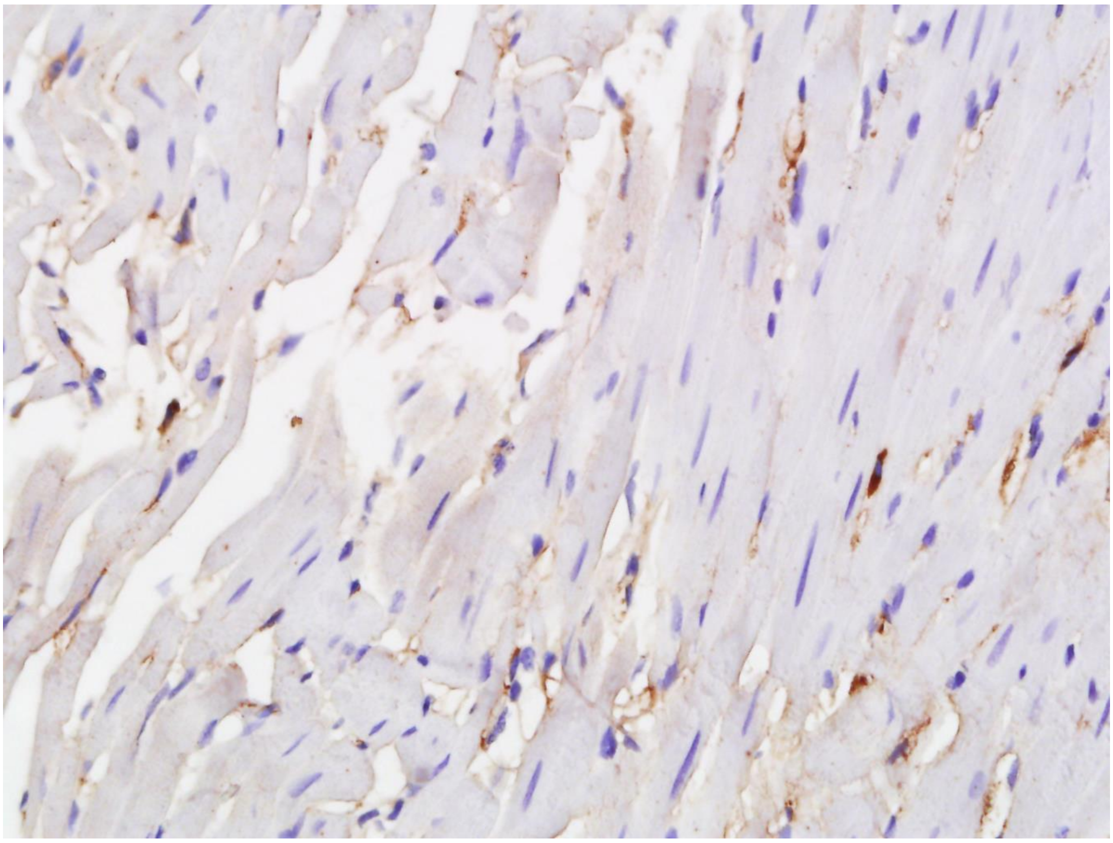

TNF-α
Control

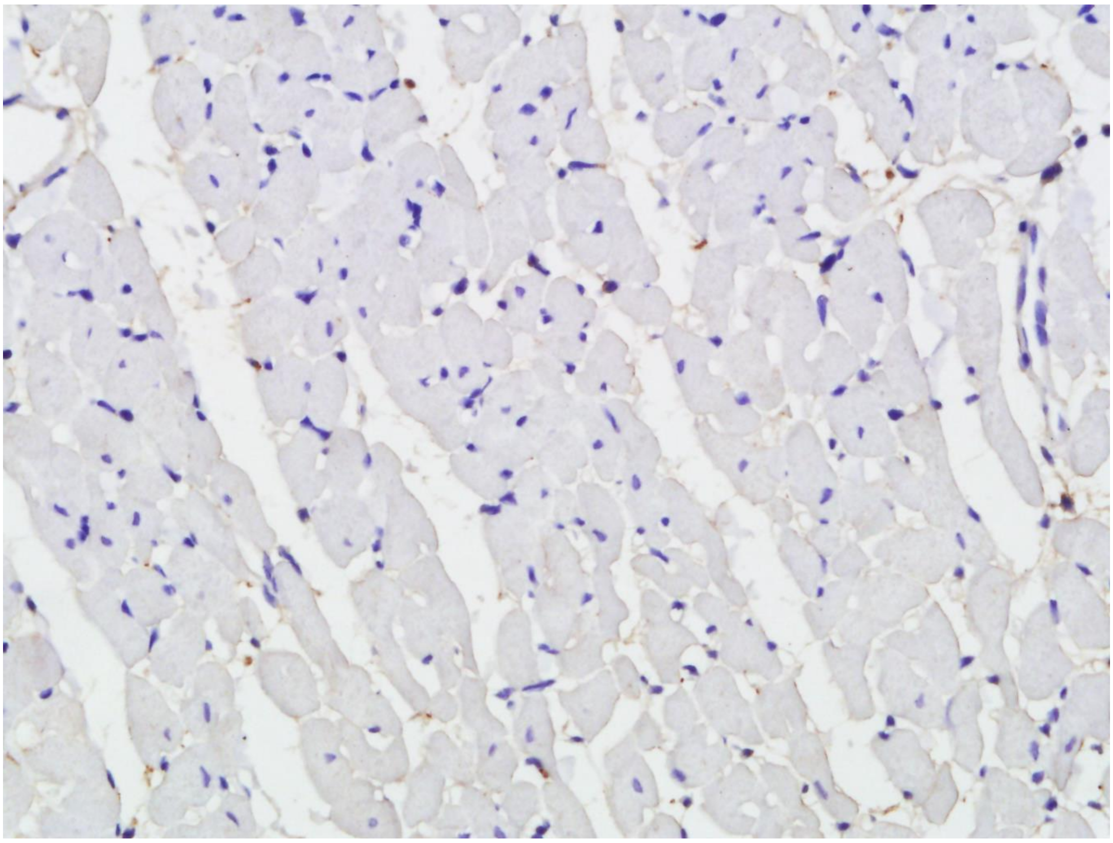

DCM

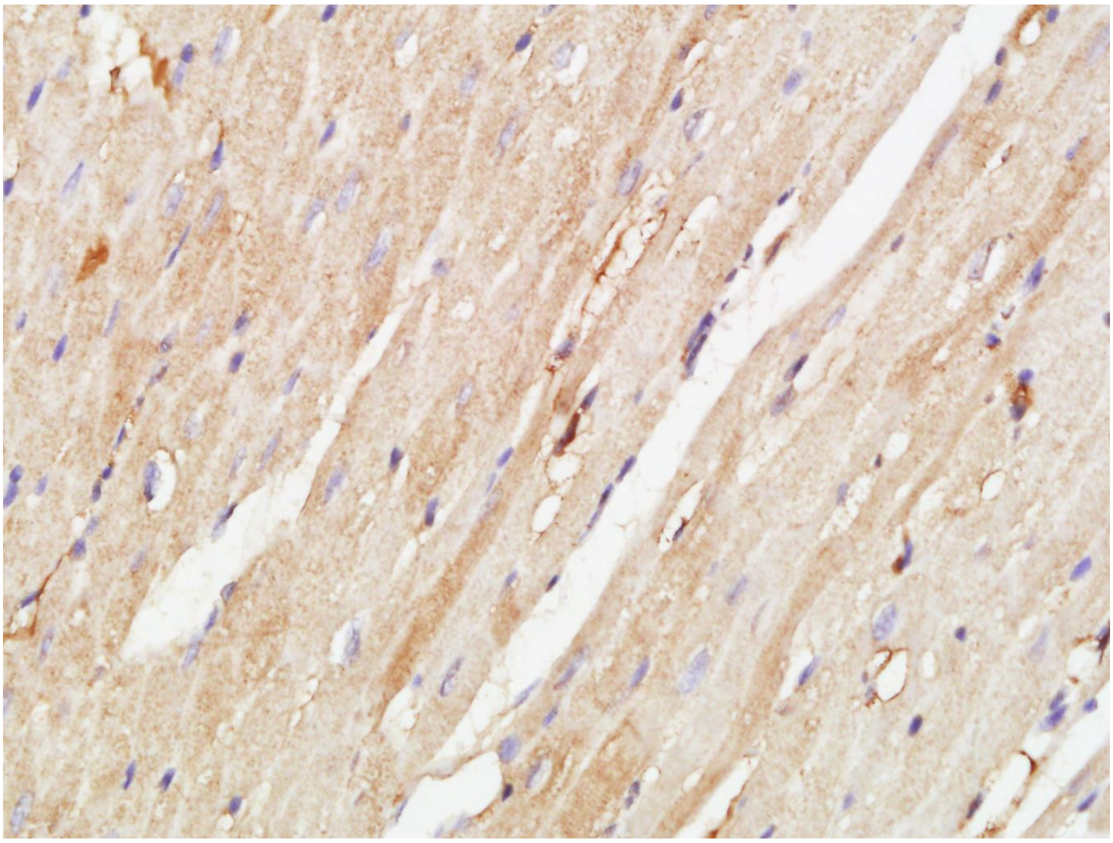

LV-SC-shRNA

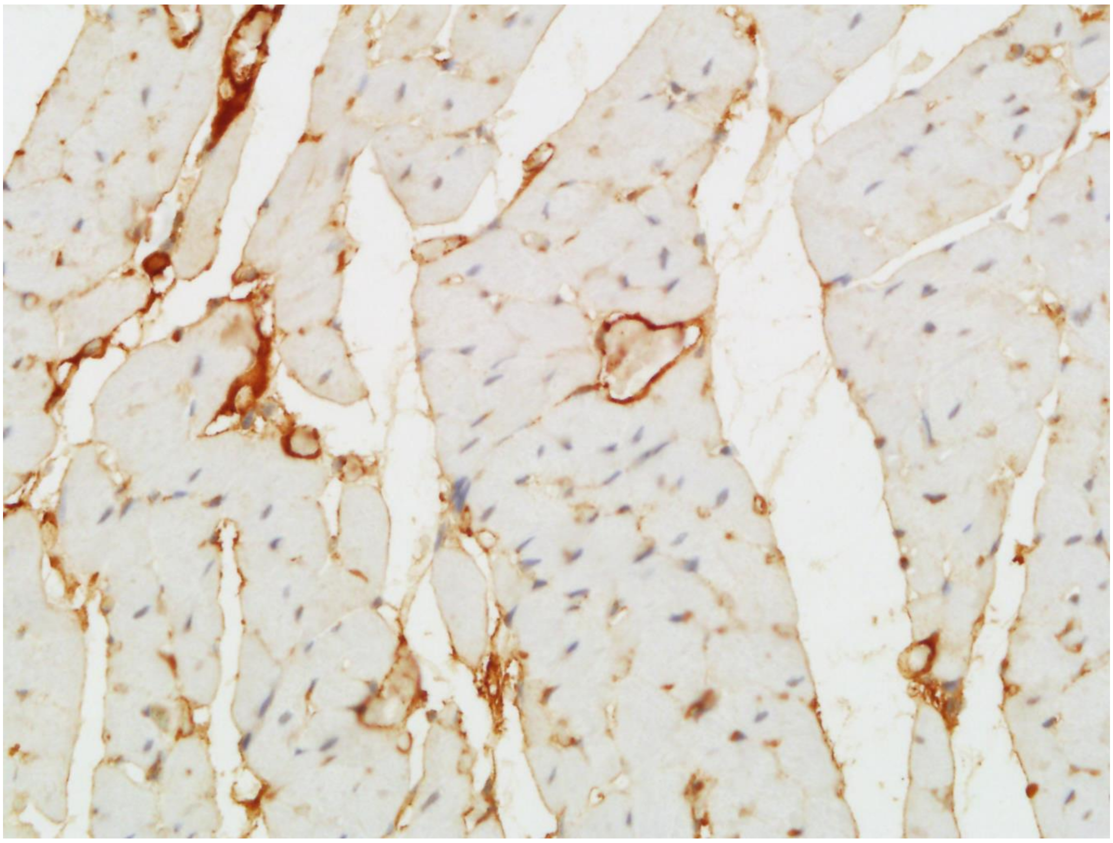

LV-YAP-shRNA:

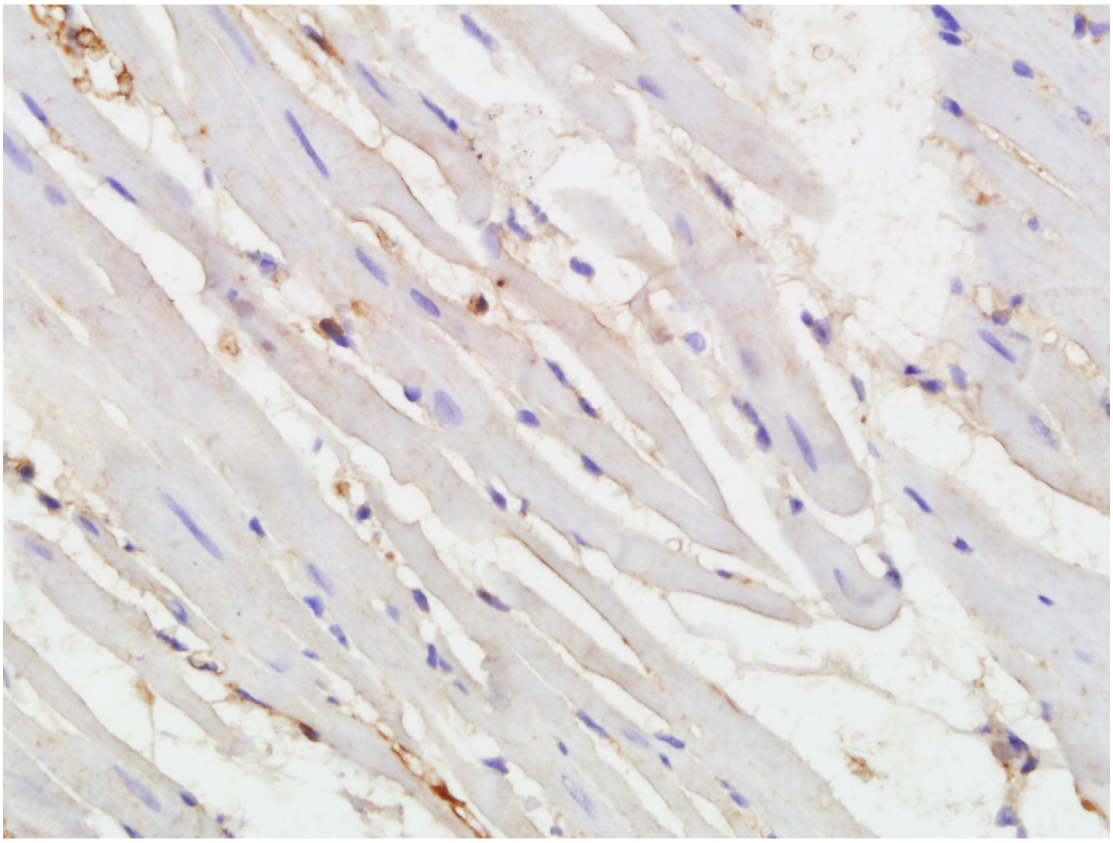

Figure5

YAP overexpression

Control

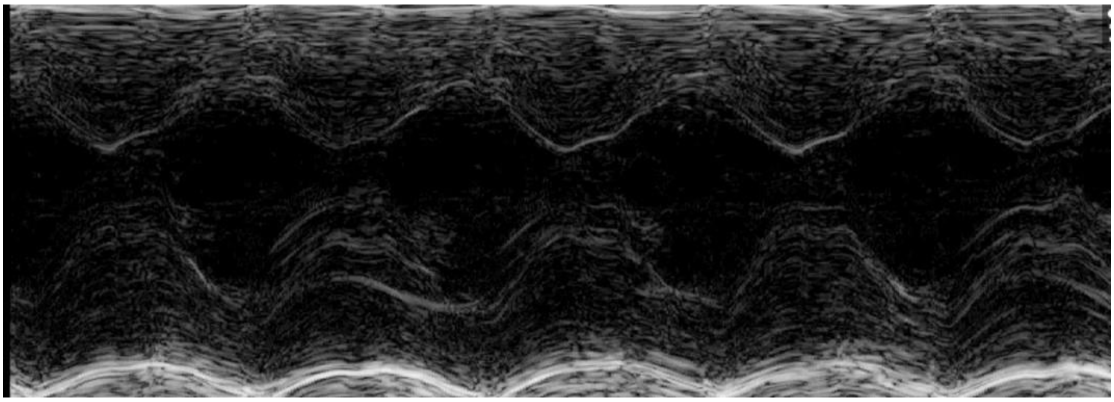

DCM:

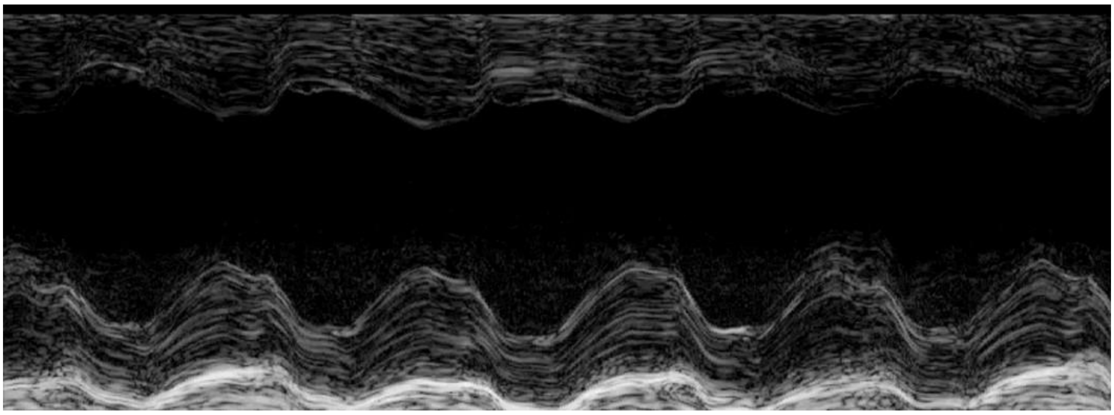

Ad-EGFP:

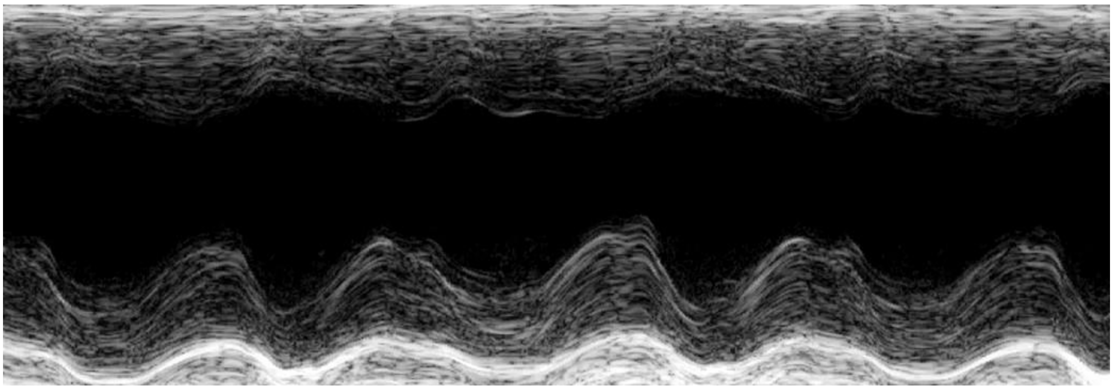

Ad-YAP:

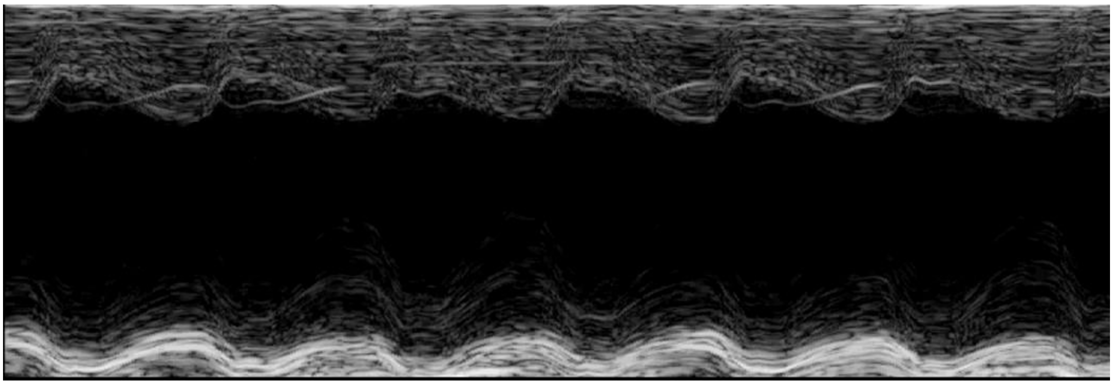

YAP silence

Control:

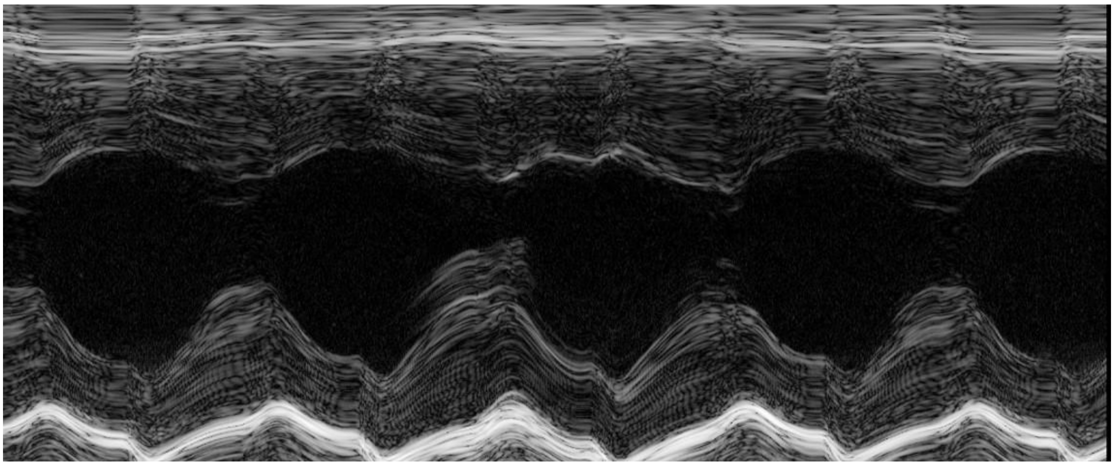

DCM:

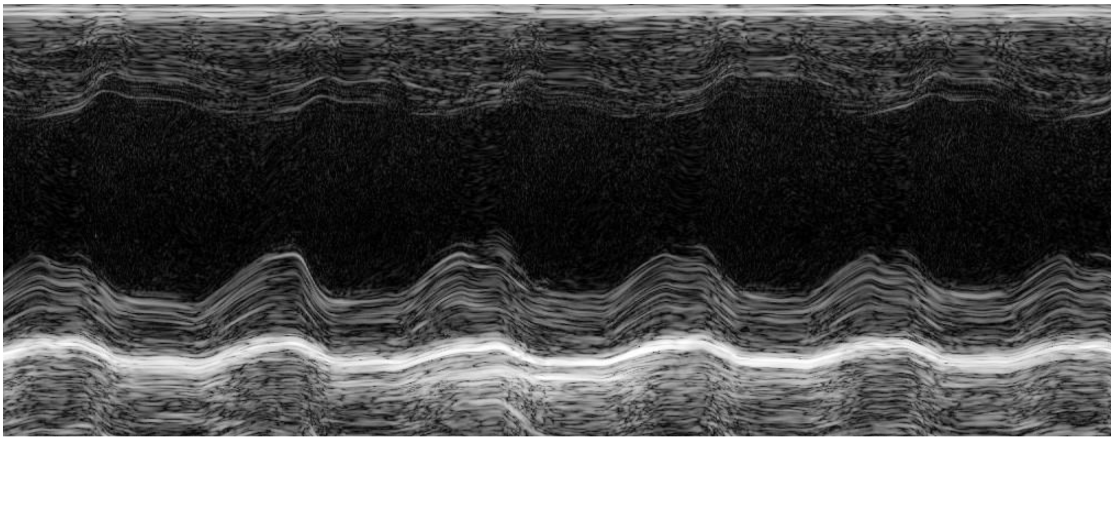

LV-SC-shRNA:

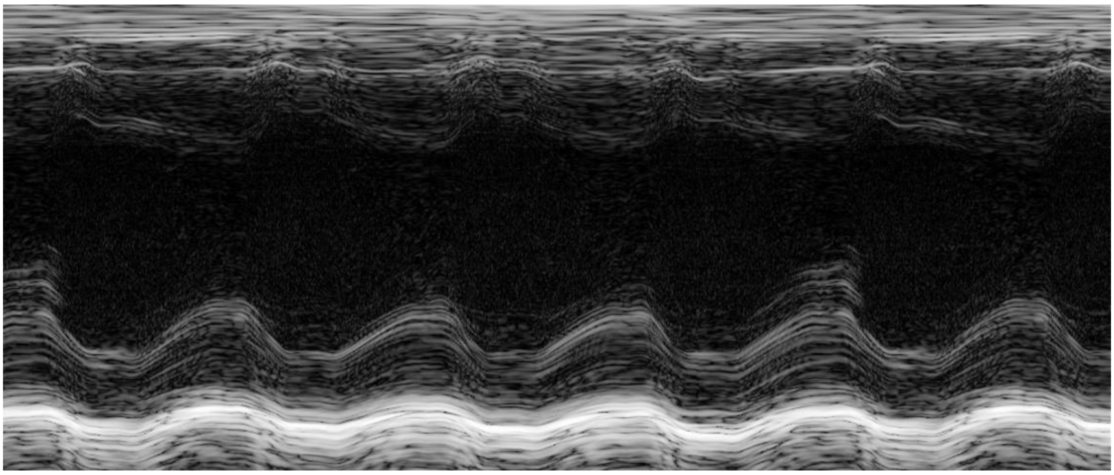

LV-YAP-shRNA:

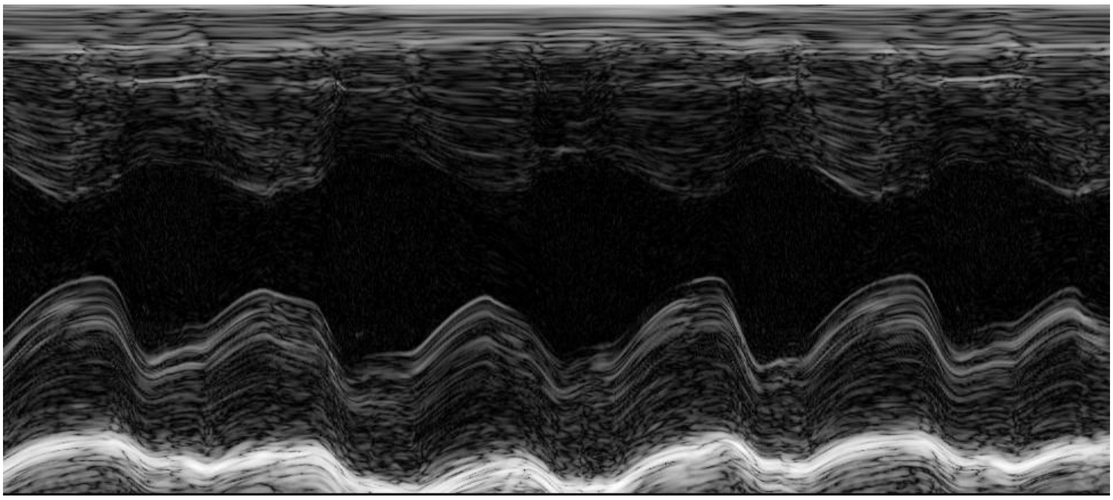

Supplement: Supplementary file 2 [file Data_Sheet_2.pdf]
